# The N-6 methyladenosine dynamics in STEMI and the effect of IL-6 inhibition - a hypothesis generating sub-study of the ASSAIL-MI trial

**DOI:** 10.3389/fimmu.2025.1532325

**Published:** 2025-06-06

**Authors:** Tuva B. Dahl, Ana Quiles-Jiménez, Kaspar Broch, Anne Kristine Anstensrud, Lars Gullestad, Geir Ø. Andersen, Ola Kleveland, Jonas Øgaard, Vigdis Bjerkeli, Azita Rashidi, Kuan Yang, Kirsten B. Holven, Pål Aukrust, Magnar Bjørås, Camilla Huse, Bente Halvorsen

**Affiliations:** ^1^ Research Institute of Internal Medicine, Oslo University Hospital Rikshospitalet, Oslo, Norway; ^2^ Department of Acute Medicine, Oslo University Hospital, Oslo, Norway; ^3^ Department of Cardiology, Oslo University Hospital Rikshospitalet, Oslo, Norway; ^4^ K. G. Jebsen Cardiac Research Centre and Centre for Heart Failure Research, University of Oslo, Oslo, Norway; ^5^ Faculty of Medicine, Institute of Clinical Medicine, University of Oslo, Oslo, Norway; ^6^ Department of Cardiology, Oslo University Hospital Ullevål, Oslo, Norway; ^7^ Department of Cardiology, Center for Clinical Heart Research, Oslo University Hospital Ullevål, Oslo, Norway; ^8^ Clinic of Cardiology, St. Olav’s Hospital, Trondheim University Hospital, Trondheim, Norway; ^9^ Norwegian PSC Research Center, Department of Transplantation Medicine, Division of Surgery, Inflammatory Diseases and Transplantation, Oslo University Hospital, Rikshospitalet, Oslo, Norway; ^10^ Department of Nutrition, Institute of Basic Medical Sciences, University of Oslo, Oslo, Norway; ^11^ National Advisory Unit on Familial Hypercholesterolemia, Department of Endocrinology, Morbid Obesity and Preventive Medicine, Oslo University Hospital, Oslo, Norway; ^12^ Department of Clinical and Molecular Medicine, Norwegian University of Science and Technology (NTNU), Trondheim, Norway; ^13^ Department of Microbiology, Oslo University Hospital, Oslo, Norway; ^14^ Department of Medicine, Cardiovascular Division, Brigham and Women’s Hospital, Harvard Medical School, Boston, MA, United States

**Keywords:** N6-methyladenosine (m^6^A), epitranscriptome, RNA methylation, STEMI, inflammation, tocilizumab

## Abstract

**Background:**

Epitranscriptomics, with m^6^A as the most prevalent in mammals, is a novel treatment target for inflammatory diseases, including cardiovascular diseases. However, little is known about m^6^A RNA-regulation during myocardial infarction (MI).

**Methods:**

In this explorative sub-study of the ASSAIL-MI trial, we used whole blood samples from patients with acute ST-elevation MI (STEMI) (n=6) at admission and after 3–7 days, and from healthy control subjects (n=3). RNA was isolated, and m^6^A sites were analyzed using human m^6^A single nucleotide resolution microarray analysis. mRNA levels were analyzed using RNA sequencing analysis.

**Results:**

Compared with controls, patients with STEMI had a strikingly different pattern of m^6^A deposition. In total, 845 m^6^A methylation sites in whole blood RNA were hypomethylated and 36 were hypermethylated compared with controls. Of the hypomethylated transcripts, 194 transcripts were lower expressed, while 197 transcripts were higher expressed. The m^6^A pattern changed from an overall hypomethylation at admission to an overall hypermethylation 3–7 day after admission. Anti-inflammatory treatment with tocilizumab further altered the m^6^A deposition.

**Conclusions:**

In this hypothesis generating study, m^6^A deposition differs STEMI patients and healthy controls. The m^6^A pattern changes over the course of 3–7 days. This response is, at least to some degree, is modulated by blocking the IL-6 receptor. Our data may suggest that this post-transcriptional regulation of RNA is involved in the immune response during STEMI, highlighting its potential as a target for therapy in MI.

## Background

Cardiovascular disease (CVD) is one of the foremost causes of mortality worldwide and is associated with large healthcare costs (1). Ischemic heart disease, including myocardial infarction (MI), is the most prevalent manifestation of CVD (1). Although survival after MI has improved, many patients have extensive myocardial damage and recurrent acute events, at least partly involving persistent inflammatory responses following MI (2). Percutaneous coronary intervention (PCI) has improved outcomes after MI, but is associated with ischemia/reperfusion injury that may further aggravate inflammation (2).

Patients with MI have localized as well as systemic inflammation (3). Inflammation within the myocardium behaves as a double-edged sword, and a correct immune response is crutial for the long term consequences. Some degree of inflammation is necessary for infarct healing, while an exaggerated and persistent response can be detrimental (4). In the ASSAIL-MI (ASSessing the effect of Anti-IL-6 treatment in Myocardial Infarction) trial we showed that mitigation of inflammation by blocking the interleukin-6 (IL-6) receptor with the monoclonal antibody tocilizumab leads to improved outcomes in patients with MI (5). Tocilizumab reduced C-reactive protein (CRP) and improved myocardial salvage (MSI) and the extent of microvascular obstruction in ST-elevation MI (STEMI) patients (5). In contrast to our previous study in NSTEMI patients (6), tociizumab did, however, not significantly reduce TnT levels as assessed as AUC during hospitalization in the STEMI study (5, 6).

The post-transcriptional RNA modification where methylation of the adenosine base at the nitrogen-6 position, forming N6-methyladenosine (m^6^A) RNA, is the most prevalent of the reversible epitranscriptomic modifications in mammals (7). This epitranscriptomic modification is shown to affect splicing, translation, stability, transcription level, and degradation of mRNA (8).

Evidence suggests a complex interplay between m^6^A deposition and inflammation in the pathogenesis of various diseases, including autoimmune diseases, cancers, and metabolic disorders (9). The m^6^A deposition is installed by methyltransferases, and new compounds affecting these enzymes shows promise in pre-clinical trials in cancer treatment (10). Data on m^6^A modification in CVD are scarce. In atherosclerosis, however, our research has indicated that the regulators of and the bulk levels of m^6^A are lower in RNA extracted from atherosclerotic carotid plaques than in RNA from healthy arteries (11). Reports suggest that several of the m^6^A methylation regulators are altered in MI, and that there is an interaction with immunity (12–16). The study by Yang J et al. is of particular interest, using data from the Gene Expression Omnibus (GEO) database to show that m^6^A modification, including its effects on the immune microenvironment, could play a key role in the pathogenesis of STEMI (12). In rodents, several of the m^6^A regulators may play a role in cardiac regenerative ability and heart function (17–23). A knockdown mouse model of the methyltransferase Wilms tumor 1 associated protein (WTAP), involved in m^6^A regulation, reduced hypoxia/reoxygenation-induced injury within the myocardium (24). Together, this underscore the potential for targeting this mechanism as a treatment target also in CVD.

To our knowledge, no studies have so far addressed the role of m^6^A methylation in whole blood from patients with STEMI, and data on how anti-inflammatory therapy modulates this methylation pattern are lacking. In this sub-study of the ASSessing the effect of Anti-IL-6 treatment in Myocardial Infarction (ASSAIL-MI) trial, we aimed to explore differences in m^6^A methylation sites between healthy individuals and patients with STEMI at time of hospitalization, and how this affected gene expression of the targeted mRNA. We also aimed to explore the alterations in m^6^A methylation 3–7 days after hospitalization compared with hospitalization, and the influence of the IL-6 receptor blocker tocilizumab on m^6^A methylation in STEMI.

## Methods

### Patients and study design

This study comprises six subjects enrolled in the ASSAIL-MI trial and three healthy control subjects. These analysis were a sub-study of the ASSAIL-MI trial, registered in ClinicalTrials.gov, number NCT03004703. The demographics of the patients and controls in this sub-study are shown in **Table 1**. All controls were characterized as healthy based on the disease history and no use on regular medication.

**Table 1:** Baseline characteristics of the patients and healthy controls included in the m^6^A single nucleotide array.

Baseline Characteristics	Hospitalized STEMI (*n*=6)	Healthy (*n*=3)
Age, years	63 ± 7	59 ± 4
Men	2 (33,3)	2 (66)
Time from symptom onset to arrival at PCI center, min	144 ± 45	

Values are mean ± SD or n (%).

The ASSAIL-MI trial investigated whether a single intravenous dose of tocilizumab could improve myocardial salvage in patients admitted with acute STEMI. The key inclusion criterion was first-time STEMI with symptom onset less than 6 hours before PCI. Patients were excluded if they had previous MI; chronic infection, or chronic inflammatory or autoimmune disease; uncontrolled inflammatory bowel disease; ongoing infectious or immunologic disease; major surgery within the past eight weeks; or treatment with immunosuppressants other than low-dose steroids (equivalent to a systemic exposure to 5 mg prednisone per day). In addition, all patients in both treatment arms were treated according to current established guidelines for STEMI patients. The exclusion criteria of no previous MI mean that the data should be interpreted with caution in relation to patients with recurrent MI. Details about the study design and participants have been described elsewhere (5, 25). The trial participants were allocated 1:1 to treatment with tocilizumab 280 mg i.v or matching placebo in a double-blind manner. Immediately after randomization and the initiation of study drug administration, patients underwent PCI of the culprit vessel and provided optimal standard medical therapy (5, 25).

Explorative sub-studies on inflammation were pre-specified in the originally approved study protocol (25). Although the present sub-study could fits into this category, the analyses of m^6^A were not predefined.

### Blood sampling protocol

Whole blood samples for total RNA isolation were collected in PAXgene™ Blood RNA tubes (BD, Franklin Lakes, NJ). Arterial samples were taken at admission (prior to PCI, before unfractionated heparin and tocilizumab/placebo were administered at the catheterization laboratory). Venous samples were drawn after 3–7 days. Venous blood samples were collected once only from the healthy control subjects.

### RNA isolation and sequencing

We have previously published the main results of RNA sequencing from whole blood in the ASSAIL-MI trial (26). Total RNA was isolated from the BD PAXgene™ Blood RNA samples with the MagMAX™ for Stabilized Blood Tubes RNA Isolation Kit (Invitrogen™, Waltham, MA) following the manufacturer’s instruction. Novogene (UK) Company limited used a ribosomal RNA depletion library on the isolated RNA samples. The fastp (v0.23.0) was used to remove contaminated adapters and low-quality reads with phred score below 30 in the pair-end mode (27). Filtered reads were mapped to the human transcriptome (Gencode Human Release H37), and transcripts were quantified with 200 bootstrap iterations by Salmon (v1.5.2) (28, 29). The Salmon outputs were summarized to gene-level and imported into DESeq2 (v1.34.0) via tximeta (v.1.12.3) (30, 31). For better accuracy, hemoglobin mRNAs were removed before the analysis of differentially expressed genes (DEGs) (32). DEGs were uploaded to Metascape for pathway analyses.

### m^6^A single nucleotide array

m^6^A sites were analyzed using human m^6^A Single Nucleotide resolution microarray analysis by Arraystar Inc (Rockville, MD, USA). We used Nanodrop ND-100 for total RNA quantification and Bioanalyzer 2100 and Mops electrophoresis to control RNA integrity. Arraystar’s standard protocols were used for sample preparation and microarray hybridization. Briefly, the total RNA was split into two fractions: “MazF-digested” and “MazF-Undigested”. The “MazF-Digested” fraction was treated with the RNA endoribonuclease MazF to cleave unmodified m^6^A sites. The “MazF-Undigested” fraction was not treated with MazF for both modified and unmodified sites. The “MazF-Digested” RNAs were labeled with Cy5, and the “MazF-Undigested” RNAs were labeled with Cy3 as cRNAs in separate reactions using Arraystar RNA Labeling protocol. The two cRNA fractions were then combined and hybridized onto Arraystar Human m^6^A Single Nucleotide Array (8x15K, Arraystar). The slides were washed and scanned in two-color channels by an Agilent Scanner G2505C.

Agilent Feature Extraction software (version 11.0.1.1) was used to analyze the acquired array images. The average of log2-scaled Spike-in RNA intensities was used for normalization of the raw intensities of MazF-Digested (Cy5-labelled) and MazF-Undigested (Cy3-labelled) RNA. Then, probe signal with Present (P) or Marginal (M) QC flags in at least 3 out of 15 samples were retained for “m^6^A site abundance” analyses. The “m^6^A site abundance” was calculated for the m^6^A methylation amount based on the normalized intensities of the MazF-Digested (Cy5-labelled) samples. Differentially m^6^A-methylated sites between the groups for comparisons were identified by filtering on the fold change (FC) and statistical significance (p-values) thresholds. The data was also filtered based on m^6^A methylation site position in mRNAs, and further into the 5’UTR, CDS, and 3’UTR regions. The default thresholds were |FC| ≥ 2.0 and p-values < 0.05. To show the distinguishable m^6^A-methylation pattern among samples, hierarchical clustering was performed. All m^6^a modifications are adjusted for the total number of transcript.

### Statistics

For the RNA-sequencing data, we performed false discovery rate (FDR) adjustment and report adjusted *p*-values. It is important to notice that patient number (given as *n*) varies slightly at different time-points for RNA analyses due to quality issues of missing samples. However, the amount of missing data was evenly distributed between the placebo and tocilizumab groups, and the missing values are assumed to be missing at random. mRNA transcript counts from genes involved in m^6^A regulation were analyzed using *t*-test and 2-way ANOVA, and *p*-values < 0.05 were considered statistically significant. Statistical analyses were performed in GraphPad Prism 8.3.0 (GraphPad Software, La Jolla, CA).

## Results

**Figure 1:**
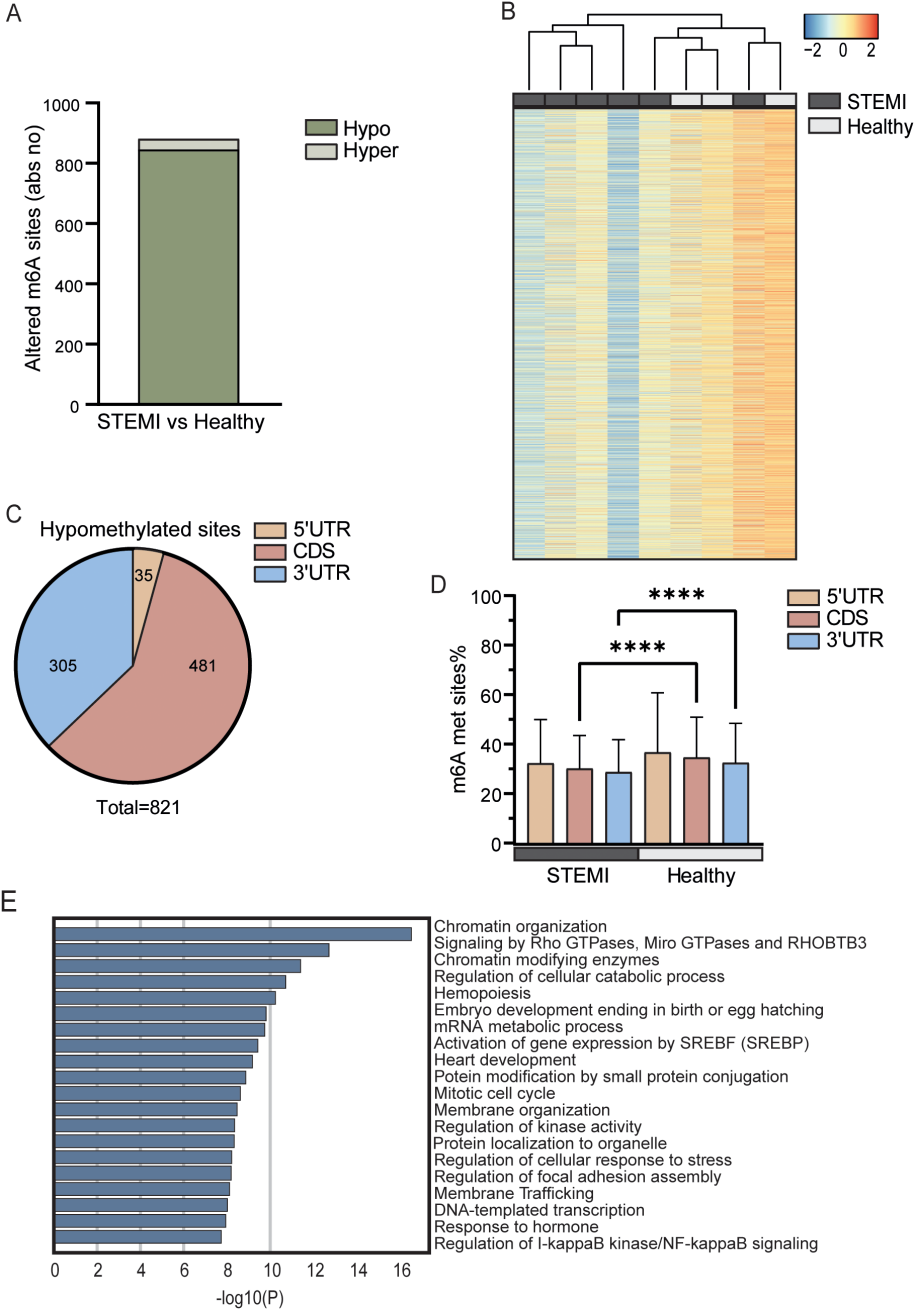
m^6^A distribution in patients with STEMI prior to PCI vs healthy controls. **(A)** Absolute numbers of significantly hypo- or hypermethylated m^6^A sites in total RNA from whole blood between patients with STEMI at hospitalization and healthy controls. **(B)** Heatmap showing the degree of m^6^A methylation in total RNA between the groups. **(C)** Distribution of regulated hypomethylated m^6^A sites in mRNAs between patients with STEMI at hospitalization and healthy controls. **(D)** Average m^6^A methylation percent for all sites in the different sections of protein coding transcripts. ****p < 0.0001 (2-way ANOVA with Tukey’s multiple comparisons test). **(E)** Metascape analysis of GO biological processes and Reactome pathways for all significantly differentially methylated mRNAs.

Of the 11,237 possible m^6^A sites analyzed in the array, whole blood RNA from patients with acute STEMI had at hospital admission 845 hypomethylated m^6^A sites and 36 hypermethylated sites, as compared with RNA from whole blood from healthy controls (**Figure 1A**). The transcript with the highest fold change in the hyper methylated sites is the HSP90AA1, coding for the stress induced heat shock protein Hsp90A (33), while the transcript with the highest fold change of the hypomethylated sites is an methyltransferase forming m7A, METTL1 (34) (**Supplementary Table S1**). Heatmap cluster analysis of all analyzed m^6^A sites partly separated patients with acute STEMI from healthy controls (**Figure 1B**). We found that most m^6^A hypomethylation in mRNAs occurred in the coding sequence (CDS) and the 3’ untranslated region (UTR), while less than 10% of the sites were found in the 5’UTR (**Figure 1C**). In contrast, the hypermethylated sites comprised of only 20 sites in the CDS and 14 sites in the 3’UTR (**Supplementary Figure S1**). As shown in **Figure 1D**, the average degree of m^6^A methylation was significantly lower in both CDS and 3’UTRs, but not in 5UTR, in patients with STEMI compared with healthy controls (p < 0.0001 for both CDSs and 3’UTRs, **Figure 1D**). Although the 3’UTR of the mRNA transcripts has regulatory functions important for mRNA stability, localization, and translation (35).

Annotation analysis of mRNAs with differentially enriched m^6^A sites (both hyper- and hypomethylated) showed that “chromatin organization” (GO: 0006325) was the most significantly regulated pathway between the patients with STEMI and the healthy controls (**Figure 1E** **).** Intriguingly, recent studies suggest that dysfunction in chromatin regulators may be an important mechanism of MI (36). Other pathways relevant for the processes during STEMI, potentially influenced by m^6^A, included “signaling by Rho GTPases, Miro GTPases and THOBTB3” (R-HSA-9716542), “hemopoiesis” (GO:0030097), and “regulation of I-kappaB kinase/NF-kappaB signaling” (GO:0043122). All these pathways are important for a correct immune responses and inflammation during STEMI. It is, however, important to underscore that all pathways in **Figure 1E** is significantly regulated at m^6^A methylation sites. This means that these pathways is potentially regulated by this RNA modification, but not necessarily the most relevant pathological pathway in MI and we should avoid grading of the biological importance of the different pathways based on p value alone.

**Figure 2:**
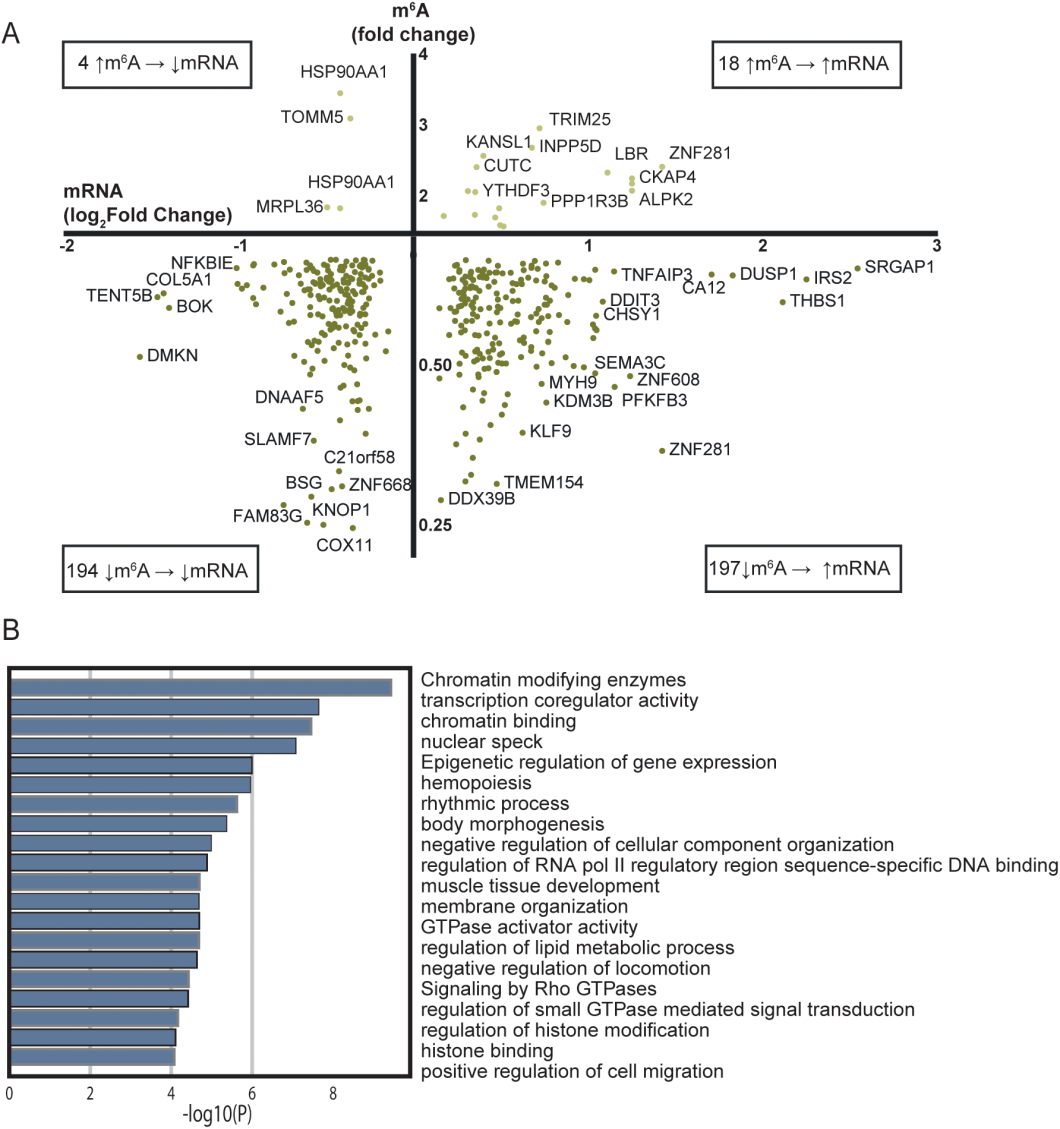
Comparison of RNA sequencing and m^6^A array for all mRNAs with significantly regulated m^6^A sites between patients with STEMI at hospitalization and healthy controls. **(A)** Transcripts with altered m^6^A methylation and their transcript level between patients with acute STEMI and healthy controls. Only genes/targets found in both analyses are included. **(B)** Metascape analysis of GO biological processes and Reactome pathways for the 197 hypomethylated and upregulated genes in **(A)**.

To examine whether m^6^A methylation in patients with STEMI had an impact on the mRNA levels, we compared the fold difference of m^6^A methylation to log_2_fold difference of the corresponding mRNA levels (**Figure 2A**, **Supplementary Table S3**). Notably, we observed that 48.3% of the differentially methylated transcripts were also differentially expressed between the two groups. Of the hypomethylated transcripts, 194 transcripts were down-regulated while 197 transcripts were up-regulated in the patients with STEMI than in the healthy controls. For the hypermethylated transcripts, 4 mRNA transcripts were down-regulated and 18 mRNA transcripts were up-regulated.

m^6^A is suggested to be a mark for degradation of the mRNA (37), suggesting that less m^6^A will lead to a higher amount of the mRNA in question. The 197 hypomethylated mRNAs that were associated with higher mRNA transcript levels in **Figure 2A** follow this pattern. To assess the biological function of the genes coding for these mRNAs, we performed an additional annotation analysis (**Figure 2B**). “Chromatin modifying enzymes” (R-HSA-3247509) was the most significant, but again we should avoid grading of the importance of the different pathways based on p value alone. However, several pathways were related to myocardial injury and healing, such as “hemopoiesis” (GO:0030097), “positive regulation of cell migration” (GO:0030335), and “regulation of lipid metabolic process” (GO:0019216) (38–40) were also altered and could therefore be regulated trough the m^6^A methylation pathway.

**Figure 3:**
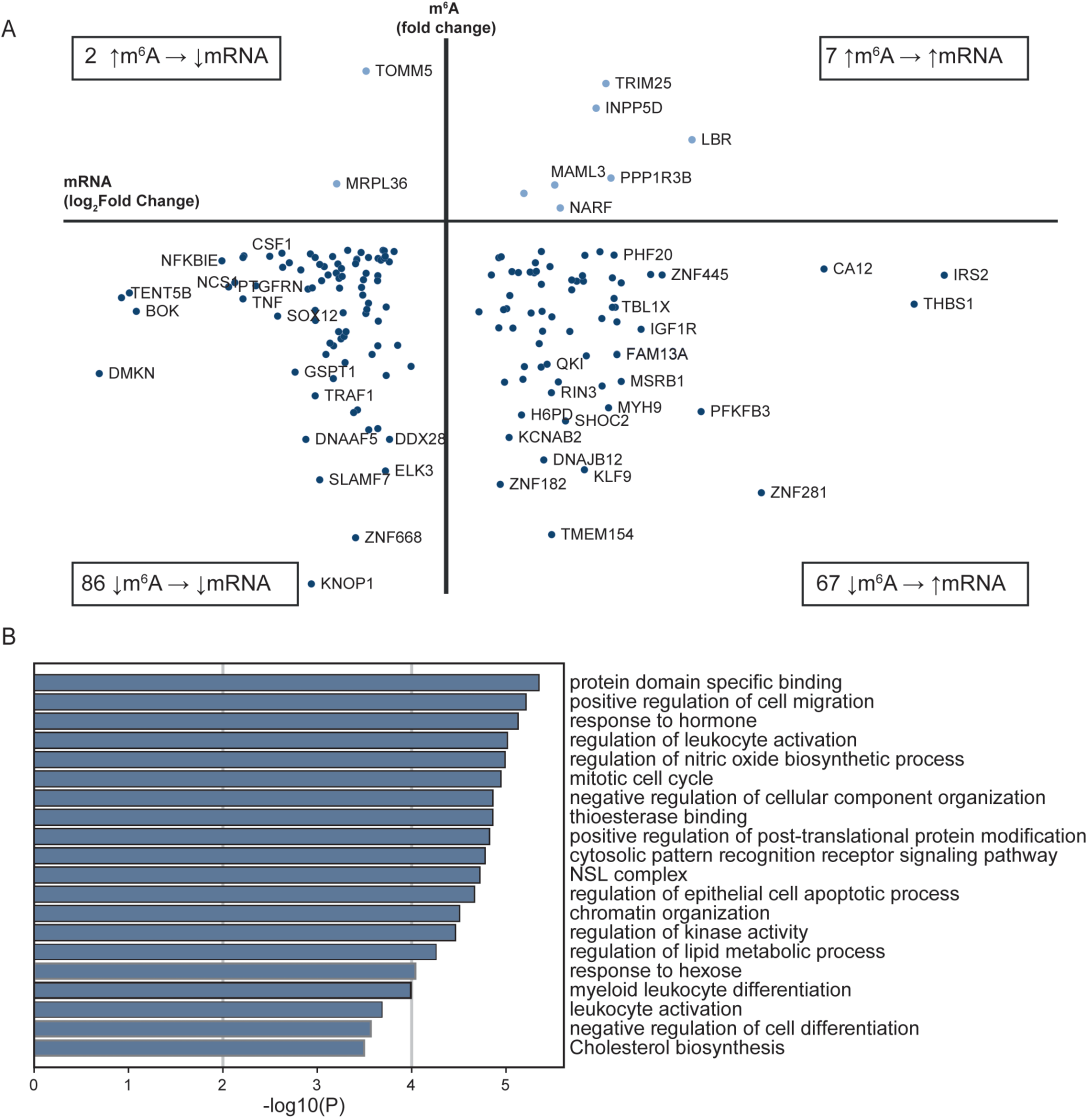
Comparison of RNA sequencing and m^6^A array for all transcripts with altered m^6^A methylation in the 3’UTR between patients with STEMI at hospitalization and healthy controls. **(A)** Transcripts with differently altered m^6^A sites in the 3’UTR and their transcript levels between patients with acute STEMI and healthy controls. Only genes found in both analyses are included. **(B)** Metascape analysis of GO biological processes and Reactome pathways for all 3’UTR genes in **(A)**.

The 3’UTR of the mRNA transcripts has regulatory functions important for mRNA stability, localization, and translation (35). 86 mRNAs were hypomethylated in the 3’UTR and were expressed at a lower log_2_fold level in patients with STEMI then in healthy controls, while 67 hypomethylated mRNAs were expressed at a higher log_2_fold level in STEMI (bottom quadrants, **Figure 3A**). A small proportion of the genes were hypermethylated at the 3’UTR in STEMI versus healthy controls, with log_2_fold transcript levels lower in 2 and higher in 7 of the transcripts (upper quadrants, **Figure 3A**).

Annotation analysis of the transcripts with altered m^6^A methylation in the 3’UTR, revealed that the most significantly altered metabolic pathways were “protein domain specific binding” (GO:0019904) and “positive regulation of cell migration” (GO:0030335) (**Figure 3B**).

**Figure 4:**
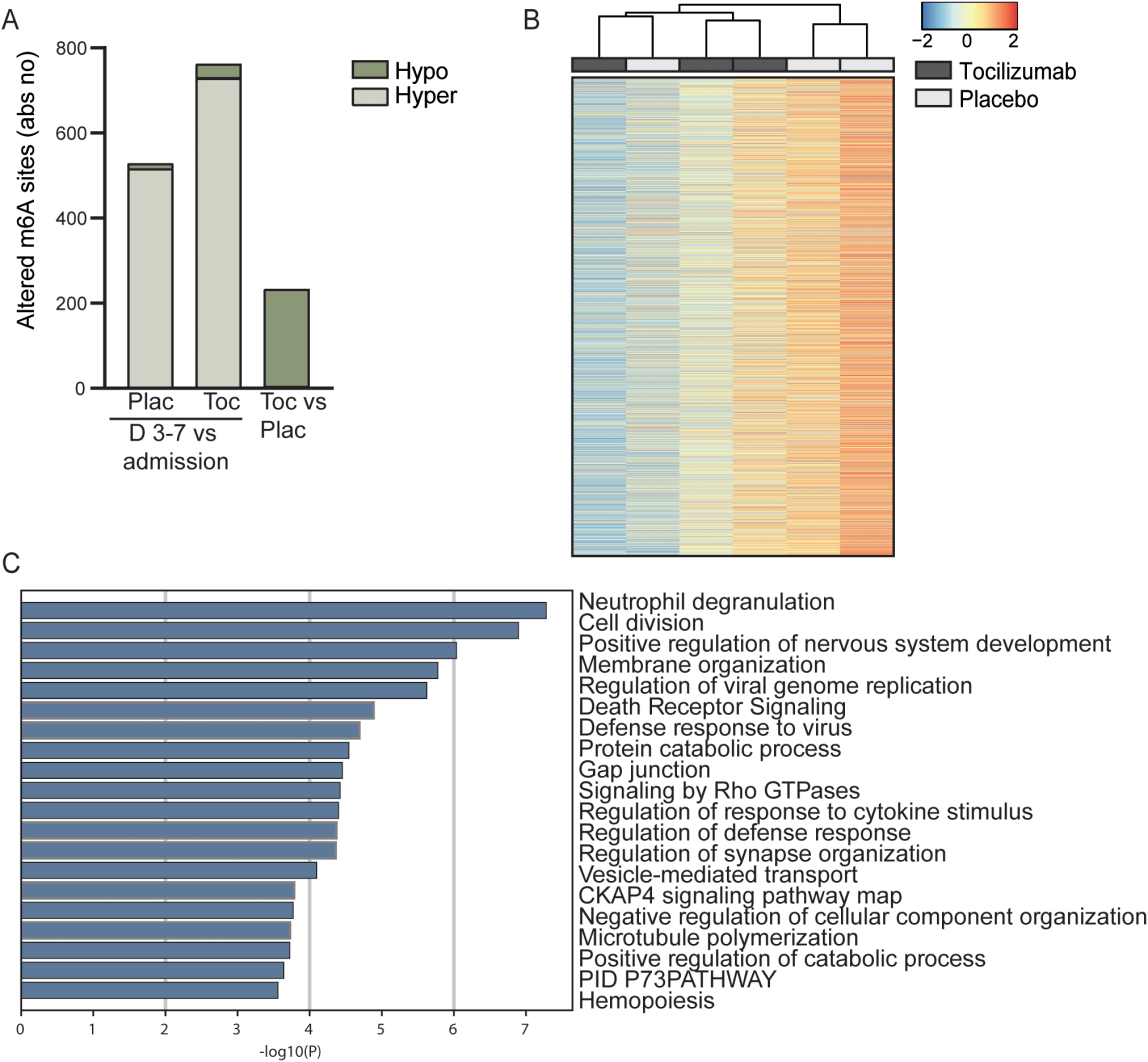
Longitudinal m^6^A distribution in patients with STEMI and the effect of IL-6 receptor inhibition. **(A)** Absolute numbers of significantly hypo- or hypermethylated sites in total RNA from whole blood 3–7 days after percutaneous coronary intervention (PCI) treatment, versus time at hospitalization for placebo treated (Plac), for patients receiving tocilizumab (Toc) and tocilizumab vs placebo at day 3-7 (Toc vs Plac). **(B)** Heatmap showing the degree of m^6^A methylation in total RNA in the placebo and tocilizumab group 3–7 days after treatment. **(C)** Metascape analysis of GO biological processes and Reactome pathways for all significantly differentially methylated mRNAs between placebo and tocilizumab treated patients.

The ASSAIL-MI trial participants were randomized to 280 mg tocilizumab or placebo prior to revascularization by PCI. In both the placebo and tocilizumab group, there was a marked shift in the m^6^A methylation pattern from hospital admission to 3–7 days after admission. There was a shift to increased hypermethylation (516 sites and 729 sites; placebo and tocilizumab, respectively) and decreased hypomethylation (12 sites and 33 sites; placebo and tocilizumab, respectively) compared with what was observed at the time of hospital admission (**Figure 4A**, **Supplementary Table S2**). At day 3-7, 3 m^6^A sites were significantly hypermethylated and 230 m^6^A sites were significantly hypomethylated in the tocilizumab arm versus the placebo arm (**Figure 4A**). Heatmap cluster analysis of all methylated sites in whole blood RNA from the placebo and tocilizumab treated groups showed incomplete separation of the two treatment arms (**Figure 4B**).

Hypermethylated sites in protein-coding mRNA in the placebo arm were mostly positioned in the 3’UTR (200 sites) and the CDS (284 sites) (**Supplementary Figure S2**). A similar pattern was observed in the tocilizumab arm (CDS 423 sites and 3’UTR 264 sites) (**Supplementary Figure S3**). The average methylation percent for all sites was higher for 5’UTR, CDS, and 3’UTR after 3–7 days for both treatment arms than at the time of hospitalization (**Supplementary Figures S2, S3**). There were no differences between the treatment groups regarding the average m^6^A methylation percent for 5’UTR, CDS, or 3’UTR (**Supplementary Figure S4**).

Annotation analyses comparing transcripts with significantly regulated m^6^A sites (both hyper- and hypomethylated) between tocilizumab and placebo showed that the pathway that was regulated most differently between the treatment arms was “Neutrophil degranulation” (R-HSA-6798695). This pathway is highly relevant for the immune response after STEMI and IL-6 inhibition by tocilizumab as also shown in the ASSAIL-MI trial (26). Amongst the differently methylated sites we find the transcripts for CD14 and Toll like reseptor 2 (TLR2), two protein shown to be important for priming the neutrophils in response granulocyte macrophage colony stimulating factor (GM-CSF) (41). Although the authors tested these mechanisms in responses på pathogen associated molecular pattern, we believe that similar mechanisms will be operating in response to danger associated molecular pattern such as during STEMI. Further, other relevant methylated transcripts for Disintegrin and metalloproteinase domain-containing protein 10 (ADAM10) and CSTB, which induce production of cystatin B, are both related to extracellular matrix remodeling (42, 43), and Fibrinogen Like 2 (FGL2) is related to formation of neutrophil extracellular traps (NETs) (44). Other interesting pathways related to the immune system and cell maintenance that were regulated differently between the two treatment arms were “Response to cytokine stimulus” (GO:0060759) and “Membrane organization” (GO:0061024) (**Figure 4C**).

Finally, we reanalyzed previous published transcriptome analyses on enzymes involved in in m^6^A regulation from whole blood in the ASSAIL trial which is the basis of the present manuscript (26). Transcriptome data were available 14 healthy controls, 37 STEMI patients at hospital admission and of these patients 19 received tocilizumab and 18 received placebo during follow-up. As shown in **Supplementary Figure S5**, STEMI patients had decreased transcript levels of the writer METTL16 and increased levels of the writer WTAP, and an even more complex regulation of the readers with up-regulation of YTHDF3 and PRRC2A and down-regulation of YTHDF1, YTHDF2 and IGF2BP2 in STEMI patients. Moreover, analyses of samples collected 3–7 days after hospital admission showed an up-regulation of the readers METTL16 and RBM15 and a down-regulation of the writers YTHDF3 and HRNPC in the tocilizumab group (**Supplementary Figure S6**). The regulation of m^6^A by these enzymes are complex (7) and the net effects of these changes are at present uncertain. These data, at least in some degree, support changes of the m^6^A regulating machinery in STEMI and notably, some of these changes (regulation of METTL3 and YTHDF3) were reversed by tocilizumab.

## Discussion

Very recently, Chao et al. showed data on the m^6^A regulators during MI (45). Moreover, data from Yang J e al suggest that m^6^A modification could contribute to the pathogenesis of STEMI, including effects on the myocardial microenvironment (12). Furthermore, a recent review summarized clinical and preclinical data, supporting a role of m^6^A modification in aterogenesis, ischemia-reperfusion injury and MI (46). To the best of our knowledge, however, no reports have previously described the m^6^A methylation landscape over time in patients with STEMI. In this explorative sub-study of the ASSAIL-MI trial, we found that these patients had an m^6^A pattern that was strikingly different from that of healthy controls, with a general hypomethylation of transcripts in patients with STEMI. Intriguingly, the same patients showed an overall hypermethylation 3–7 days after hospitalization and PCI treatment compared with at admission. Despite similarities between the placebo and the tocilizumab group, anti-inflammatory treatment with tocilizumab altered m^6^A deposition after STEMI.

At hospital admission, the patients with STEMI had less m^6^A methylation than healthy controls. The distinctly different profile of m^6^A methylation could reflect m^6^A distribution as a participant in the regulation of the immune response during STEMI. Vausort et al. showed that patients who developed heart failure after MI had lower levels of m^6^A in the blood (47). This, in addition to our results, points to that m^6^A levels might play important roles in the immune response to MI, and could possibly also predict outcomes after an MI.

We found that over all, our patients with acute STEMI had less m^6^A methylation (i.e., more hypomethylation) than healthy controls. On the other hand, a study on peripheral blood mononuclear cells from patients with STEMI, non-STEMI, and unstable angina showed increased m^6^A methylation (i.e., more hypermethylation) in all three conditions (16). This study did not include neutrophils, the most dominating cell type in whole blood, playing an important role in acute MI (26). The discrepancy between these results might therefore reflect that different immune cell subtypes in the blood have different m^6^A profiles during MI.

Mo et al. have shown that m^6^A-single nucleotide polymorphisms, which can result in gain or loss of the m^6^A methylation site, are associated with coronary artery disease (CAD) (48). It is therefore likely that m^6^A methylation might play a causal role in the development of this disorder. Accordingly, m^6^A methylation could be a target for therapy in CAD and potentially also other forms of CVD (48), as suggested in cancer (49). Indeed, it has also been suggested that m^6^A could represent a novel target for therapy in MI (46).

It is important to underscore that the effect of m^6^A on RNA is rather complex affecting both stability, clearance, splicing and translation (50). Furthermore, whereas hypometylation is thought to enhance processes such as induction of RNA stability and increased translation, the opposite may be a consequence of hypermethylatin transcripts (50). Herein we found that in general, hypomethylation was more closely related to alteration in the transcript level, than hypermethylation, but with no clear differences in the number of up-regulated or down-regulated transcripts, underscoring a complex regulation of transcript levels by m^6^A modification. The mRNAs that were hypomethylated and had higher transcript levels in STEMI govern several pathways related to chromatin activity as well as pathways with more direct relevance for STEMI, such as hemopoiesis, positive regulation of cell migration, and regulation of lipid metabolic process.

In our data, most sites with different methylation patterns between patients with STEMI and healthy controls were found in the 3’UTR and in the CDS. This is not surprising, as in humans, m^6^A sites are enriched in coding sequences and in 3’UTR, especially near stop codons (51, 52). This shift in m^6^A methylation could also contribute to the responses at the translational level, that is not reflected by the mRNA level in the sample.

MI is associated with an inflammatory response. Opening the infarct-related artery can cause ischemia-reperfusion injury and further increase inflammation. Our data show that patients with STEMI have a massive hypermethylation of m^6^A sites 3–7 days after MI compared with the m^6^A methylation pattern at hospital admission. Although this shift in m^6^A methylation pattern was seen in whole blood samples obtain 3–7 days after hospital admission, it is possible PCI-induced ischemia reperfusion injury at least partly could have contributed to this pattern. m^6^A regulation rapidly alters the stability, function or activity of the mRNA transcript. Thus, the m^6^A pattern might mirror the rapid and changing immune responses that occur in these patients after an acute MI. Our findings illustrate the ability of cells to rapidly shift their m^6^A methylation pattern in response to acute events. Yang et al. recently showed that m^6^A regulators were correlated with immune responses, suggesting that immune dysregulation in STEMI was regulated by m^6^A methylation (12).

Previously, we have reported that tocilizumab had beneficial effects on myocardial salvage in STEMI (5). Although there was some overlap with the placebo group, the current study showed differences in the RNA m^6^A methylation pattern between whole blood from patients treated with tocilizumab and those receiving placebo. Our previous report on transcriptome analyses of neutrophils in the ASSAIL-MI trial (26), find that neutrophil degranulation is dampend by tocilizumab. In the present study we found that the m^6^A methylation between the two treatment arms is mainly hypomethylated in the Tocilizumab compared to the placebo arm. Further, annotation analysis of these differently methylated transcripts show involvement in the neutrophil degranulation process such as regulation of extracellular matrix remodeling, neutrophil activation including TLR2 activation and NETs formation. We could speculate that the hypomethylation (i.e., activation) of these transcripts could be a regulatory mechanism to dampen neutrophil degranulation in these patients.

Our study has several important limitations. In particular, the small study population, including both patients and in particular controls, and the heterogeneity within the cohort are important limitations of the present study. This study is hypothesis generating sub-study of the ASSAIL-MI trial examining the role of m^6^A methylation in STEMI and cannot provide a complete picture of the molecular mechanisms involved. Several of the findings such as the enzyme data need to be confirmed at the protein level. Moreover, associations do not necessarily mean any causal relationship. Future studies should comprise a larger number of patients and should also include studies in animal models as well as *in vitro* and *ex vivo* expeients to improve our understanding of the molecular mechanisms governing epitranscriptomics in MI and related atherosclerotic disorders.

## Conclusion

In this hypothesis generating study, we show that *in vivo* m^6^A methylation patterns differ between patients with acute STEMI and healthy individuals. The m^6^A pattern changed after 3 to 7 days. This response was in some degree modulated by IL-6 receptor inhibition. Our data suggest that m^6^A modifications play a role in the inflammatory response after STEMI, potentially representing a novel target for therapy in patients with MI.

## Data Availability

The data will be available for other researchers, but owing to ethical laws in Norway, de-identified data will only be fully available upon reasonable request to the corresponding author and not a part of this publication.
