# Supplementary material for: The N-6 methyladenosine dynamics in STEMI and the effect of IL-6 inhibition - a hypothesis generating sub-study of the ASSAIL-MI trial
Source: Front Immunol. 2025 Jun 6;16:1532325. doi: 10.3389/fimmu.2025.1532325 (PMC12178860; doi:10.3389/fimmu.2025.1532325)
Supplement: Supplementary file 1 [file DataSheet1.pdf]

Supplemental table S1. Differently methylated transcripts STEMI vs HC

Hypermethylated m6A sites							
TransID	*m6A transcript location*	*Gene Symbol*	*Trans biotype*	*Fold change*	*P-value (unpaired t-test)*	*Average STEMI*	*Average HC*
NM_001017963	829	HSP90AA1	protein_coding	3.47	0.018	-6.05	-7.85
NM_001134484	529	TOMM5	protein_coding	3.11	0.028	-6.32	-7.95
NM_005082	5556	TRIM25	protein_coding	2.97	0.048	-4.25	-5.82
NM_005541	4410	INPP5D	protein_coding	2.70	0.019	-3.91	-5.34
NM_018257	1080	PCMTD2	protein_coding	2.62	0.034	-6.20	-7.60
NM_015443_2	1357	KANSL1	protein_coding	2.58	0.003	-5.98	-7.35
NM_005245	3201	FAT1	protein_coding	2.57	0.038	-5.47	-6.83
NR_131012	1610	NEAT1	ncRNA	2.54	0.033	-2.76	-4.11
NM_012482	770	ZNF281	protein_coding	2.43	0.025	-4.78	-6.06
NM_015960	452	CUTC	protein_coding	2.42	0.005	-6.35	-7.63
NM_002811	1116	PSMD7	protein_coding	2.36	0.026	-6.37	-7.61
NM_006407	547	ARL6IP5	protein_coding	2.35	0.005	-6.21	-7.44
NM_002296	3656	LBR	protein_coding	2.35	0.033	-4.09	-5.32
NM_005207	4426	CRKL	protein_coding	2.29	0.009	-6.55	-7.75
NM_006825	1756	CKAP4	protein_coding	2.26	0.036	-5.63	-6.81
NM_006825	1798	CKAP4	protein_coding	2.19	0.038	-5.19	-6.32
NM_012073	1369	CCT5	protein_coding	2.17	0.022	-6.25	-7.37
NM_052947	5376	ALPK2	protein_coding	2.09	0.033	-5.83	-6.90
NM_145055	986	C18orf25	protein_coding	2.09	0.047	-6.29	-7.35
NM_001277817	1575	YTHDF3	protein_coding	2.07	0.029	-5.55	-6.60
NM_001026	592	RPS24	protein_coding	2.07	0.036	-6.58	-7.62
NM_024607	2317	PPP1R3B	protein_coding	1.92	0.027	-5.13	-6.07
NM_032479	582	MRPL36	protein_coding	1.86	0.043	-6.12	-7.01
NM_006555	691	YKT6	protein_coding	1.85	0.015	-5.77	-6.66
NM_001017963	1337	HSP90AA1	protein_coding	1.85	0.035	-6.10	-6.98
NM_018717	4811	MAML3	protein_coding	1.85	0.027	-5.85	-6.73
NM_001258393	2002	CLPB	protein_coding	1.81	0.017	-6.33	-7.18
NM_012414	4956	RAB3GAP2	protein_coding	1.75	0.032	-6.43	-7.23
NM_017926	705	GPATCH2L	protein_coding	1.74	0.037	-5.77	-6.57
NM_016205	2743	PDGFC	protein_coding	1.72	0.014	-5.07	-5.85
NM_020755	1387	SERINC1	protein_coding	1.71	0.023	-6.52	-7.29
NM_004941	4061	DHX8	protein_coding	1.66	0.028	-6.43	-7.16
NR_001564	17935	XIST	ncRNA	1.63	0.029	-6.16	-6.86
NM_014159	4140	SETD2	protein_coding	1.60	0.045	-6.01	-6.69
NM_012336	1523	NARF	protein_coding	1.59	0.030	-4.88	-5.55
NM_000508	1230	FGA	protein_coding	1.58	0.027	-5.98	-6.64
**Hypomethylated m6A sites**							
TransID	*m6A transcript location*	*Gene Symbol*	*Trans biotype*	*Fold change*	*P-value (unpaired t-test)*	*Average STEMI*	*Average HC*
NM_020733	4159	HEG1	protein_coding	0.67	0.023	-3.51	-2.93
NM_053052	2226	SNAP47	protein_coding	0.67	0.026	-4.23	-3.64
NM_001080826	3070	PRAG1	protein_coding	0.67	0.008	-4.07	-3.48
NM_001098832	1922	FAM104A	protein_coding	0.67	0.043	-5.50	-4.92
NM_015325	2767	ICE1	protein_coding	0.67	0.033	-3.22	-2.63
NM_024562	3600	TANGO6	protein_coding	0.67	0.009	-4.89	-4.30
NM_015885	2732	PCF11	protein_coding	0.67	0.031	-4.01	-3.43

NM_001039618	1257	CREBZF	protein_coding	0.67	0.013	-1.62	-1.03
NM_000967	266	RPL3	protein_coding	0.67	0.017	-4.12	-3.53
NM_001003699	3331	RREB1	protein_coding	0.67	0.039	-1.53	-0.95
NM_020195	1096	SDR39U1	protein_coding	0.67	0.017	-4.81	-4.22
NM_001122772	3894	AGAP2	protein_coding	0.67	0.015	-3.78	-3.19
NM_016561	1741	BFAR	protein_coding	0.66	0.024	-3.11	-2.52
NM_001360	1994	DHCR7	protein_coding	0.66	0.034	-3.00	-2.41
NM_032795	1428	RPUSD4	protein_coding	0.66	0.050	-5.68	-5.09
NR_003945	6307	GVINP1	ncRNA	0.66	0.024	1.62	2.21
NM_021184	922	C6orf47	protein_coding	0.66	0.046	-1.92	-1.33
NM_005094	2513	SLC27A4	protein_coding	0.66	0.034	-4.23	-3.64
NM_004295	1775	TRAF4	protein_coding	0.66	0.048	3.92	4.52
NM_014409	824	TAF5L	protein_coding	0.66	0.019	-0.38	0.22
NM_180990	765	ZACN	protein_coding	0.66	0.040	-3.80	-3.21
NM_017905	821	TMCO3	protein_coding	0.66	0.019	-0.48	0.12
NM_001242840	863	GUK1	protein_coding	0.66	0.028	2.14	2.73
NM_014634	5102	PPM1F	protein_coding	0.66	0.016	-2.05	-1.45
NM_052916	2811	RNF157	protein_coding	0.66	0.007	-4.20	-3.60
NM_001626	2455	AKT2	protein_coding	0.66	0.020	-2.82	-2.23
NM_001178035	512	CEP85L	protein_coding	0.66	0.032	-2.51	-1.91
NM_152540	711	SCFD2	protein_coding	0.66	0.022	-4.00	-3.40
NM_003906	1016	MCM3AP	protein_coding	0.66	0.017	-2.09	-1.50
NM_030640	1815	DUSP16	protein_coding	0.66	0.007	-3.37	-2.77
NM_001319	2026	CSNK1G2	protein_coding	0.66	0.021	3.36	3.96
NM_016436	3893	PHF20	protein_coding	0.66	0.040	-3.38	-2.79
NM_005245	12984	FAT1	protein_coding	0.66	0.028	-4.33	-3.73
NM_015316	3874	PPP1R13B	protein_coding	0.66	0.039	-3.88	-3.28
NM_007300	6408	BRCA1	protein_coding	0.66	0.008	-3.98	-3.38
NM_015270	3876	ADCY6	protein_coding	0.66	0.009	-0.01	0.59
NM_080652	1000	TMEM41A	protein_coding	0.66	0.039	-1.59	-0.99
NM_001303525	713	TUBB6	protein_coding	0.66	0.021	-3.36	-2.75
NM_020745	3673	AARS2	protein_coding	0.66	0.006	-3.94	-3.34
NM_181782	1782	NCOA7	protein_coding	0.66	0.046	-3.24	-2.64
NM_017819	1319	TRMT10C	protein_coding	0.66	0.029	-5.70	-5.09
NM_001142292	1292	LMAN2L	protein_coding	0.66	0.018	-1.73	-1.13
NM_004793	2936	LONP1	protein_coding	0.66	0.021	-4.59	-3.98
NM_001171741	882	C3orf18	protein_coding	0.66	0.012	-4.02	-3.42
NM_007242	1834	DDX19B	protein_coding	0.66	0.022	-6.07	-5.46
NM_001083600	1359	NAA60	protein_coding	0.66	0.017	-3.01	-2.41
NM_024747	2235	HPS6	protein_coding	0.66	0.050	-2.20	-1.60
NM_032382	1701	COG8	protein_coding	0.66	0.012	1.36	1.96
NR_024606	1977	LBX2-AS1	ncRNA	0.66	0.023	-1.34	-0.74
NM_004818	2877	DDX23	protein_coding	0.66	0.027	-1.98	-1.37
NM_017528	118	BUD23	protein_coding	0.66	0.048	-0.72	-0.11
NM_014801	1039	PCNX2	protein_coding	0.66	0.018	-3.71	-3.10
NM_006598	3701	SLC12A7	protein_coding	0.66	0.024	-2.08	-1.47
NM_001077663	2242	URGCP	protein_coding	0.66	0.043	-3.00	-2.40
NM_001008895	2432	CUL4A	protein_coding	0.66	0.038	-2.32	-1.72
NM_000757	2583	CSF1	protein_coding	0.66	0.023	-3.15	-2.55
NM_014938	3918	MLXIP	protein_coding	0.66	0.009	-2.45	-1.84
NM_001429	773	EP300	protein_coding	0.66	0.015	-0.74	-0.14

NM_005777	1217	RBM6	protein_coding	0.66	0.011	-2.96	-2.36
NM_000127	1257	EXT1	protein_coding	0.66	0.017	-2.49	-1.89
NM_032479	265	MRPL36	protein_coding	0.66	0.028	-1.72	-1.11
NM_032714	1151	INF2	protein_coding	0.66	0.044	-4.70	-4.10
NM_018052	2667	VAC14	protein_coding	0.66	0.033	-1.75	-1.15
NM_018047	1523	RBM22	protein_coding	0.66	0.001	-3.69	-3.08
NM_006831	398	CLP1	protein_coding	0.66	0.010	-1.44	-0.83
NM_017703	1605	FBXL12	protein_coding	0.66	0.008	-5.04	-4.43
NM_014760	3557	TATDN2	protein_coding	0.66	0.046	-6.28	-5.67
NM_007317	1107	KIF22	protein_coding	0.66	0.025	-1.64	-1.04
NM_001242854	1331	ARFIP2	protein_coding	0.66	0.013	-4.65	-4.04
NM_014755	2742	SERTAD2	protein_coding	0.66	0.028	-4.95	-4.34
NM_017782	6542	FAM208B	protein_coding	0.66	0.046	-2.56	-1.96
NM_033387	3803	FAM78A	protein_coding	0.66	0.015	-5.03	-4.42
NM_001079539	861	XBP1	protein_coding	0.66	0.011	-3.48	-2.87
NM_002334	6395	LRP4	protein_coding	0.66	0.021	-3.62	-3.01
NM_012420	1479	IFIT5	protein_coding	0.66	0.044	-2.44	-1.83
NM_001163544	3910	SYNRG	protein_coding	0.66	0.044	-3.39	-2.78
NM_020825	1552	CRAMP1	protein_coding	0.66	0.030	2.46	3.07
NM_001113407	1450	LDB1	protein_coding	0.66	0.025	-4.19	-3.58
NM_153045	1660	TMEM268	protein_coding	0.65	0.027	-0.49	0.12
NM_020814	4342	MARCH4	protein_coding	0.65	0.007	-2.17	-1.56
NM_003045	2540	SLC7A1	protein_coding	0.65	0.017	4.45	5.06
NM_013276	1696	SHPK	protein_coding	0.65	0.024	-0.49	0.12
NM_007346	1541	OGFR	protein_coding	0.65	0.017	-2.63	-2.02
NM_032287	2545	RTL6	protein_coding	0.65	0.041	-3.54	-2.93
NM_001003940	1452	BMF	protein_coding	0.65	0.034	-5.33	-4.72
NM_022106	845	FAM217B	protein_coding	0.65	0.018	-3.60	-2.99
NM_020901	3968	PHRF1	protein_coding	0.65	0.026	-5.26	-4.64
NM_004556	1958	NFKBIE	protein_coding	0.65	0.007	-3.74	-3.13
NR_015431	517	LINC-PINT	ncRNA	0.65	0.024	-2.72	-2.11
NR_103464	940	HSPA14	ncRNA	0.65	0.038	-5.85	-5.24
NM_003129	1373	SQLE	protein_coding	0.65	0.013	-5.73	-5.11
NM_020762	3256	SRGAP1	protein_coding	0.65	0.013	-0.25	0.36
NM_014413	3219	EIF2AK1	protein_coding	0.65	0.042	-6.39	-5.77
NM_203403	703	LURAP1L	protein_coding	0.65	0.022	-4.17	-3.55
NM_001185011	1165	NCAPH2	protein_coding	0.65	0.026	-1.00	-0.39
NM_014613	1693	FAF2	protein_coding	0.65	0.034	-3.17	-2.55
NM_003592	2847	CUL1	protein_coding	0.65	0.024	-3.82	-3.21
NM_001005361	1555	DNM2	protein_coding	0.65	0.010	-3.38	-2.76
NM_001010924	3342	FAM171A1	protein_coding	0.65	0.026	-1.45	-0.84
NM_001908	1314	CTSB	protein_coding	0.65	0.021	-2.92	-2.30
NM_001183	1687	ATP6AP1	protein_coding	0.65	0.005	-1.77	-1.15
NM_001122964	76	PPP4R3B	protein_coding	0.65	0.041	-4.99	-4.37
NM_006088	364	TUBB4B	protein_coding	0.65	0.033	-4.18	-3.56
NM_006379	2789	SEMA3C	protein_coding	0.65	0.047	-3.25	-2.63
NM_004824	702	CDYL	protein_coding	0.65	0.018	1.34	1.96
NM_030765	2325	B3GNT4	protein_coding	0.65	0.013	-2.44	-1.82
NM_015172	8582	PRRC2C	protein_coding	0.65	0.019	-5.17	-4.55
NM_001257098	603	RHNO1	protein_coding	0.65	0.011	-2.68	-2.06
NM_015726	641	DCAF8	protein_coding	0.65	0.033	-2.49	-1.87

NM_001025205	107	AP2M1	protein_coding	0.65	0.025	-6.08	-5.46
NM_001256183	3183	ANKRD11	protein_coding	0.65	0.008	-2.23	-1.61
NM_002017	1379	FLI1	protein_coding	0.65	0.034	-1.52	-0.90
NM_001128148	2451	TFRC	protein_coding	0.65	0.039	-5.57	-4.95
NM_004085	487	TIMM8A	protein_coding	0.65	0.025	-2.14	-1.51
NM_003977	1122	AIP	protein_coding	0.65	0.018	2.56	3.18
NM_012111	1144	AHSA1	protein_coding	0.65	0.035	0.54	1.16
NM_017582	1496	UBE2Q1	protein_coding	0.65	0.003	-5.85	-5.22
NM_021181	2625	SLAMF7	protein_coding	0.65	0.009	-3.26	-2.63
NM_017612	1946	ZCCHC8	protein_coding	0.65	0.033	-0.88	-0.25
NM_203374	1696	ZNF784	protein_coding	0.65	0.017	-1.98	-1.36
NM_001270507	2144	TNFAIP3	protein_coding	0.65	0.009	-2.65	-2.03
NM_001714	1221	BICD1	protein_coding	0.65	0.039	-0.07	0.55
NM_005113	523	GOLGA5	protein_coding	0.65	0.036	-5.76	-5.14
NM_023012	714	RSRC2	protein_coding	0.65	0.013	-1.93	-1.30
NM_005669	494	REEP5	protein_coding	0.65	0.037	-0.40	0.22
NM_030665	5123	RAI1	protein_coding	0.65	0.037	-1.36	-0.73
NM_001005415	899	MARCH2	protein_coding	0.65	0.011	-2.93	-2.30
NM_022903	1041	CCDC71	protein_coding	0.65	0.011	-4.09	-3.46
NM_001042646	3059	TRAK1	protein_coding	0.65	0.018	-1.82	-1.20
NM_001258311	1788	PGBD5	protein_coding	0.65	0.017	0.93	1.56
NM_015666	903	MTG2	protein_coding	0.65	0.028	-3.34	-2.72
NM_001242783_6	1277	TRIM26	protein_coding	0.65	0.032	-3.94	-3.31
NM_018890	1300	RAC1	protein_coding	0.65	0.035	-0.97	-0.34
NM_005811	2921	GDF11	protein_coding	0.65	0.024	-4.55	-3.92
NM_004104	5484	FASN	protein_coding	0.65	0.015	-1.70	-1.07
NM_033396	409	TNKS1BP1	protein_coding	0.65	0.047	-4.45	-3.82
NM_000958	1594	PTGER4	protein_coding	0.65	0.023	-4.88	-4.25
NM_017617	6836	NOTCH1	protein_coding	0.65	0.045	-2.17	-1.54
NM_020649	876	CBX8	protein_coding	0.65	0.032	-3.63	-3.00
NM_005990	1825	STK10	protein_coding	0.65	0.042	0.49	1.12
NM_014521	1063	SH3BP4	protein_coding	0.65	0.033	-4.06	-3.43
NM_001253699	3465	ERBIN	protein_coding	0.65	0.039	-2.81	-2.18
NM_018452	1282	TMEM242	protein_coding	0.65	0.016	-0.41	0.22
NM_032316	967	NICN1	protein_coding	0.65	0.029	-2.05	-1.42
NM_018051	310	WDR60	protein_coding	0.65	0.044	-5.76	-5.13
NM_021241	1951	WIZ	protein_coding	0.65	0.019	-5.58	-4.95
NM_002649	1835	PIK3CG	protein_coding	0.65	0.029	-1.69	-1.06
NM_001271441	2408	CFAP410	protein_coding	0.65	0.019	-0.48	0.15
NM_001030006	1207	AP2B1	protein_coding	0.64	0.036	-6.45	-5.82
NM_001143810	985	BDNF	protein_coding	0.64	0.014	-5.09	-4.46
NM_017772	1941	TBC1D22B	protein_coding	0.64	0.009	-1.68	-1.05
NM_005777	768	RBM6	protein_coding	0.64	0.044	-5.83	-5.19
NM_003488	919	AKAP1	protein_coding	0.64	0.017	-4.02	-3.38
NM_001218	1304	CA12	protein_coding	0.64	0.044	-3.22	-2.58
NM_016507	297	CDK12	protein_coding	0.64	0.050	-0.31	0.33
NR_045128	1482	TXLNGY	ncRNA	0.64	0.014	-1.85	-1.21
NM_001110556	4750	FLNA	protein_coding	0.64	0.049	-5.76	-5.12
NM_001478	2401	B4GALNT1	protein_coding	0.64	0.023	-3.60	-2.96
NM_173551	6929	ANKS6	protein_coding	0.64	0.038	-5.21	-4.57
NM_032427	2060	MAML2	protein_coding	0.64	0.046	-6.08	-5.44

NM_004417	1294	DUSP1	protein_coding	0.64	0.038	5.30	5.94
NM_005137	2122	DGCR2	protein_coding	0.64	0.034	-0.25	0.39
NM_177542	142	SNRPD2	protein_coding	0.64	0.037	-2.36	-1.72
NM_006360	314	EIF3M	protein_coding	0.64	0.050	-5.28	-4.64
NM_014825	3358	URB1	protein_coding	0.64	0.026	-2.79	-2.15
NM_016604	1873	KDM3B	protein_coding	0.64	0.035	-6.03	-5.38
NM_020933	3278	ZNF317	protein_coding	0.64	0.020	-0.96	-0.32
NM_033290	5395	MID1	protein_coding	0.64	0.031	-5.64	-5.00
NM_001819	1038	CHGB	protein_coding	0.64	0.032	-2.95	-2.31
NM_005148	1060	UNC119	protein_coding	0.64	0.033	-6.21	-5.56
NM_016034	549	MRPS2	protein_coding	0.64	0.017	-4.75	-4.11
NM_015112	5395	MAST2	protein_coding	0.64	0.015	-0.67	-0.03
NM_144775	3336	SMCR8	protein_coding	0.64	0.018	-6.40	-5.75
NM_004996	4972	ABCC1	protein_coding	0.64	0.023	-2.18	-1.54
NM_006923	1314	SDF2	protein_coding	0.64	0.023	3.05	3.70
NM_003489	3408	NRIP1	protein_coding	0.64	0.009	-2.08	-1.44
NM_153182	1481	RIOX2	protein_coding	0.64	0.011	-3.30	-2.66
NM_183047	4418	ZMYND8	protein_coding	0.64	0.019	-3.63	-2.99
NM_001042681	5536	RERE	protein_coding	0.64	0.025	2.01	2.65
NM_144975	2814	SLFN5	protein_coding	0.64	0.015	-4.88	-4.23
NM_018036	6770	ATG2B	protein_coding	0.64	0.017	-4.76	-4.11
NM_001080414	1928	CCDC88C	protein_coding	0.64	0.033	-3.92	-3.27
NM_024496	939	IRF2BPL	protein_coding	0.64	0.026	-2.21	-1.57
NM_001243439	752	SPECC1	protein_coding	0.64	0.036	0.05	0.70
NM_005236	2122	ERCC4	protein_coding	0.64	0.035	-2.93	-2.29
NM_001025091	2661	ABCF1	protein_coding	0.64	0.035	3.00	3.65
NM_001261834	2143	TBRG4	protein_coding	0.64	0.045	-6.37	-5.73
NM_020732	4429	ARID1B	protein_coding	0.64	0.033	-3.96	-3.32
NM_006460	2787	HEXIM1	protein_coding	0.64	0.024	-3.74	-3.10
NM_001145928	3328	SAP130	protein_coding	0.64	0.024	-6.49	-5.84
NM_018358	2235	ABCF3	protein_coding	0.64	0.043	1.52	2.17
NM_053043	3240	RBM33	protein_coding	0.64	0.015	-5.43	-4.78
NM_080861	1298	SPSB3	protein_coding	0.64	0.011	-2.31	-1.66
NM_001145299	2322	EXOC7	protein_coding	0.64	0.011	-1.78	-1.13
NM_002886	927	RAP2B	protein_coding	0.64	0.032	-1.18	-0.53
NM_172206	2755	CAMKK1	protein_coding	0.64	0.027	-5.34	-4.69
NM_012204	2021	GTF3C4	protein_coding	0.64	0.002	-5.91	-5.26
NM_018461	1496	PPP2R2D	protein_coding	0.64	0.046	-2.53	-1.88
NM_001002295	2127	GATA3	protein_coding	0.64	0.020	-3.57	-2.92
NM_181489	3771	ZNF445	protein_coding	0.64	0.017	-4.65	-4.00
NM_003749	5104	IRS2	protein_coding	0.64	0.042	-3.41	-2.76
NM_033390	2296	ZC3H12C	protein_coding	0.64	0.019	-4.16	-3.51
NM_001267782	1355	AMBRA1	protein_coding	0.64	0.013	-4.88	-4.23
NR_152210	2567	AHSA2P	ncRNA	0.64	0.021	-5.20	-4.54
NM_001184740	1777	CTTN	protein_coding	0.64	0.018	-1.57	-0.92
NM_001136262	1113	ATXN7L3B	protein_coding	0.64	0.030	-5.27	-4.62
NM_033224	1205	PURB	protein_coding	0.64	0.031	0.03	0.68
NM_012401	799	PLXNB2	protein_coding	0.64	0.034	-1.51	-0.86
NM_001134338	771	RNF24	protein_coding	0.64	0.030	-1.08	-0.42
NM_020772	853	NUFIP2	protein_coding	0.64	0.027	-3.56	-2.91
NM_014071	5872	NCOA6	protein_coding	0.63	0.012	-5.17	-4.52

NM_007279	444	U2AF2	protein_coding	0.63	0.010	-2.28	-1.62
NM_001270975	1399	IST1	protein_coding	0.63	0.033	-3.03	-2.37
NM_014643	1450	ZNF516	protein_coding	0.63	0.018	-1.50	-0.84
NM_020899	5409	ZBTB4	protein_coding	0.63	0.023	-5.04	-4.38
NM_001012241	1101	MSL1	protein_coding	0.63	0.011	-2.28	-1.62
NM_013437	851	LRP12	protein_coding	0.63	0.042	4.05	4.70
NM_005216	780	DDOST	protein_coding	0.63	0.045	-1.19	-0.53
NM_020461	4122	TUBGCP6	protein_coding	0.63	0.019	-4.59	-3.93
NM_170725	1819	PGBD2	protein_coding	0.63	0.017	-4.74	-4.08
NM_001012279	3314	SOGA3	protein_coding	0.63	0.003	-3.87	-3.21
NM_005647	4535	TBL1X	protein_coding	0.63	0.029	-0.23	0.43
NM_001661	452	ARL4D	protein_coding	0.63	0.036	-5.02	-4.36
NM_017929	1319	PEX26	protein_coding	0.63	0.010	-5.53	-4.87
NM_024121	913	TMEM185B	protein_coding	0.63	0.025	-3.01	-2.35
NM_148920	2870	PIGQ	protein_coding	0.63	0.037	-3.73	-3.07
NM_024097	560	C1orf50	protein_coding	0.63	0.043	-6.37	-5.70
NM_001080477	6849	TENM3	protein_coding	0.63	0.009	-5.72	-5.06
NM_000852	875	GSTP1	protein_coding	0.63	0.031	-0.95	-0.28
NM_001042681	4805	RERE	protein_coding	0.63	0.048	-0.78	-0.12
NM_013336	2137	SEC61A1	protein_coding	0.63	0.039	-2.29	-1.62
NM_001134439	1021	PHLDB2	protein_coding	0.63	0.047	-4.77	-4.11
NM_014805	1645	EPM2AIP1	protein_coding	0.63	0.039	-0.50	0.16
NM_001194998	5163	CEP152	protein_coding	0.63	0.014	-3.38	-2.72
NM_001242739	1225	ZNF691	protein_coding	0.63	0.047	-4.89	-4.23
NM_014777	3663	URB2	protein_coding	0.63	0.024	-2.73	-2.07
NM_182557	2211	BCL9L	protein_coding	0.63	0.044	-4.90	-4.23
NM_005050	2157	ABCD4	protein_coding	0.63	0.026	-0.08	0.59
NM_144775	2804	SMCR8	protein_coding	0.63	0.042	1.50	2.17
NM_001252119	2490	PASK	protein_coding	0.63	0.014	-0.44	0.22
NM_032182	1297	ABRAXAS2	protein_coding	0.63	0.049	0.07	0.73
NM_145265	1057	CCDC127	protein_coding	0.63	0.036	0.07	0.74
NM_015695	1164	BRPF3	protein_coding	0.63	0.024	-2.37	-1.70
NM_018984	3700	SSH1	protein_coding	0.63	0.007	-1.37	-0.71
NR_026915	643	AADACP1	ncRNA	0.63	0.025	-0.72	-0.05
NM_014727	1040	KMT2B	protein_coding	0.63	0.029	-0.71	-0.04
NM_014870	6742	ZBTB40	protein_coding	0.63	0.003	-1.84	-1.17
NM_006401	992	ANP32B	protein_coding	0.63	0.009	-3.71	-3.05
NM_001810	1051	CENPB	protein_coding	0.63	0.040	-5.25	-4.58
NM_015210	4594	MTCL1	protein_coding	0.63	0.021	-3.90	-3.23
NM_001195752	921	THAP3	protein_coding	0.63	0.031	-3.78	-3.11
NM_001163809	2077	WDR81	protein_coding	0.63	0.020	-3.40	-2.73
NM_015282	7105	CLASP1	protein_coding	0.63	0.028	-3.16	-2.49
NM_148920	1808	PIGQ	protein_coding	0.63	0.018	-1.99	-1.32
NM_002254	4789	KIF3C	protein_coding	0.63	0.032	4.91	5.58
NM_152914	436	NATD1	protein_coding	0.63	0.010	-3.25	-2.58
NM_005631	2609	SMO	protein_coding	0.63	0.036	0.81	1.48
NM_002957	2056	RXRA	protein_coding	0.63	0.042	-4.27	-3.60
NM_006977	867	ZBTB25	protein_coding	0.63	0.047	3.66	4.33
NM_003302	1668	TRIP6	protein_coding	0.63	0.039	2.44	3.11
NM_033418	497	METTL18	protein_coding	0.63	0.006	-3.49	-2.82
NM_005370	640	RAB8A	protein_coding	0.63	0.004	-4.21	-3.54

NM_012121	424	CDC42EP4	protein_coding	0.63	0.015	-0.78	-0.10
NM_006390	3409	IPO8	protein_coding	0.63	0.030	-1.79	-1.11
NM_001408	2744	CELSR2	protein_coding	0.63	0.033	-3.99	-3.32
NM_017798	563	YTHDF1	protein_coding	0.63	0.019	-0.45	0.23
NM_014825	3536	URB1	protein_coding	0.63	0.009	-3.77	-3.10
NM_014602	1090	PIK3R4	protein_coding	0.63	0.006	-1.47	-0.80
NM_003512	485	HIST1H2AC	protein_coding	0.63	0.009	-4.08	-3.40
NM_014286	1005	NCS1	protein_coding	0.63	0.010	-5.09	-4.41
NM_032444	7043	SLX4	protein_coding	0.63	0.014	0.10	0.77
NM_016332	901	MSRB1	protein_coding	0.63	0.045	1.36	2.04
NM_004462	1296	FDFT1	protein_coding	0.63	0.032	-0.21	0.47
NM_020461	4041	TUBGCP6	protein_coding	0.63	0.037	-4.68	-4.00
NM_015527	2404	TBC1D10B	protein_coding	0.63	0.038	-5.03	-4.36
NM_001244249	1078	APEX1	protein_coding	0.63	0.029	-3.02	-2.35
NM_198925	3466	SEMA4B	protein_coding	0.63	0.033	-2.02	-1.34
NM_014911	12955	AAK1	protein_coding	0.63	0.014	0.15	0.83
NM_001240	1938	CCNT1	protein_coding	0.63	0.049	-2.99	-2.31
NM_182557	5055	BCL9L	protein_coding	0.63	0.026	0.03	0.71
NM_001112734	998	ZSCAN30	protein_coding	0.62	0.038	-3.79	-3.11
NM_003036	2738	SKI	protein_coding	0.62	0.008	-2.34	-1.66
NM_183047	2495	ZMYND8	protein_coding	0.62	0.023	2.39	3.07
NM_007186	6967	CEP250	protein_coding	0.62	0.008	-5.56	-4.88
NM_001037984	3330	SLC38A10	protein_coding	0.62	0.003	-4.81	-4.13
NM_003119	2834	SPG7	protein_coding	0.62	0.029	-1.06	-0.38
NM_183415	4892	UBE3B	protein_coding	0.62	0.019	-5.57	-4.89
NM_001201552	1634	ZNF821	protein_coding	0.62	0.021	-4.47	-3.79
NM_014011	697	SOCS5	protein_coding	0.62	0.002	-4.25	-3.57
NM_025010	1727	KLHL18	protein_coding	0.62	0.037	-4.76	-4.08
NM_001080414	2119	CCDC88C	protein_coding	0.62	0.028	-4.10	-3.42
NM_198488	3393	FAM83H	protein_coding	0.62	0.013	-1.36	-0.68
NM_003257	3618	TJP1	protein_coding	0.62	0.037	-0.12	0.56
NM_025137	5563	SPG11	protein_coding	0.62	0.027	-1.86	-1.18
NM_152362	802	TNFAIP8L1	protein_coding	0.62	0.013	1.35	2.03
NM_006423	82	RABAC1	protein_coding	0.62	0.010	0.55	1.23
NM_016333	2061	SRRM2	protein_coding	0.62	0.027	-1.56	-0.88
NM_152617	1645	RNF168	protein_coding	0.62	0.032	-6.48	-5.79
NM_021962	2798	ABR	protein_coding	0.62	0.045	-2.72	-2.03
NM_001261826	4997	AP3D1	protein_coding	0.62	0.014	-6.16	-5.48
NM_016388	690	TRAT1	protein_coding	0.62	0.020	1.99	2.67
NM_021826	240	FASTKD5	protein_coding	0.62	0.024	1.19	1.88
NM_020440	5720	PTGFRN	protein_coding	0.62	0.042	-5.05	-4.36
NM_018994	2086	FBXO42	protein_coding	0.62	0.031	1.15	1.83
NM_000116	1295	TAZ	protein_coding	0.62	0.030	-5.07	-4.39
NM_001017963	2490	HSP90AA1	protein_coding	0.62	0.021	-4.27	-3.59
NM_020532	191	RTN4	protein_coding	0.62	0.046	3.62	4.30
NM_030811	671	MRPS26	protein_coding	0.62	0.047	-5.64	-4.95
NM_002105	1279	H2AFX	protein_coding	0.62	0.048	-1.69	-1.00
NM_024536	2049	CHPF	protein_coding	0.62	0.032	-2.52	-1.84
NM_002446	3006	MAP3K10	protein_coding	0.62	0.010	2.27	2.96
NM_006753	1211	SURF6	protein_coding	0.62	0.032	4.84	5.53
NM_006139	507	CD28	protein_coding	0.62	0.035	-5.61	-4.92

NM_032689	4026	ZNF607	protein_coding	0.62	0.024	-4.96	-4.27
NM_000527	3467	LDLR	protein_coding	0.62	0.026	-2.69	-2.00
NM_001123329	1716	ZBTB1	protein_coding	0.62	0.004	-3.60	-2.91
NR_003945	2603	GVINP1	ncRNA	0.62	0.006	-4.03	-3.34
NM_015049	2873	TRAK2	protein_coding	0.62	0.025	-3.85	-3.16
NM_012145	1024	DTYMK	protein_coding	0.62	0.043	-2.59	-1.89
NM_015342	739	PPWD1	protein_coding	0.62	0.007	-3.99	-3.29
NM_001039770	1590	TMPPE	protein_coding	0.62	0.037	-5.35	-4.66
NM_018984	3472	SSH1	protein_coding	0.62	0.033	-1.74	-1.05
NM_183400	759	RNF14	protein_coding	0.62	0.012	-0.64	0.05
NM_001042555	1407	FRS2	protein_coding	0.62	0.043	-5.13	-4.44
NM_005762	2693	TRIM28	protein_coding	0.62	0.006	-4.83	-4.14
NM_001014839	509	NCDN	protein_coding	0.62	0.016	1.24	1.94
NM_022051	3812	EGLN1	protein_coding	0.62	0.037	-6.53	-5.84
NM_014804	757	KIAA0753	protein_coding	0.62	0.026	-3.71	-3.02
NM_080867	711	SOCS4	protein_coding	0.62	0.001	-4.45	-3.76
NM_020198	1885	CCDC47	protein_coding	0.62	0.015	-2.00	-1.31
NM_015062	4011	PPRC1	protein_coding	0.62	0.029	-2.60	-1.91
NM_018143_2	1596	KLHL11	protein_coding	0.62	0.024	-1.78	-1.08
NM_006788	3172	RALBP1	protein_coding	0.62	0.026	-1.77	-1.06
NM_004690	1495	LATS1	protein_coding	0.62	0.031	0.24	0.94
NM_014976	3005	PDCD11	protein_coding	0.62	0.040	3.73	4.43
NM_001143887	1611	COPS2	protein_coding	0.62	0.029	-2.54	-1.84
NR_040662_4	1264	HCP5	ncRNA	0.62	0.014	-1.47	-0.77
NM_020857	722	VPS18	protein_coding	0.62	0.042	-6.15	-5.45
NM_005926	1534	MFAP1	protein_coding	0.61	0.010	-4.07	-3.37
NM_024033	1374	CYREN	protein_coding	0.61	0.045	2.18	2.88
NM_015338	3158	ASXL1	protein_coding	0.61	0.021	-5.25	-4.54
NM_003680	2963	YARS	protein_coding	0.61	0.034	-3.03	-2.33
NM_004747	2870	DLG5	protein_coding	0.61	0.047	-3.10	-2.39
NM_001278074	5988	COL5A1	protein_coding	0.61	0.029	-0.86	-0.15
NM_025054	2301	VCPIP1	protein_coding	0.61	0.042	-3.46	-2.75
NM_002419	65	MAP3K11	protein_coding	0.61	0.033	-6.27	-5.57
NM_020786	1319	PDP2	protein_coding	0.61	0.033	-5.73	-5.02
NM_145305	1308	SLC25A43	protein_coding	0.61	0.032	-0.39	0.32
NM_013336	2427	SEC61A1	protein_coding	0.61	0.043	-0.70	0.00
NM_023074	757	ZNF649	protein_coding	0.61	0.027	-2.24	-1.53
NM_014369	1763	PTPN18	protein_coding	0.61	0.022	-2.40	-1.69
NM_022748	4798	TNS3	protein_coding	0.61	0.029	0.08	0.78
NM_001365341	110	SHLD3	protein_coding	0.61	0.028	0.43	1.14
NM_004448	4455	ERBB2	protein_coding	0.61	0.039	-1.12	-0.41
NM_003405	671	YWHAH	protein_coding	0.61	0.009	-6.32	-5.62
NR_132117	313	DNAJC3-DT	ncRNA	0.61	0.045	0.76	1.47
NR_046285	795	LOC100130744	ncRNA	0.61	0.046	-5.85	-5.14
NR_028514	333	PITPNA-AS1	ncRNA	0.61	0.030	-2.45	-1.75
NM_005953	27	MT2A	protein_coding	0.61	0.033	-0.92	-0.21
NM_003633	1833	ENC1	protein_coding	0.61	0.024	-1.42	-0.71
NM_015721	957	GEMIN4	protein_coding	0.61	0.038	-1.96	-1.25
NM_001099638	3115	ZNF146	protein_coding	0.61	0.023	-6.54	-5.83
NM_021127	318	PMAIP1	protein_coding	0.61	0.020	-6.49	-5.78
NM_152293	1343	TADA2B	protein_coding	0.61	0.041	1.59	2.30

NM_182663	1480	RASSF5	protein_coding	0.61	0.025	-3.68	-2.97
NM_032775	883	KLHL22	protein_coding	0.61	0.015	-2.50	-1.79
NM_021975	1755	RELA	protein_coding	0.61	0.036	-3.19	-2.48
NM_001761	2019	CCNF	protein_coding	0.61	0.039	-6.06	-5.35
NM_032970	1080	SEC22C	protein_coding	0.61	0.008	-3.87	-3.15
NM_016507	938	CDK12	protein_coding	0.61	0.011	-4.60	-3.89
NM_178040	4092	ERC1	protein_coding	0.61	0.026	0.27	0.98
NM_004628	1260	XPC	protein_coding	0.61	0.028	-6.62	-5.91
NM_022757	1758	CCDC14	protein_coding	0.61	0.020	-0.74	-0.02
NM_020338	5002	ZMIZ1	protein_coding	0.61	0.024	-1.74	-1.03
NM_020753	3549	CASKIN2	protein_coding	0.61	0.016	-3.58	-2.86
NM_018663	667	PXMP2	protein_coding	0.61	0.044	-5.84	-5.12
NM_052943	1585	TENT5B	protein_coding	0.61	0.013	-3.48	-2.76
NM_001270439	646	ARPC5	protein_coding	0.61	0.032	-6.33	-5.62
NM_015322	843	FEM1B	protein_coding	0.61	0.023	0.05	0.77
NM_001620	15745	AHNAK	protein_coding	0.61	0.044	-2.14	-1.42
NM_004687	2653	MTMR4	protein_coding	0.61	0.014	1.58	2.30
NM_016436	3741	PHF20	protein_coding	0.61	0.021	-2.88	-2.17
NM_016930	1477	STX18	protein_coding	0.61	0.001	-0.97	-0.25
NM_000594	1226	TNF	protein_coding	0.61	0.036	-4.01	-3.29
NM_001430	3984	EPAS1	protein_coding	0.61	0.039	-4.33	-3.61
NM_001100164	1149	PHACTR2	protein_coding	0.61	0.042	-6.08	-5.35
NM_007118	10049	TRIO	protein_coding	0.61	0.022	-3.60	-2.88
NM_024513	1074	FYCO1	protein_coding	0.61	0.013	-5.19	-4.46
NM_020861	1687	ZBTB2	protein_coding	0.61	0.036	-0.19	0.54
NM_024776	4364	PEAK1	protein_coding	0.61	0.023	-2.74	-2.02
NM_016316	843	REV1	protein_coding	0.61	0.019	-2.23	-1.51
NM_020865	68	DHX36	protein_coding	0.61	0.046	-5.06	-4.33
NM_001618	2866	PARP1	protein_coding	0.60	0.013	-1.39	-0.66
NM_007313	2414	ABL1	protein_coding	0.60	0.022	-6.12	-5.40
NM_001353345	1581	SETD1B	protein_coding	0.60	0.007	-1.78	-1.06
NM_000127	1502	EXT1	protein_coding	0.60	0.025	-1.45	-0.73
NM_001099784	3421	FBXL19	protein_coding	0.60	0.045	3.08	3.81
NM_006150	1847	PRICKLE3	protein_coding	0.60	0.017	-4.51	-3.78
NM_144604	2998	ZC3H18	protein_coding	0.60	0.028	-3.61	-2.89
NM_178835	4593	ZNF827	protein_coding	0.60	0.039	5.85	6.58
NM_000527	162	LDLR	protein_coding	0.60	0.029	-5.32	-4.59
NM_001242851	386	RNF146	protein_coding	0.60	0.002	-6.56	-5.83
NM_152892	1958	LRWD1	protein_coding	0.60	0.041	-4.92	-4.19
NM_006977	1054	ZBTB25	protein_coding	0.60	0.048	-2.85	-2.12
NM_020466	1065	LYRM2	protein_coding	0.60	0.035	-5.03	-4.30
NM_032859	817	ABHD13	protein_coding	0.60	0.042	-5.92	-5.19
NM_003435	30	ZNF134	protein_coding	0.60	0.035	-2.00	-1.27
NM_020922	3655	WNK3	protein_coding	0.60	0.013	-0.50	0.24
NM_004284	2594	CHD1L	protein_coding	0.60	0.025	-3.41	-2.67
NM_002618	41	PEX13	protein_coding	0.60	0.015	-5.10	-4.37
NM_030952	1693	NUAK2	protein_coding	0.60	0.023	2.56	3.30
NM_201443	1668	TEAD4	protein_coding	0.60	0.017	-5.66	-4.92
NM_017588	1281	WDR5	protein_coding	0.60	0.025	-2.95	-2.22
NM_001195055	771	DDIT3	protein_coding	0.60	0.049	-2.35	-1.62
NM_000516	521	GNAS	protein_coding	0.60	0.041	-4.81	-4.07

NM_014779	1624	TSC22D2	protein_coding	0.60	0.043	-5.43	-4.70
NM_017436	1735	A4GALT	protein_coding	0.60	0.026	-5.38	-4.65
NM_012398	2951	PIP5K1C	protein_coding	0.60	0.015	-3.64	-2.90
NM_014071	2695	NCOA6	protein_coding	0.60	0.016	-3.34	-2.60
NM_003246	4329	THBS1	protein_coding	0.60	0.019	-5.13	-4.39
NM_020338	4753	ZMIZ1	protein_coding	0.60	0.040	-2.69	-1.95
NM_138428	430	SMIM12	protein_coding	0.60	0.039	-5.12	-4.38
NM_032120	718	RBM48	protein_coding	0.60	0.045	-4.08	-3.34
NM_018197	1752	ZFP64	protein_coding	0.60	0.008	-2.20	-1.46
NM_001145338_4	1239	ZBTB22	protein_coding	0.60	0.032	-3.83	-3.09
NM_015073	6366	SIPA1L3	protein_coding	0.60	0.015	-6.16	-5.42
NM_014866	3392	SEC16A	protein_coding	0.60	0.002	-2.01	-1.27
NM_001008744	419	TDP1	protein_coding	0.60	0.017	-2.07	-1.33
NM_153812	1514	PHF13	protein_coding	0.60	0.005	-3.54	-2.80
NM_005786	3676	TSHZ1	protein_coding	0.60	0.004	-5.78	-5.04
NM_013318	3912	PRRC2B	protein_coding	0.60	0.035	-2.06	-1.32
NM_032217	6357	ANKRD17	protein_coding	0.60	0.015	-2.12	-1.38
NM_001040438_3	537	C6orf48	protein_coding	0.60	0.025	-0.76	-0.02
NM_001025252	528	TPD52	protein_coding	0.60	0.013	-4.22	-3.48
NM_003128	2533	SPTBN1	protein_coding	0.60	0.019	-4.77	-4.03
NM_015340	3201	LARS2	protein_coding	0.60	0.004	-4.66	-3.92
NM_005647	4582	TBL1X	protein_coding	0.60	0.023	-4.49	-3.74
NM_004508	1682	IDI1	protein_coding	0.60	0.027	-3.32	-2.57
NM_001174108	2783	ZBED6	protein_coding	0.60	0.038	-4.71	-3.97
NM_020795	3047	NLGN2	protein_coding	0.60	0.047	-3.35	-2.60
NM_031486	2557	ZNF484	protein_coding	0.60	0.040	-4.89	-4.14
NM_181672	4091	OGT	protein_coding	0.59	0.033	0.00	0.75
NM_024580	3045	EFL1	protein_coding	0.59	0.031	-3.61	-2.86
NM_012156	3446	EPB41L1	protein_coding	0.59	0.035	-3.21	-2.46
NM_003390	2579	WEE1	protein_coding	0.59	0.035	-2.77	-2.02
NM_020718	2995	USP31	protein_coding	0.59	0.014	-1.75	-0.99
NM_001198838	3480	RBM12	protein_coding	0.59	0.004	-3.83	-3.08
NM_000127	1326	EXT1	protein_coding	0.59	0.003	-2.97	-2.22
NM_003051	1104	SLC16A1	protein_coding	0.59	0.023	-4.24	-3.49
NM_016612	995	SLC25A37	protein_coding	0.59	0.038	0.62	1.37
NM_006598	4008	SLC12A7	protein_coding	0.59	0.015	-3.98	-3.23
NM_020795	1654	NLGN2	protein_coding	0.59	0.015	-2.07	-1.32
NM_024546	1448	RNF219	protein_coding	0.59	0.045	-5.28	-4.52
NM_001244134	1812	MAP3K8	protein_coding	0.59	0.006	-3.51	-2.75
NM_020414	1154	DDX24	protein_coding	0.59	0.036	-5.62	-4.86
NM_203500	2142	KEAP1	protein_coding	0.59	0.036	-3.25	-2.50
NM_032515	1861	BOK	protein_coding	0.59	0.033	-3.19	-2.43
NM_001170794	1607	BACH2	protein_coding	0.59	0.036	-5.50	-4.74
NM_004973	1634	JARID2	protein_coding	0.59	0.030	-4.48	-3.72
NM_005650	3552	TCF20	protein_coding	0.59	0.033	-5.96	-5.21
NM_006540	2409	NCOA2	protein_coding	0.59	0.035	-4.70	-3.94
NM_015963	480	THAP4	protein_coding	0.59	0.029	-3.02	-2.26
NM_001244580	12481	TRRAP	protein_coding	0.59	0.025	-4.75	-3.99
NM_013248	548	NXT1	protein_coding	0.59	0.027	-6.09	-5.33
NM_001166139	1097	LCORL	protein_coding	0.59	0.009	-4.41	-3.65
NM_001100594	2497	SNRK	protein_coding	0.59	0.042	-4.48	-3.72

NM_182919	2563	TICAM1	protein_coding	0.59	0.049	-5.76	-5.00
NM_006095	3858	ATP8A1	protein_coding	0.59	0.015	-4.45	-3.69
NM_004415	6169	DSP	protein_coding	0.59	0.031	-6.49	-5.72
NM_001973	2681	ELK4	protein_coding	0.59	0.035	-2.66	-1.89
NM_022841	2302	RFX7	protein_coding	0.59	0.020	-4.68	-3.92
NR_038955	1785	LINC00963	ncRNA	0.59	0.011	-1.71	-0.94
NM_002184	3162	IL6ST	protein_coding	0.59	0.006	-4.34	-3.58
NM_153371	1355	LNX2	protein_coding	0.59	0.011	-3.10	-2.33
NM_001080779	3750	MYO1C	protein_coding	0.59	0.038	-1.66	-0.90
NM_014913	3285	ADNP2	protein_coding	0.59	0.018	-3.45	-2.68
NM_003417	929	ZNF264	protein_coding	0.59	0.007	-2.08	-1.31
NM_000373	587	UMPS	protein_coding	0.59	0.043	-5.17	-4.40
NM_032752	2162	ZNF496	protein_coding	0.59	0.032	-2.44	-1.67
NM_005791	643	MPHOSPH10	protein_coding	0.59	0.048	-3.50	-2.73
NM_016565	305	COA4	protein_coding	0.59	0.010	-5.75	-4.98
NM_014572	1499	LATS2	protein_coding	0.59	0.021	1.76	2.53
NR_047573	63	TMEM44-AS1	ncRNA	0.59	0.034	-4.26	-3.49
NM_014757	3258	MAML1	protein_coding	0.59	0.008	-5.61	-4.84
NM_006943	2486	SOX12	protein_coding	0.59	0.046	-5.67	-4.89
NM_005813	35	PRKD3	protein_coding	0.59	0.048	-0.82	-0.05
NM_004901	1487	ENTPD4	protein_coding	0.59	0.037	-2.81	-2.03
NM_018899_2	2388	PCDHAC2	protein_coding	0.58	0.031	-5.38	-4.60
NM_001184900	2079	CARD8	protein_coding	0.58	0.016	-3.18	-2.41
NM_021908	176	ST7	protein_coding	0.58	0.040	-4.10	-3.33
NM_014908	662	DOLK	protein_coding	0.58	0.027	4.20	4.98
NM_015020	3966	PHLPP2	protein_coding	0.58	0.028	-2.01	-1.24
NM_001961	1929	EEF2	protein_coding	0.58	0.017	0.50	1.28
NM_000434_4	1395	NEU1	protein_coding	0.58	0.011	-1.61	-0.84
NM_001135597	6080	CCDC88A	protein_coding	0.58	0.028	-5.76	-4.98
NM_003797	145	EED	protein_coding	0.58	0.028	-2.77	-1.99
NM_014947	242	FOXJ3	protein_coding	0.58	0.020	-5.76	-4.99
NM_198557	677	RBM43	protein_coding	0.58	0.008	-2.83	-2.05
NM_144498	2404	OSBPL2	protein_coding	0.58	0.013	-6.10	-5.33
NM_006990	5510	WASF2	protein_coding	0.58	0.021	0.39	1.17
NM_015421	491	TMEM186	protein_coding	0.58	0.017	-4.88	-4.10
NM_001992	2555	F2R	protein_coding	0.58	0.048	-3.78	-3.00
NM_004285	344	H6PD	protein_coding	0.58	0.015	-4.30	-3.52
NM_004774	4596	MED1	protein_coding	0.58	0.024	-1.79	-1.01
NM_001007278	714	TRIM13	protein_coding	0.58	0.036	2.10	2.88
NM_203505	282	G3BP2	protein_coding	0.58	0.046	-3.24	-2.46
NM_012302	4258	ADGRL2	protein_coding	0.58	0.034	-3.32	-2.54
NM_001145343	548	ZNF566	protein_coding	0.58	0.009	-4.15	-3.37
NM_001257975	925	CIZ1	protein_coding	0.58	0.025	-5.97	-5.18
NM_001142640	496	TNRC6C	protein_coding	0.58	0.013	-5.43	-4.65
NM_015534	800	ZZZ3	protein_coding	0.58	0.033	-6.51	-5.72
NM_001242785	1237	HLCS	protein_coding	0.58	0.041	-2.86	-2.08
NM_012448	2501	STAT5B	protein_coding	0.58	0.045	-5.09	-4.30
NM_178835	1997	ZNF827	protein_coding	0.58	0.013	2.21	2.99
NM_014506	1037	TOR1B	protein_coding	0.58	0.026	-3.61	-2.83
NM_001042631	767	SDHAF1	protein_coding	0.58	0.020	-4.67	-3.88
NM_032590	1692	KDM2B	protein_coding	0.58	0.032	-1.57	-0.78

NM_017754	3245	UHRF1BP1	protein_coding	0.58	0.017	-3.43	-2.65
NM_171998	963	RAB39B	protein_coding	0.58	0.046	-0.21	0.58
NM_005801	874	EIF1	protein_coding	0.58	0.029	-3.38	-2.59
NM_001005333	49	MAGED1	protein_coding	0.58	0.007	-4.50	-3.71
NM_014918	960	CHSY1	protein_coding	0.58	0.029	-4.86	-4.07
NM_014883	3674	FAM13A	protein_coding	0.58	0.038	-0.32	0.47
NM_004273	1617	CHST3	protein_coding	0.58	0.023	-5.01	-4.22
NM_001048166	1713	STIL	protein_coding	0.58	0.042	-3.99	-3.20
NM_021922	670	FANCE	protein_coding	0.58	0.006	-3.72	-2.93
NM_004514	3032	FOXK2	protein_coding	0.58	0.041	-2.35	-1.56
NM_022347	333	TOR1AIP2	protein_coding	0.58	0.023	-1.77	-0.98
NM_017712	954	PGPEP1	protein_coding	0.58	0.042	2.61	3.40
NM_001206612	1031	CHTOP	protein_coding	0.58	0.039	-2.86	-2.07
NM_170606	11359	KMT2C	protein_coding	0.58	0.044	-1.37	-0.58
NR_033341	1129	LOC100379224	ncRNA	0.58	0.017	-3.96	-3.16
NM_005245	12156	FAT1	protein_coding	0.58	0.024	-4.98	-4.19
NM_002473	4214	MYH9	protein_coding	0.58	0.033	-4.52	-3.72
NM_001287	3344	CLCN7	protein_coding	0.57	0.024	-2.82	-2.02
NR_046507	3393	PINK1-AS	ncRNA	0.57	0.029	-1.29	-0.49
NM_032725	551	BUD13	protein_coding	0.57	0.039	-1.02	-0.22
NM_004093	1230	EFNB2	protein_coding	0.57	0.019	-3.00	-2.20
NM_001366737	860	GCNT4	protein_coding	0.57	0.044	-3.17	-2.37
NM_018334	2294	LRRN3	protein_coding	0.57	0.042	-3.48	-2.68
NM_001244701	410	ZFP36L1	protein_coding	0.57	0.011	-3.01	-2.21
NM_016598	1595	ZDHHC3	protein_coding	0.57	0.011	-2.76	-1.96
NM_001160305	1504	SETD6	protein_coding	0.57	0.023	-3.32	-2.52
NM_001115016	3770	KANSL3	protein_coding	0.57	0.028	-4.56	-3.75
NM_005063	1283	SCD	protein_coding	0.57	0.011	-1.62	-0.81
NM_002417	9808	MKI67	protein_coding	0.57	0.022	-0.74	0.06
NM_031445	1905	AMMECR1L	protein_coding	0.57	0.024	0.28	1.08
NM_014938	3987	MLXIP	protein_coding	0.57	0.032	-3.01	-2.20
NM_020177	843	FEM1C	protein_coding	0.57	0.026	-2.47	-1.66
NM_030937	1714	CCNL2	protein_coding	0.57	0.028	-4.68	-3.87
NM_015221	1261	DNMBP	protein_coding	0.57	0.010	-6.26	-5.45
NR_002196	1647	H19	ncRNA	0.57	0.015	-3.83	-3.02
NM_001164771	2286	SLC7A2	protein_coding	0.57	0.017	-0.79	0.02
NM_000591	1245	CD14	protein_coding	0.57	0.029	-0.09	0.73
NM_001946	1781	DUSP6	protein_coding	0.57	0.042	-7.09	-6.28
NM_000303	1396	PMM2	protein_coding	0.57	0.033	-4.95	-4.14
NM_012433	89	SF3B1	protein_coding	0.57	0.046	-3.40	-2.58
NM_001257397	162	UBE2V1	protein_coding	0.57	0.024	-5.68	-4.86
NM_199360	178	TPD52L2	protein_coding	0.57	0.038	-1.76	-0.94
NM_014762	3093	DHCR24	protein_coding	0.57	0.043	0.23	1.05
NM_000875	10131	IGF1R	protein_coding	0.57	0.039	-4.21	-3.39
NM_015100	4429	POGZ	protein_coding	0.57	0.013	-4.64	-3.82
NR_131012	933	NEAT1	ncRNA	0.57	0.027	-0.05	0.77
NM_001020658	3182	PUM1	protein_coding	0.57	0.030	4.36	5.18
NM_001029882	3761	AHDC1	protein_coding	0.57	0.024	-2.95	-2.13
NM_018706	3278	DHTKD1	protein_coding	0.57	0.044	2.75	3.57
NM_021035	6839	ZNFX1	protein_coding	0.57	0.026	-4.52	-3.70
NM_014805	923	EPM2AIP1	protein_coding	0.57	0.012	-6.54	-5.72

NM_015073	3412	SIPA1L3	protein_coding	0.57	0.040	-5.70	-4.88
NM_175907	1948	ZADH2	protein_coding	0.57	0.014	-5.86	-5.04
NM_001190470	630	MTRNR2L2	protein_coding	0.57	0.009	-2.78	-1.96
NM_024735	1894	FBXO31	protein_coding	0.57	0.014	-6.32	-5.50
NM_001257975	1293	CIZ1	protein_coding	0.57	0.031	-1.18	-0.36
NM_018948	1610	ERRFI1	protein_coding	0.57	0.015	-3.82	-2.99
NM_000098	1404	CPT2	protein_coding	0.57	0.044	-6.32	-5.50
NM_000179	2834	MSH6	protein_coding	0.57	0.005	-1.27	-0.45
NM_012293	3498	PXDN	protein_coding	0.57	0.013	-4.40	-3.57
NM_005934	4337	MLLT1	protein_coding	0.56	0.039	-4.18	-3.35
NM_015203	2797	RPRD2	protein_coding	0.56	0.028	-5.28	-4.45
NM_181672	2967	OGT	protein_coding	0.56	0.015	-3.31	-2.48
NM_005515	198	MNX1	protein_coding	0.56	0.048	-5.14	-4.32
NM_031866	2264	FZD8	protein_coding	0.56	0.050	-2.37	-1.54
NM_001258358	94	HPCAL1	protein_coding	0.56	0.020	-5.21	-4.38
NM_015629	510	PRPF31	protein_coding	0.56	0.009	-6.67	-5.84
NM_004491	2689	ARHGAP35	protein_coding	0.56	0.021	-4.22	-3.38
NM_001080517	3348	SETD5	protein_coding	0.56	0.027	-3.40	-2.57
NM_024622	404	FASTKD1	protein_coding	0.56	0.020	-5.14	-4.31
NM_015042	1594	ZNF609	protein_coding	0.56	0.006	-3.19	-2.36
NM_152260	177	RPUSD2	protein_coding	0.56	0.038	-1.98	-1.14
NM_015510	1220	DHRS7B	protein_coding	0.56	0.034	-2.08	-1.24
NM_013248	892	NXT1	protein_coding	0.56	0.038	-3.78	-2.95
NM_001141947	2039	CCDC66	protein_coding	0.56	0.034	-1.87	-1.04
NM_005895	1385	GOLGA3	protein_coding	0.56	0.036	-3.72	-2.88
NM_001007278	674	TRIM13	protein_coding	0.56	0.034	1.34	2.18
NM_017780	1852	CHD7	protein_coding	0.56	0.039	-2.21	-1.37
NM_001664	439	RHOA	protein_coding	0.56	0.048	-4.96	-4.12
NM_001821	2201	CHML	protein_coding	0.56	0.042	-4.21	-3.37
NM_005066	1448	SFPQ	protein_coding	0.56	0.025	-2.00	-1.16
NM_145173	1535	DIRAS1	protein_coding	0.56	0.050	2.80	3.65
NM_001173487	2087	NKRF	protein_coding	0.56	0.038	-6.45	-5.60
NM_014844	2162	TECPR2	protein_coding	0.56	0.017	-0.75	0.09
NM_001424	1712	EMP2	protein_coding	0.56	0.035	-2.54	-1.69
NM_014866	3274	SEC16A	protein_coding	0.56	0.012	-5.46	-4.61
NM_001083330	2372	ZNF133	protein_coding	0.56	0.027	-4.31	-3.46
NM_017599	2375	VEZT	protein_coding	0.55	0.013	-0.36	0.49
NM_001012241	1183	MSL1	protein_coding	0.55	0.019	-0.22	0.63
NM_001244950	393	SPOCK2	protein_coding	0.55	0.038	-2.74	-1.89
NM_003804	2816	RIPK1	protein_coding	0.55	0.035	-1.76	-0.91
NM_025222	26	WDR82	protein_coding	0.55	0.040	-3.77	-2.92
NM_205860	2186	NR5A2	protein_coding	0.55	0.047	-5.77	-4.92
NM_001037806	1670	NCKAP5L	protein_coding	0.55	0.038	4.47	5.32
NM_014452	2405	TNFRSF21	protein_coding	0.55	0.026	-1.43	-0.58
NM_019042	546	PUS7	protein_coding	0.55	0.013	-6.33	-5.48
NM_020829	4306	RIC1	protein_coding	0.55	0.036	-5.00	-4.15
NM_138444	480	KCTD12	protein_coding	0.55	0.014	-5.07	-4.21
NM_178014_6	2414	TUBB	protein_coding	0.55	0.024	0.00	0.86
NM_007313	2804	ABL1	protein_coding	0.55	0.021	-6.34	-5.48
NM_006309	2708	LRRFIP2	protein_coding	0.55	0.010	-3.23	-2.37
NR_152871	6791	TUG1	ncRNA	0.55	0.041	-1.10	-0.24

NM_016442	479	ERAP1	protein_coding	0.55	0.034	-3.65	-2.79
NM_152490	1731	B3GALNT2	protein_coding	0.55	0.001	-6.02	-5.16
NM_013386	1730	SLC25A24	protein_coding	0.55	0.024	-3.95	-3.08
NM_001112736	5047	FAM208A	protein_coding	0.55	0.035	-0.95	-0.09
NM_001136262	1289	ATXN7L3B	protein_coding	0.55	0.012	-5.23	-4.37
NM_001077199	2019	SREK1	protein_coding	0.55	0.027	-4.38	-3.51
NM_031444	3587	GUCD1	protein_coding	0.55	0.049	-4.68	-3.81
NM_020337	2294	ANKRD50	protein_coding	0.55	0.002	-5.78	-4.91
NM_032511	1400	FAXC	protein_coding	0.55	0.027	-5.04	-4.17
NM_004719	4668	SCAF11	protein_coding	0.55	0.013	-4.55	-3.68
NM_001166215	1576	S1PR5	protein_coding	0.55	0.039	-3.70	-2.83
NM_000112	1353	SLC26A2	protein_coding	0.55	0.023	-6.28	-5.41
NM_171999	4012	SALL3	protein_coding	0.55	0.050	-5.60	-4.72
NM_001145838	986	MTFR1	protein_coding	0.55	0.019	-5.74	-4.86
NM_001256183	3024	ANKRD11	protein_coding	0.55	0.039	-6.63	-5.76
NM_001126111	1521	OSGIN2	protein_coding	0.54	0.008	-3.26	-2.38
NM_032718	937	MFSD9	protein_coding	0.54	0.007	-4.34	-3.47
NM_014982	1718	PCNX1	protein_coding	0.54	0.012	-2.13	-1.25
NM_022766	2667	CERK	protein_coding	0.54	0.039	-5.42	-4.54
NM_018095	83	KBTBD4	protein_coding	0.54	0.033	-4.16	-3.28
NR_026052	294	CENPBD1P1	ncRNA	0.54	0.013	2.19	3.07
NM_019589	1637	YLPM1	protein_coding	0.54	0.029	-2.26	-1.38
NM_032490	494	GON7	protein_coding	0.54	0.044	-1.43	-0.55
NM_016121	2336	KCTD3	protein_coding	0.54	0.046	-5.47	-4.59
NM_173165	2646	NFATC3	protein_coding	0.54	0.019	-3.83	-2.95
NM_001300	535	KLF6	protein_coding	0.54	0.030	-1.36	-0.47
NM_173811	308	HARBI1	protein_coding	0.54	0.040	-6.12	-5.23
NM_001348	2032	DAPK3	protein_coding	0.54	0.046	-4.16	-3.28
NM_004897	1739	MINPP1	protein_coding	0.54	0.012	-6.44	-5.55
NM_014883	3708	FAM13A	protein_coding	0.54	0.008	-4.98	-4.09
NM_005902	1725	SMAD3	protein_coding	0.54	0.011	-6.60	-5.71
NM_012405	2126	ICMT	protein_coding	0.54	0.040	-5.19	-4.29
NM_014883	4010	FAM13A	protein_coding	0.54	0.023	-4.36	-3.47
NM_032689	925	ZNF607	protein_coding	0.54	0.040	-3.73	-2.83
NM_000623	2142	BDKRB2	protein_coding	0.54	0.041	-3.18	-2.29
NM_017622	1040	BORCS6	protein_coding	0.54	0.008	-5.21	-4.31
NM_015542	971	UPF2	protein_coding	0.54	0.032	-6.35	-5.46
NM_152260	156	RPUSD2	protein_coding	0.54	0.030	-3.67	-2.78
NM_006662	8984	SRCAP	protein_coding	0.54	0.018	-3.32	-2.42
NM_022727	756	TRMT2A	protein_coding	0.54	0.033	-6.35	-5.45
NM_133647	4108	SLC12A6	protein_coding	0.54	0.036	-6.22	-5.32
NM_030919	1713	FAM83D	protein_coding	0.54	0.028	-4.65	-3.76
NM_183422	3837	TSC22D1	protein_coding	0.54	0.025	-5.46	-4.56
NM_018717	1440	MAML3	protein_coding	0.54	0.049	-5.61	-4.71
NM_002712	939	PPP1R7	protein_coding	0.54	0.038	-6.32	-5.41
NM_001001928	1220	PPARA	protein_coding	0.54	0.007	-5.87	-4.97
NM_015353	1597	KCTD2	protein_coding	0.54	0.015	-0.45	0.45
NM_197968	4353	ZMYM2	protein_coding	0.53	0.012	-4.45	-3.55
NM_003112	2340	SP4	protein_coding	0.53	0.020	-2.97	-2.07
NM_001242739	1171	ZNF691	protein_coding	0.53	0.025	-0.32	0.58
NM_006145	1194	DNAJB1	protein_coding	0.53	0.035	-2.11	-1.20

NM_031217	2467	KIF18A	protein_coding	0.53	0.027	-4.93	-4.02
NM_003463	861	PTP4A1	protein_coding	0.53	0.049	-3.32	-2.40
NM_052899	1594	GPRIN1	protein_coding	0.53	0.017	-3.58	-2.67
NM_001080517	3543	SETD5	protein_coding	0.53	0.026	-3.11	-2.19
NM_022717	309	SNRNP35	protein_coding	0.53	0.029	-5.54	-4.62
NM_007135	1883	ZNF79	protein_coding	0.53	0.017	0.04	0.96
NM_015059	8073	TLN2	protein_coding	0.53	0.015	-6.04	-5.12
NM_002766	1771	PRPSAP1	protein_coding	0.53	0.044	-6.95	-6.03
NM_017873	2132	ASB6	protein_coding	0.53	0.045	-2.42	-1.49
NM_006775	3922	QKI	protein_coding	0.53	0.037	-4.14	-3.21
NM_022347	682	TOR1AIP2	protein_coding	0.53	0.016	-6.52	-5.60
NM_144628	3966	TBC1D20	protein_coding	0.53	0.028	-4.14	-3.21
NM_198563	1302	STIMATE	protein_coding	0.52	0.003	-5.11	-4.18
NM_001257181	1559	SLC20A2	protein_coding	0.52	0.037	-3.25	-2.31
NM_152705	396	POLR1D	protein_coding	0.52	0.041	-4.71	-3.78
NM_170606	6273	KMT2C	protein_coding	0.52	0.034	-2.93	-2.00
NM_001144831	1331	PHB2	protein_coding	0.52	0.009	0.38	1.31
NM_004850	4670	ROCK2	protein_coding	0.52	0.050	-4.93	-3.99
NM_032199	3970	ARID5B	protein_coding	0.52	0.013	-6.15	-5.22
NM_001039885	1637	FKRP	protein_coding	0.52	0.037	-2.90	-1.96
NM_001002860	3381	BTBD7	protein_coding	0.52	0.010	1.02	1.96
NM_001142782	4543	MAGI3	protein_coding	0.52	0.014	-1.15	-0.21
NM_001111	360	ADAR	protein_coding	0.52	0.024	1.80	2.75
NM_001102426	3451	TBC1D8	protein_coding	0.52	0.034	4.21	5.15
NM_148898	2628	FOXP2	protein_coding	0.52	0.045	-6.07	-5.12
NM_006923	748	SDF2	protein_coding	0.52	0.042	-4.56	-3.62
NM_020727	229	ZBTB21	protein_coding	0.52	0.018	-2.76	-1.81
NM_019051	538	MRPL50	protein_coding	0.52	0.006	-4.58	-3.63
NM_001278196	484	ZBTB18	protein_coding	0.52	0.015	-3.89	-2.95
NM_014344	2315	FJX1	protein_coding	0.52	0.031	-6.60	-5.65
NM_001098491	2049	ZNF419	protein_coding	0.52	0.048	-3.93	-2.98
NM_001040694	1144	INCENP	protein_coding	0.52	0.037	-3.01	-2.06
NM_001258309	2544	NOP2	protein_coding	0.52	0.041	-5.08	-4.12
NM_021964	558	ZNF148	protein_coding	0.52	0.007	-4.10	-3.14
NM_002094	5490	GSPT1	protein_coding	0.52	0.031	-2.59	-1.64
NM_014661	904	FAM53B	protein_coding	0.52	0.030	-3.10	-2.15
NM_033317	1858	DMKN	protein_coding	0.51	0.006	-0.48	0.48
NM_004996	6059	ABCC1	protein_coding	0.51	0.021	-3.66	-2.70
NM_001134338	667	RNF24	protein_coding	0.51	0.022	-3.88	-2.92
NM_021100	1678	NFS1	protein_coding	0.51	0.040	-0.56	0.40
NM_015312	4373	KIAA1109	protein_coding	0.51	0.044	-6.57	-5.61
NM_001452	1885	FOXF2	protein_coding	0.51	0.031	-5.29	-4.33
NM_022455	7306	NSD1	protein_coding	0.51	0.040	-2.15	-1.19
NM_015340	2995	LARS2	protein_coding	0.51	0.039	-2.29	-1.32
NM_015060	1327	AVL9	protein_coding	0.51	0.040	-6.13	-5.17
NM_001116	4990	ADCY9	protein_coding	0.51	0.038	-2.21	-1.25
NM_017612	2337	ZCCHC8	protein_coding	0.51	0.048	-4.39	-3.43
NM_032857	1702	LACTB	protein_coding	0.51	0.047	-5.78	-4.81
NM_006794	299	GPR75	protein_coding	0.51	0.021	-5.59	-4.62
NM_145034	1604	TOR1AIP2	protein_coding	0.51	0.036	-2.74	-1.77
NM_001099679	2343	TRIM32	protein_coding	0.51	0.029	-6.09	-5.12

NM_001172509	68	SATB2	protein_coding	0.51	0.020	-4.49	-3.51
NM_005779	1768	LHFPL2	protein_coding	0.51	0.021	-4.04	-3.06
NM_024805	1017	RBFA	protein_coding	0.51	0.035	-3.62	-2.64
NM_022461	1609	AZI2	protein_coding	0.51	0.035	-3.61	-2.62
NM_006766	6143	KAT6A	protein_coding	0.51	0.048	-3.48	-2.50
NM_015584	1718	POLDIP2	protein_coding	0.51	0.035	1.29	2.28
NM_016332	956	MSRB1	protein_coding	0.51	0.021	-0.03	0.96
NM_024675	2137	PALB2	protein_coding	0.50	0.009	-6.48	-5.49
NM_001080495	5433	TNRC18	protein_coding	0.50	0.008	-3.32	-2.34
NM_001111	3873	ADAR	protein_coding	0.50	0.023	-3.08	-2.10
NM_198935	4143	SS18L1	protein_coding	0.50	0.004	-5.30	-4.31
NR_040012	1181	SPAG5-AS1	ncRNA	0.50	0.011	-6.39	-5.41
NM_001943	2750	DSG2	protein_coding	0.50	0.038	-4.77	-3.78
NM_033204	983	ZNF101	protein_coding	0.50	0.033	-2.81	-1.82
NM_004697	2570	PRPF4	protein_coding	0.50	0.024	-1.79	-0.79
NM_013318	6956	PRRC2B	protein_coding	0.50	0.027	-4.40	-3.40
NM_021738	1354	SVIL	protein_coding	0.50	0.032	-2.58	-1.58
NM_001017995	6508	SH3PXD2B	protein_coding	0.50	0.020	-2.54	-1.54
NM_003222	1629	TFAP2C	protein_coding	0.50	0.026	-4.61	-3.61
NM_015556	4819	SIPA1L1	protein_coding	0.50	0.035	-6.54	-5.54
NM_001961	242	EEF2	protein_coding	0.50	0.033	-5.32	-4.31
NM_032314	1135	COQ5	protein_coding	0.50	0.029	-5.09	-4.09
NM_025191	2818	EDEM3	protein_coding	0.50	0.035	-5.67	-4.66
NM_001013694	918	SRRD	protein_coding	0.50	0.025	-0.23	0.78
NM_001278346	1027	PHYKPL	protein_coding	0.50	0.019	-6.41	-5.40
NM_024536	1508	CHPF	protein_coding	0.50	0.018	-6.86	-5.85
NM_017840	469	MRPL16	protein_coding	0.49	0.039	-3.69	-2.67
NM_004585	725	RARRES3	protein_coding	0.49	0.034	3.31	4.33
NM_024832	3204	RIN3	protein_coding	0.49	0.022	-2.71	-1.69
NM_006379	2576	SEMA3C	protein_coding	0.49	0.041	-6.84	-5.81
NM_012232	1106	CAVIN1	protein_coding	0.49	0.009	-6.39	-5.36
NM_001190945	2960	TRAF1	protein_coding	0.49	0.009	-4.79	-3.75
NM_015172	1756	PRRC2C	protein_coding	0.49	0.036	-2.85	-1.81
NM_012158	1358	FBXL3	protein_coding	0.49	0.044	-4.90	-3.86
NM_020747	3554	ZNF608	protein_coding	0.48	0.038	-1.65	-0.60
NM_004719	2958	SCAF11	protein_coding	0.48	0.039	-6.01	-4.96
NM_001278182	2462	EOMES	protein_coding	0.48	0.038	-5.54	-4.48
NM_018287	823	ARHGAP12	protein_coding	0.48	0.033	-4.16	-3.11
NM_001244580	11715	TRRAP	protein_coding	0.48	0.025	-4.81	-3.76
NM_031901	201	MRPS21	protein_coding	0.48	0.048	-3.45	-2.39
NM_173566	2178	PRR14L	protein_coding	0.48	0.035	-4.26	-3.20
NM_000437	1746	PAFAH2	protein_coding	0.48	0.044	-4.91	-3.84
NM_030640	2769	DUSP16	protein_coding	0.48	0.016	-1.93	-0.86
NM_004447	2729	EPS8	protein_coding	0.48	0.020	-1.86	-0.79
NM_152703	3842	SAMD9L	protein_coding	0.48	0.013	-5.22	-4.15
NM_006031	8429	PCNT	protein_coding	0.48	0.033	-5.03	-3.95
NM_005515	1360	MNX1	protein_coding	0.48	0.025	-5.83	-4.76
NM_002473	6397	MYH9	protein_coding	0.47	0.042	3.58	4.66
NM_017542	1092	POGK	protein_coding	0.47	0.035	-3.45	-2.37
NM_024792	1262	FAM57A	protein_coding	0.47	0.038	2.71	3.80
NM_153717	6131	EVC	protein_coding	0.47	0.038	-3.87	-2.78

NM_012481	2071	IKZF3	protein_coding	0.47	0.037	-1.96	-0.87
NM_182476	1528	COQ6	protein_coding	0.47	0.029	-0.33	0.77
NM_004566	2728	PFKFB3	protein_coding	0.47	0.032	-2.28	-1.19
NM_018385	2832	LSG1	protein_coding	0.47	0.011	-1.95	-0.85
NM_015042	1431	ZNF609	protein_coding	0.46	0.009	-0.70	0.41
NM_004285	2791	H6PD	protein_coding	0.46	0.035	-3.77	-2.66
NM_032172	3581	USP42	protein_coding	0.46	0.037	-3.78	-2.67
NM_004799	1644	ZFYVE9	protein_coding	0.46	0.033	-6.19	-5.08
NM_020865	3035	DHX36	protein_coding	0.46	0.042	-4.87	-3.75
NM_021953	1337	FOXM1	protein_coding	0.46	0.044	-4.02	-2.90
NM_152857	668	WTAP	protein_coding	0.46	0.037	-3.58	-2.46
NM_007373	3529	SHOC2	protein_coding	0.46	0.012	-6.05	-4.92
NM_014755	1494	SERTAD2	protein_coding	0.46	0.033	-6.52	-5.39
NM_005777	267	RBM6	protein_coding	0.45	0.008	1.60	2.76
NM_001136262	732	ATXN7L3B	protein_coding	0.45	0.045	-4.74	-3.58
NM_002229	1250	JUNB	protein_coding	0.45	0.008	-1.16	0.00
NM_004728	362	DDX21	protein_coding	0.45	0.040	-4.38	-3.21
NM_138704	1052	NSMCE3	protein_coding	0.45	0.041	-6.49	-5.33
NM_001627	425	ALCAM	protein_coding	0.45	0.034	-0.35	0.82
NR_023924	1444	DHRS4-AS1	ncRNA	0.44	0.023	-5.59	-4.42
NM_020727	1035	ZBTB21	protein_coding	0.44	0.030	-4.60	-3.42
NM_016604	1329	KDM3B	protein_coding	0.44	0.036	-4.22	-3.05
NM_001631	962	ALPI	protein_coding	0.44	0.018	-3.61	-2.43
NM_001172818	565	PGM1	protein_coding	0.44	0.033	-4.45	-3.26
NM_032775	1895	KLHL22	protein_coding	0.44	0.025	-5.58	-4.39
NM_003604	3156	IRS4	protein_coding	0.44	0.017	-5.93	-4.74
NM_001199862	1695	KCNAB2	protein_coding	0.44	0.033	-0.33	0.87
NM_017802	3075	DNAAF5	protein_coding	0.43	0.041	-4.32	-3.12
NM_018380	2321	DDX28	protein_coding	0.43	0.032	-5.04	-3.84
NM_004728	305	DDX21	protein_coding	0.43	0.044	-6.42	-5.21
NM_017934	5393	PHIP	protein_coding	0.42	0.007	-4.13	-2.90
NM_024511	1923	HAUS3	protein_coding	0.42	0.038	-3.47	-2.22
NM_024490	4500	ATP10A	protein_coding	0.42	0.041	-0.42	0.85
NM_002375	1631	MAP4	protein_coding	0.41	0.025	-0.04	1.24
NM_017575	1133	SMG6	protein_coding	0.41	0.008	1.10	2.39
NM_001002762	2371	DNAJB12	protein_coding	0.41	0.007	0.52	1.81
NM_016240	1111	SCARA3	protein_coding	0.41	0.006	-3.03	-1.73
NR_047001	1839	RNF219-AS1	ncRNA	0.40	0.048	-6.81	-5.49
NR_038842	554	LINC01137	ncRNA	0.40	0.029	-2.98	-1.65
NM_001206	2090	KLF9	protein_coding	0.40	0.033	-5.22	-3.88
NM_021964	2425	ZNF148	protein_coding	0.39	0.047	3.48	4.82
NM_005230	1633	ELK3	protein_coding	0.39	0.009	-7.08	-5.74
NM_033200	2091	LMF2	protein_coding	0.39	0.038	2.65	4.01
NM_002375	1709	MAP4	protein_coding	0.39	0.035	-0.99	0.37
NM_006923	849	SDF2	protein_coding	0.39	0.020	-4.89	-3.51
NM_021181	2007	SLAMF7	protein_coding	0.38	0.024	-3.44	-2.06
NM_022903	1395	CCDC71	protein_coding	0.38	0.014	-5.63	-4.25
NM_031965	1055	HASPIN	protein_coding	0.38	0.020	-5.48	-4.08
NM_001178099	2583	ZNF182	protein_coding	0.38	0.004	-6.62	-5.21
NM_012482	3361	ZNF281	protein_coding	0.37	0.035	-4.49	-3.05
NM_005626	1202	SRSF4	protein_coding	0.36	0.028	-4.67	-3.19

NM_002706	796	PPM1B	protein_coding	0.36	0.046	-5.46	-3.97
NM_015325	3310	ICE1	protein_coding	0.35	0.039	-7.51	-6.01
NM_058180	615	C21orf58	protein_coding	0.34	0.020	-1.54	0.03
NM_024963	1581	FBXL18	protein_coding	0.33	0.028	0.75	2.35
NM_173079	1183	RUNDC1	protein_coding	0.33	0.024	-0.19	1.41
NM_024095	770	ASB8	protein_coding	0.32	0.046	1.30	2.95
NM_152680	2002	TMEM154	protein_coding	0.32	0.032	-1.41	0.26
NM_001172669	2432	ZNF668	protein_coding	0.31	0.044	-3.40	-1.72
NM_001728	870	BSG	protein_coding	0.31	0.022	-3.15	-1.45
NM_015687	3609	FILIP1	protein_coding	0.30	0.039	1.01	2.75
NM_001174118	734	MEX3D	protein_coding	0.30	0.024	1.16	2.91
NM_152778	1784	MFSD8	protein_coding	0.29	0.030	0.05	1.82
NM_080598	547	DDX39B	protein_coding	0.29	0.043	1.45	3.23
NM_001039999	1798	FAM83G	protein_coding	0.28	0.048	1.11	2.93
NM_005126	1341	NR1D2	protein_coding	0.28	0.031	-5.83	-4.00
NM_001166693	759	AFF1	protein_coding	0.28	0.026	-0.87	0.98
NM_198569	1141	ADGRG6	protein_coding	0.26	0.044	-6.18	-4.26
NM_015057	9251	MYCBP2	protein_coding	0.26	0.017	0.30	2.26
NM_001012991	1551	KNOP1	protein_coding	0.26	0.023	-1.47	0.50
NM_004375	265	COX11	protein_coding	0.25	0.031	-2.98	-0.99
NM_015659	1086	RSL1D1	protein_coding	0.25	0.045	-3.60	-1.58
NM_005371	973	METTL1	protein_coding	0.22	0.047	-1.10	1.06

Supplemental table S2. Methylated transcripts Tocilizumab vs Placebo

**Hypermethylated m6A sites**							
TransID	***m6A transcript location***	***Gene Symbol***	***Trans biotype***	***Fold change***	***P-value (unpaired t-test)***	***Tocilizumab (mean)***	***Placebo (mean)***
NM_201380	12585	PLEC	protein_coding	2.71	0.034	-3.15	-4.58
NM_006038	840	SPATA2	protein_coding	2.39	0.025	-5.08	-6.33
NM_001992	2603	F2R	protein_coding	2.11	0.048	-4.43	-5.51
**Hypormethylated m6A sites**							
TransID	***m6A transcript location***	***Gene Symbol***	***Trans biotype***	***Fold change***	***P-value (unpaired t-test)***	***Tocilizumab (mean)***	***Placebo (mean)***
NM_152260	83	RPUSD2	protein_coding	0.67	0.041	-5.23	-4.65
NM_015020	4127	PHLPP2	protein_coding	0.66	0.005	-4.40	-3.81
NM_003409	1609	ZBTB14	protein_coding	0.66	0.032	-5.26	-4.67
NM_001278182	2462	EOMES	protein_coding	0.66	0.040	-5.70	-5.11
NM_013975	206	LIG3	protein_coding	0.66	0.047	-5.83	-5.24
NM_032865	2407	TNS4	protein_coding	0.66	0.032	-6.05	-5.45
NM_005791	687	MPHOSPH10	protein_coding	0.66	0.044	0.41	1.02
NM_015559	532	SETBP1	protein_coding	0.66	0.048	-5.04	-4.44
NM_004725	190	BUB3	protein_coding	0.65	0.040	4.67	5.28
NM_012208	2232	HARS2	protein_coding	0.65	0.033	-6.26	-5.65
NM_006110	683	CD2BP2	protein_coding	0.65	0.036	-6.34	-5.73
NM_138346	1161	KIAA2013	protein_coding	0.65	0.018	-5.83	-5.21
NM_019044	2343	CCDC93	protein_coding	0.65	0.047	-6.40	-5.78
NM_145058	1184	RILPL2	protein_coding	0.65	0.009	-5.28	-4.65
NM_017712	954	PGPEP1	protein_coding	0.65	0.010	3.21	3.83
NM_003403	1951	YY1	protein_coding	0.65	0.005	-6.10	-5.47

NM_004638_3	3195	PRRC2A	protein_coding	0.65	0.046	1.87	2.50
NM_015038	4827	KIAA0754	protein_coding	0.65	0.036	-6.60	-5.97
NM_005126	1187	NR1D2	protein_coding	0.64	0.017	-5.12	-4.48
NM_021960	914	MCL1	protein_coding	0.64	0.028	-1.69	-1.05
NM_001277817	669	YTHDF3	protein_coding	0.64	0.016	-5.85	-5.21
NM_001002814	6678	RAB11FIP1	protein_coding	0.64	0.022	-3.72	-3.08
NM_001270507	1592	TNFAIP3	protein_coding	0.64	0.018	-6.22	-5.58
NM_003842	1309	TNFRSF10B	protein_coding	0.64	0.039	-6.15	-5.50
NM_000100	415	CSTB	protein_coding	0.64	0.032	-5.50	-4.85
NM_003198	997	ELOA	protein_coding	0.64	0.021	-5.25	-4.60
NM_000416	1615	IFNGR1	protein_coding	0.64	0.037	-6.37	-5.71
NM_017554	2120	PARP14	protein_coding	0.64	0.012	-5.22	-4.57
NM_004863	1919	SPTLC2	protein_coding	0.63	0.037	5.01	5.67
NM_015213	801	DENND5A	protein_coding	0.63	0.042	-6.69	-6.02
NM_014494	1068	TNRC6A	protein_coding	0.63	0.003	-5.14	-4.47
NM_020827	380	CFAP97	protein_coding	0.63	0.040	-5.01	-4.34
NM_024832	3204	RIN3	protein_coding	0.63	0.007	-2.30	-1.63
NM_005514_4	838	HLA-B	protein_coding	0.63	0.014	-2.81	-2.13
NM_002923	778	RGS2	protein_coding	0.63	0.029	-5.32	-4.64
NM_014806	950	RUSC2	protein_coding	0.63	0.029	-5.51	-4.83
NM_022153	1900	VSIR	protein_coding	0.62	0.008	-4.30	-3.63
NM_016006	556	ABHD5	protein_coding	0.62	0.033	-3.95	-3.27
NM_032444	7043	SLX4	protein_coding	0.62	0.007	0.44	1.13
NM_032765	627	TRIM52	protein_coding	0.62	0.041	-5.64	-4.96
NM_001002762	2371	DNAJB12	protein_coding	0.62	0.025	1.26	1.95
NM_001659	156	ARF3	protein_coding	0.62	0.034	-4.14	-3.45
NM_001145299	2266	EXOC7	protein_coding	0.62	0.015	-6.10	-5.40
NM_018990	2572	SASH3	protein_coding	0.62	0.028	-5.00	-4.30
NM_000113	989	TOR1A	protein_coding	0.61	0.046	-5.27	-4.57
NM_001494	1929	GDI2	protein_coding	0.61	0.048	-5.74	-5.03
NM_032182	1297	ABRAXAS2	protein_coding	0.61	0.005	0.37	1.08
NM_020532	191	RTN4	protein_coding	0.61	0.040	4.16	4.88
NM_005253	2064	FOSL2	protein_coding	0.61	0.009	-2.82	-2.10
NM_000591	1245	CD14	protein_coding	0.61	0.012	0.39	1.11
NM_138473	2531	SP1	protein_coding	0.61	0.036	-3.10	-2.37
NM_001134338	706	RNF24	protein_coding	0.61	0.047	-5.81	-5.08
NM_032859	817	ABHD13	protein_coding	0.60	0.048	-6.05	-5.32
NM_006260	2088	DNAJC3	protein_coding	0.60	0.046	-5.95	-5.22
NM_015172	3673	PRRC2C	protein_coding	0.60	0.037	-7.62	-6.89
NM_015946	1985	PELO	protein_coding	0.60	0.030	-6.05	-5.31
NM_138473	1159	SP1	protein_coding	0.60	0.015	-5.21	-4.48
NM_003204	180	NFE2L1	protein_coding	0.60	0.012	-5.53	-4.79
NM_203390	948	RBM12B	protein_coding	0.60	0.012	-5.92	-5.18
NM_001080477	6957	TENM3	protein_coding	0.60	0.024	-2.70	-1.96
NM_020727	229	ZBTB21	protein_coding	0.60	0.006	-2.33	-1.58
NM_145037	2287	NXPE3	protein_coding	0.59	0.031	-6.34	-5.59
NM_007186	7054	CEP250	protein_coding	0.59	0.024	-4.40	-3.65
NM_001281453	1493	MBD3	protein_coding	0.59	0.019	-6.04	-5.29
NM_004514	3032	FOXK2	protein_coding	0.59	0.029	-2.37	-1.61
NM_017669	1998	ERCC6L	protein_coding	0.59	0.036	-6.74	-5.98
NM_024490	4500	ATP10A	protein_coding	0.59	0.010	0.10	0.86

NM_014892	3155	SCAF8	protein_coding	0.59	0.011	-5.49	-4.72
NM_001271938	8097	MEGF8	protein_coding	0.58	0.010	-6.71	-5.93
NM_003363	2960	USP4	protein_coding	0.58	0.002	-5.75	-4.97
NM_016448	1276	DTL	protein_coding	0.58	0.039	-5.25	-4.46
NM_182476	1528	COQ6	protein_coding	0.58	0.001	0.25	1.03
NM_014415	3078	ZBTB11	protein_coding	0.58	0.040	-7.33	-6.54
NM_015556	4819	SIPA1L1	protein_coding	0.57	0.050	-6.76	-5.96
NM_001821	2201	CHML	protein_coding	0.57	0.048	-4.13	-3.32
NM_002298	3356	LCP1	protein_coding	0.57	0.003	-4.58	-3.77
NM_001098832	2586	FAM104A	protein_coding	0.57	0.029	-6.30	-5.48
NM_024597	1346	MAP7D3	protein_coding	0.57	0.030	-4.61	-3.80
NM_001893	1982	CSNK1D	protein_coding	0.56	0.032	-4.20	-3.37
NM_006682	767	FGL2	protein_coding	0.56	0.005	-6.46	-5.62
NM_001017405	1737	MAEA	protein_coding	0.56	0.020	-5.53	-4.70
NM_006493	687	CLN5	protein_coding	0.56	0.045	-5.74	-4.90
NM_002296	3656	LBR	protein_coding	0.56	0.048	-5.16	-4.32
NM_001270439	346	ARPC5	protein_coding	0.56	0.035	-6.12	-5.28
NM_024565	1646	CCNJL	protein_coding	0.56	0.028	-6.28	-5.44
NM_032251	4892	CCDC88B	protein_coding	0.56	0.004	-1.78	-0.94
NM_021035	5451	ZNFX1	protein_coding	0.56	0.043	-6.32	-5.47
NM_015492	296	C15orf39	protein_coding	0.55	0.000	-4.11	-3.25
NM_001111	3873	ADAR	protein_coding	0.55	0.019	-2.90	-2.04
NM_021960	665	MCL1	protein_coding	0.55	0.004	-2.85	-1.99
NM_018169	1751	RESF1	protein_coding	0.55	0.025	-6.08	-5.22
NM_014779	1624	TSC22D2	protein_coding	0.55	0.031	-5.46	-4.59
NM_004385	9212	VCAN	protein_coding	0.55	0.048	-5.39	-4.52
NM_003264	734	TLR2	protein_coding	0.55	0.015	-7.29	-6.42
NM_001080825	5056	TMEM120B	protein_coding	0.54	0.007	2.53	3.40
NM_020865	3132	DHX36	protein_coding	0.54	0.043	-5.57	-4.69
NM_133264	1762	WIPF2	protein_coding	0.54	0.017	-6.91	-6.03
NM_002778	2309	PSAP	protein_coding	0.54	0.031	-3.85	-2.97
NM_148894	933	BOD1L1	protein_coding	0.54	0.041	-5.89	-5.01
NM_001199862	1695	KCNAB2	protein_coding	0.54	0.009	-0.17	0.71
NM_001002814	546	RAB11FIP1	protein_coding	0.54	0.046	-6.42	-5.53
NM_001110	2916	ADAM10	protein_coding	0.54	0.018	-6.32	-5.43
NM_015299	2195	KHNYN	protein_coding	0.54	0.042	-7.24	-6.35
NM_178014_6	913	TUBB	protein_coding	0.54	0.033	-6.78	-5.88
NM_181847	839	AMIGO2	protein_coding	0.54	0.029	-7.36	-6.46
NM_144628	3966	TBC1D20	protein_coding	0.54	0.003	-4.22	-3.32
NM_001002032	592	JPT1	protein_coding	0.53	0.040	-5.96	-5.05
NM_144628	4253	TBC1D20	protein_coding	0.53	0.020	-1.40	-0.48
NM_001265589	2942	RTN3	protein_coding	0.53	0.033	-1.62	-0.71
NM_001112736	2533	FAM208A	protein_coding	0.53	0.041	-6.82	-5.90
NM_006031	3103	PCNT	protein_coding	0.53	0.045	-6.12	-5.20
NM_031445	1905	AMMECR1L	protein_coding	0.53	0.009	0.84	1.76
NM_001099409	1475	EHBP1L1	protein_coding	0.53	0.044	-7.07	-6.15
NR_152871	6824	TUG1	ncRNA	0.53	0.018	-6.83	-5.90
NM_005733	3064	KIF20A	protein_coding	0.52	0.018	-6.61	-5.68
NM_001001410	1022	TSR3	protein_coding	0.52	0.049	-6.66	-5.72
NM_001145409	1935	NONO	protein_coding	0.52	0.042	-5.71	-4.77
NM_181701	1851	QSOX2	protein_coding	0.52	0.042	3.35	4.28

NM_022051	3904	EGLN1	protein_coding	0.52	0.032	-5.26	-4.32
NM_199512	1726	CCDC80	protein_coding	0.52	0.048	-4.95	-4.01
NM_002838	3970	PTPRC	protein_coding	0.52	0.024	-6.60	-5.66
NM_001277223	145	TAGLN2	protein_coding	0.52	0.007	-6.37	-5.42
NM_014762	3093	DHCR24	protein_coding	0.52	0.012	0.70	1.64
NM_022727	756	TRMT2A	protein_coding	0.52	0.047	-6.47	-5.52
NM_001789	3250	CDC25A	protein_coding	0.52	0.008	-7.39	-6.44
NM_018287	823	ARHGAP12	protein_coding	0.52	0.021	-4.52	-3.56
NM_003718	4611	CDK13	protein_coding	0.52	0.026	-5.47	-4.51
NM_001025091	2661	ABCF1	protein_coding	0.51	0.011	3.44	4.40
NM_021960	1131	MCL1	protein_coding	0.51	0.013	-4.20	-3.24
NM_152362	802	TNFAIP8L1	protein_coding	0.51	0.027	1.58	2.54
NM_015001	3367	SPEN	protein_coding	0.51	0.023	-5.47	-4.50
NM_015322	1889	FEM1B	protein_coding	0.51	0.037	-6.13	-5.15
NM_001415	2322	EIF2S3	protein_coding	0.51	0.035	-1.41	-0.44
NM_006454	510	MXD4	protein_coding	0.50	0.004	1.90	2.89
NM_002375	1709	MAP4	protein_coding	0.50	0.001	-0.60	0.40
NM_018029	727	EBLN2	protein_coding	0.50	0.026	-7.21	-6.21
NM_012288	6714	TRAM2	protein_coding	0.50	0.022	-5.59	-4.59
NM_024620	839	ZNF329	protein_coding	0.50	0.036	-4.20	-3.19
NM_005729	659	PPIF	protein_coding	0.50	0.039	-6.06	-5.05
NM_080491	2828	GAB2	protein_coding	0.50	0.011	-3.87	-2.86
NM_001111	1186	ADAR	protein_coding	0.49	0.011	-7.15	-6.13
NM_021960	1577	MCL1	protein_coding	0.49	0.032	-0.70	0.33
NM_152680	2002	TMEM154	protein_coding	0.49	0.002	-0.97	0.07
NM_001116	4642	ADCY9	protein_coding	0.48	0.021	0.70	1.75
NM_006939	4161	SOS2	protein_coding	0.48	0.003	-6.24	-5.19
NM_032859	1095	ABHD13	protein_coding	0.48	0.042	0.12	1.19
NM_002375	1631	MAP4	protein_coding	0.48	0.000	0.53	1.60
NM_001099679	1494	TRIM32	protein_coding	0.48	0.013	-5.72	-4.65
NM_014827	2028	ZC3H11A	protein_coding	0.48	0.043	-7.42	-6.35
NM_001111	360	ADAR	protein_coding	0.48	0.021	1.95	3.02
NM_001136472	767	LITAF	protein_coding	0.47	0.017	-4.04	-2.97
NM_198935	4143	SS18L1	protein_coding	0.47	0.013	-5.43	-4.35
NM_030819	1486	GFOD2	protein_coding	0.47	0.029	-7.44	-6.37
NM_205860	2186	NR5A2	protein_coding	0.47	0.026	-6.34	-5.26
NM_001682	4312	ATP2B1	protein_coding	0.47	0.018	-5.85	-4.76
NM_015299	908	KHNYN	protein_coding	0.47	0.046	3.52	4.62
NM_153717	6131	EVC	protein_coding	0.47	0.021	-4.10	-3.00
NM_145039	2837	CENPBD1	protein_coding	0.47	0.047	-7.15	-6.05
NM_006327	861	TIMM23	protein_coding	0.46	0.034	-5.19	-4.08
NR_002819	1994	MALAT1	ncRNA	0.46	0.014	-5.90	-4.78
NM_020412	468	CHMP1B	protein_coding	0.46	0.013	-5.35	-4.23
NM_001136223	1958	RCOR3	protein_coding	0.46	0.014	-5.88	-4.75
NM_080491	2792	GAB2	protein_coding	0.46	0.031	-5.80	-4.66
NM_017668	1918	NDE1	protein_coding	0.45	0.000	-5.94	-4.80
NM_018247	1947	TMEM30A	protein_coding	0.44	0.039	-7.19	-6.02
NM_022100	590	MRPS14	protein_coding	0.44	0.049	-5.59	-4.41
NM_018050	1555	MANSC1	protein_coding	0.44	0.043	-7.35	-6.15
NM_001040438_3	537	C6orf48	protein_coding	0.43	0.028	-0.55	0.67
NM_002535	2233	OAS2	protein_coding	0.43	0.021	-5.12	-3.90

NM_021964	2490	ZNF148	protein_coding	0.43	0.034	-7.18	-5.96
NM_000043	1267	FAS	protein_coding	0.43	0.001	-6.74	-5.51
NM_006825	1798	CKAP4	protein_coding	0.43	0.032	-6.42	-5.18
NM_001002860	3381	BTBD7	protein_coding	0.42	0.026	1.63	2.86
NM_001067	5340	TOP2A	protein_coding	0.42	0.035	-7.21	-5.97
NM_006939	1717	SOS2	protein_coding	0.42	0.006	-5.49	-4.25
NM_001008397	1075	GPX8	protein_coding	0.42	0.006	-7.57	-6.33
NM_130386	2495	COLEC12	protein_coding	0.42	0.008	-6.95	-5.69
NM_022461	1568	AZI2	protein_coding	0.42	0.026	-7.09	-5.83
NM_018380	2497	DDX28	protein_coding	0.42	0.048	-7.04	-5.78
NM_005908	2621	MANBA	protein_coding	0.41	0.014	-7.56	-6.28
NM_001018072	4048	BTBD11	protein_coding	0.41	0.048	-6.86	-5.57
NM_020120	4859	UGGT1	protein_coding	0.41	0.049	-6.50	-5.21
NM_033200	2091	LMF2	protein_coding	0.41	0.042	2.69	3.99
NM_021964	2425	ZNF148	protein_coding	0.41	0.037	3.60	4.90
NM_025160	2391	WDR26	protein_coding	0.40	0.039	-5.67	-4.36
NM_005030	1256	PLK1	protein_coding	0.40	0.023	0.85	2.16
NM_138792	348	LEO1	protein_coding	0.40	0.027	-6.82	-5.49
NM_014706	2993	SART3	protein_coding	0.39	0.017	1.34	2.68
NM_024095	770	ASB8	protein_coding	0.39	0.020	1.15	2.51
NM_148898	2628	FOXP2	protein_coding	0.39	0.040	-7.32	-5.96
NM_006098	617	RACK1	protein_coding	0.39	0.005	1.93	3.29
NM_002649	2106	PIK3CG	protein_coding	0.38	0.011	-7.08	-5.70
NR_002819	1935	MALAT1	ncRNA	0.38	0.029	-4.77	-3.39
NM_001111	1063	ADAR	protein_coding	0.37	0.000	-7.67	-6.24
NM_006082	1640	TUBA1B	protein_coding	0.37	0.019	-1.07	0.37
NM_004996	5895	ABCC1	protein_coding	0.36	0.024	-4.73	-3.27
NM_001127395	723	METTL21A	protein_coding	0.36	0.015	-7.50	-6.03
NM_001162498	2198	LPAR6	protein_coding	0.36	0.014	-7.33	-5.85
NM_001005920	815	JMJD8	protein_coding	0.36	0.023	3.15	4.63
NM_005431	2299	XRCC2	protein_coding	0.36	0.010	-7.22	-5.73
NR_131012	933	NEAT1	ncRNA	0.35	0.019	0.27	1.77
NM_006858	870	TMED1	protein_coding	0.35	0.009	-0.70	0.80
NM_003380	1782	VIM	protein_coding	0.35	0.005	-1.35	0.16
NR_152406	1256	CUTALP	ncRNA	0.35	0.008	-7.38	-5.87
NM_014657	1313	TTI1	protein_coding	0.35	0.047	-3.65	-2.13
NM_080598	547	DDX39B	protein_coding	0.34	0.019	1.62	3.16
NM_003740	1685	KCNK5	protein_coding	0.34	0.046	-7.58	-6.00
NM_015057	9251	MYCBP2	protein_coding	0.33	0.003	0.45	2.06
NM_020120	4883	UGGT1	protein_coding	0.33	0.017	-6.99	-5.37
NM_058180	615	C21orf58	protein_coding	0.33	0.004	-1.43	0.19
NM_003257	3741	TJP1	protein_coding	0.32	0.043	1.31	2.94
NM_015687	3609	FILIP1	protein_coding	0.32	0.005	1.19	2.83
NM_003380	1817	VIM	protein_coding	0.32	0.019	-6.27	-4.63
NM_006031	6666	PCNT	protein_coding	0.31	0.028	1.58	3.26
NM_006302	1355	MOGS	protein_coding	0.31	0.035	2.00	3.68
NM_001627	425	ALCAM	protein_coding	0.31	0.003	-0.18	1.53
NM_173079	1183	RUNDC1	protein_coding	0.28	0.008	0.20	2.03
NM_018334	2097	LRRN3	protein_coding	0.28	0.044	-6.60	-4.75
NM_001031623	1891	ZNF451	protein_coding	0.25	0.044	-7.56	-5.54
NM_006907	1932	PYCR1	protein_coding	0.25	0.045	-3.25	-1.23

NM_014345	6534	ZNF318	protein_coding	0.24	0.009	-0.96	1.11
NM_001039999	1798	FAM83G	protein_coding	0.23	0.020	0.86	2.96
NM_145059	653	FUK	protein_coding	0.23	0.050	1.93	4.04
NM_001166693	759	AFF1	protein_coding	0.23	0.006	-0.84	1.30
NM_181552	1668	CUX1	protein_coding	0.22	0.047	-2.72	-0.57
NM_175038	3218	CNTN1	protein_coding	0.21	0.009	-1.57	0.68
NM_001012991	1551	KNOP1	protein_coding	0.20	0.006	-2.30	0.04
NM_001040446	1916	MTMR12	protein_coding	0.18	0.026	-0.41	2.06
NM_005371	973	METTL1	protein_coding	0.18	0.016	-1.43	1.05
NM_020761	6552	RPTOR	protein_coding	0.08	0.013	-2.47	1.10

Supplemental figure S3 transcripts with significantly different m6A sites and DEGs between HC and STEMI

m6A methylation array (hypomethylated)							RNAsequensing		
**TransID**	**Gene Symbol**	**m6A location**	**m6A transcript location**	**Trans biotype**	**Fold change**	**P-value (unpaired t-test)**	Gene ID Version	**log2 Fold Change**	p-value
NM_053052	SNAP47	3'UTR	2226	protein_coding	**0.67**	0.026	ENSG00000143740.15	**-0.29**	0.007
NM_001080826	PRAG1	CDS	3070	protein_coding	**0.67**	0.008	ENSG00000275342.5	**-0.48**	0.002
NM_001098832	FAM104A	3'UTR	1922	protein_coding	**0.67**	0.043	ENSG00000133193.12	**-0.45**	0.016
NM_024562	TANGO6	3'UTR	3600	protein_coding	**0.67**	0.009	ENSG00000103047.8	**-0.28**	0.046
NM_015885	PCF11	CDS	2732	protein_coding	**0.67**	0.031	ENSG00000165494.11	**0.30**	0.005
NM_000967	RPL3	CDS	266	protein_coding	**0.67**	0.017	ENSG00000100316.16	**-0.33**	0.020
NM_020195	SDR39U1	3'UTR	1096	protein_coding	**0.67**	0.017	ENSG00000100445.17	**-0.24**	0.020
NM_001122772	AGAP2	3'UTR	3894	protein_coding	**0.67**	0.015	ENSG00000135439.11	**0.43**	0.002
NM_001360	DHCR7	3'UTR	1994	protein_coding	**0.66**	0.034	ENSG00000172893.16	**0.70**	0.005
NM_005094	SLC27A4	3'UTR	2513	protein_coding	**0.66**	0.034	ENSG00000167114.13	**-0.39**	0.002
NM_004295	TRAF4	3'UTR	1775	protein_coding	**0.66**	0.048	ENSG00000076604.15	**-0.74**	0.000
NM_017905	TMCO3	CDS	821	protein_coding	**0.66**	0.019	ENSG00000150403.18	**0.83**	0.000
NM_001242840	GUK1	3'UTR	863	protein_coding	**0.66**	0.028	ENSG00000143774.17	**-0.62**	0.004
NM_016436	PHF20	3'UTR	3893	protein_coding	**0.66**	0.040	ENSG00000025293.17	**0.76**	0.000
NM_007300	BRCA1	3'UTR	6408	protein_coding	**0.66**	0.008	ENSG00000012048.23	**0.63**	0.003
NM_015270	ADCY6	3'UTR	3876	protein_coding	**0.66**	0.009	ENSG00000174233.12	**-0.92**	0.011
NM_001303525	TUBB6	3'UTR	713	protein_coding	**0.66**	0.021	ENSG00000176014.13	**-0.80**	0.002
NM_020745	AARS2	3'UTR	3673	protein_coding	**0.66**	0.006	ENSG00000124608.5	**-0.28**	0.026
NM_001142292	LMAN2L	3'UTR	1292	protein_coding	**0.66**	0.018	ENSG00000114988.12	**-0.51**	0.001
NM_004793	LONP1	CDS	2936	protein_coding	**0.66**	0.021	ENSG00000196365.12	**-0.30**	0.030
NM_001083600	NAA60	3'UTR	1359	protein_coding	**0.66**	0.017	ENSG00000122390.19	**0.25**	0.022
NM_006598	SLC12A7	3'UTR	3701	protein_coding	**0.66**	0.024	ENSG00000113504.21	**-0.59**	0.010
NM_001008895	CUL4A	3'UTR	2432	protein_coding	**0.66**	0.038	ENSG00000139842.15	**-0.35**	0.001

NM_000757	CSF1	3'UTR	2583	protein_coding	**0.66**	0.023	ENSG00000184371.14	**-0.92**	0.000
NM_014938	MLXIP	3'UTR	3918	protein_coding	**0.66**	0.009	ENSG00000175727.14	**0.24**	0.030
NM_001429	EP300	CDS	773	protein_coding	**0.66**	0.015	ENSG00000100393.14	**0.68**	0.000
NM_000127	EXT1	CDS	1257	protein_coding	**0.66**	0.017	ENSG00000182197.12	**0.48**	0.002
NM_032479	MRPL36	CDS	265	protein_coding	**0.66**	0.028	ENSG00000171421.13	**-0.50**	0.043
NM_018052	VAC14	3'UTR	2667	protein_coding	**0.66**	0.033	ENSG00000103043.15	**-0.28**	0.007
NM_017703	FBXL12	3'UTR	1605	protein_coding	**0.66**	0.008	ENSG00000127452.9	**-0.31**	0.030
NM_007317	KIF22	CDS	1107	protein_coding	**0.66**	0.025	ENSG00000079616.13	**-0.40**	0.000
NM_033387	FAM78A	3'UTR	3803	protein_coding	**0.66**	0.015	ENSG00000126882.13	**-0.41**	0.000
NM_001079539	XBP1	CDS	861	protein_coding	**0.66**	0.011	ENSG00000100219.16	**-0.42**	0.001
NM_003045	SLC7A1	3'UTR	2540	protein_coding	**0.65**	0.017	ENSG00000139514.13	**-0.40**	0.015
NM_013276	SHPK	3'UTR	1696	protein_coding	**0.65**	0.024	ENSG00000197417.9	**-0.30**	0.020
NM_022106	FAM217B	CDS	845	protein_coding	**0.65**	0.018	ENSG00000196227.11	**0.68**	0.000
NM_004556	NFKBIE	3'UTR	1958	protein_coding	**0.65**	0.007	ENSG00000146232.17	**-1.02**	0.000
NR_015431	LINC-PINT	517	517	ncRNA	**0.65**	0.024	ENSG00000231721.7	**0.74**	0.000
NM_003129	SQLE	CDS	1373	protein_coding	**0.65**	0.013	ENSG00000104549.12	**-0.58**	0.006
NM_020762	SRGAP1	CDS	3256	protein_coding	**0.65**	0.013	ENSG00000196935.9	**2.55**	0.000
NM_003592	CUL1	3'UTR	2847	protein_coding	**0.65**	0.024	ENSG00000055130.17	**-0.26**	0.032
NM_001005361	DNM2	CDS	1555	protein_coding	**0.65**	0.010	ENSG00000079805.18	**0.71**	0.000
NM_001010924	FAM171A1	3'UTR	3342	protein_coding	**0.65**	0.026	ENSG00000148468.17	**-0.71**	0.011
NM_001183	ATP6AP1	3'UTR	1687	protein_coding	**0.65**	0.005	ENSG00000071553.18	**0.40**	0.000
NM_006088	TUBB4B	CDS	364	protein_coding	**0.65**	0.033	ENSG00000188229.6	**-0.26**	0.049
NM_006379	SEMA3C	CDS	2789	protein_coding	**0.65**	0.047	ENSG00000075223.14	**1.04**	0.000
NM_004824	CDYL	CDS	702	protein_coding	**0.65**	0.018	ENSG00000153046.18	**-0.61**	0.001
NM_015172	PRRC2C	CDS	8582	protein_coding	**0.65**	0.019	ENSG00000117523.16	**0.37**	0.001
NM_001257098	RHNO1	CDS	603	protein_coding	**0.65**	0.011	ENSG00000171792.11	**-0.42**	0.011
NM_001256183	ANKRD11	CDS	3183	protein_coding	**0.65**	0.008	ENSG00000167522.16	**0.63**	0.000
NM_002017	FLI1	CDS	1379	protein_coding	**0.65**	0.034	ENSG00000151702.17	**0.26**	0.038

NM_004085	TIMM8A	3'UTR	487	protein_coding	**0.65**	0.025	ENSG00000126953.8	**-0.41**	0.043
NM_003977	AIP	CDS	1122	protein_coding	**0.65**	0.018	ENSG00000110711.10	**-0.41**	0.000
NM_012111	AHSA1	3'UTR	1144	protein_coding	**0.65**	0.035	ENSG00000100591.8	**-0.49**	0.001
NM_021181	SLAMF7	3'UTR	2625	protein_coding	**0.65**	0.009	ENSG00000026751.17	**-0.57**	0.012
NM_001270507	TNFAIP3	CDS	2144	protein_coding	**0.65**	0.009	ENSG00000118503.15	**1.15**	0.000
NM_023012	RSRC2	CDS	714	protein_coding	**0.65**	0.013	ENSG00000111011.18	**0.26**	0.025
NM_005669	REEP5	CDS	494	protein_coding	**0.65**	0.037	ENSG00000129625.13	**0.33**	0.007
NM_030665	RAI1	CDS	5123	protein_coding	**0.65**	0.037	ENSG00000108557.19	**-0.34**	0.017
NM_015666	MTG2	CDS	903	protein_coding	**0.65**	0.028	ENSG00000101181.18	**0.48**	0.000
NM_001242783_6	TRIM26	CDS	1277	protein_coding	**0.65**	0.032	ENSG00000234127.9	**-0.19**	0.009
NM_018890	RAC1	3'UTR	1300	protein_coding	**0.65**	0.035	ENSG00000136238.18	**0.47**	0.000
NM_005811	GDF11	3'UTR	2921	protein_coding	**0.65**	0.024	ENSG00000135414.10	**-0.55**	0.019
NM_017617	NOTCH1	CDS	6836	protein_coding	**0.65**	0.045	ENSG00000148400.13	**0.44**	0.007
NM_020649	CBX8	CDS	876	protein_coding	**0.65**	0.032	ENSG00000141570.11	**0.56**	0.023
NM_005990	STK10	CDS	1825	protein_coding	**0.65**	0.042	ENSG00000072786.13	**0.40**	0.000
NM_032316	NICN1	3'UTR	967	protein_coding	**0.65**	0.029	ENSG00000145029.14	**-0.74**	0.003
NM_021241	WIZ	CDS	1951	protein_coding	**0.65**	0.019	ENSG00000011451.21	**0.33**	0.046
NM_002649	PIK3CG	CDS	1835	protein_coding	**0.65**	0.029	ENSG00000105851.11	**0.51**	0.000
NM_017772	TBC1D22B	3'UTR	1941	protein_coding	**0.64**	0.009	ENSG00000065491.8	**-0.47**	0.004
NM_003488	AKAP1	CDS	919	protein_coding	**0.64**	0.017	ENSG00000121057.13	**-0.30**	0.049
NM_001218	CA12	3'UTR	1304	protein_coding	**0.64**	0.044	ENSG00000074410.14	**1.71**	0.021
NM_173551	ANKS6	3'UTR	6929	protein_coding	**0.64**	0.038	ENSG00000165138.18	**-0.66**	0.009
NM_004417	DUSP1	CDS	1294	protein_coding	**0.64**	0.038	ENSG00000120129.6	**1.83**	0.000
NM_177542	SNRPD2	5'UTR	142	protein_coding	**0.64**	0.037	ENSG00000125743.11	**-0.39**	0.010
NM_006360	EIF3M	CDS	314	protein_coding	**0.64**	0.050	ENSG00000149100.13	**-0.24**	0.019
NM_016604	KDM3B	CDS	1873	protein_coding	**0.64**	0.035	ENSG00000120733.14	**0.76**	0.000
NM_005148	UNC119	3'UTR	1060	protein_coding	**0.64**	0.033	ENSG00000109103.12	**0.41**	0.001
NM_144775	SMCR8	3'UTR	3336	protein_coding	**0.64**	0.018	ENSG00000176994.11	**0.30**	0.001

NM_006923	SDF2	3'UTR	1314	protein_coding	**0.64**	0.023	ENSG00000132581.10	**0.38**	0.006
NM_183047	ZMYND8	3'UTR	4418	protein_coding	**0.64**	0.019	ENSG00000101040.19	**0.33**	0.006
NM_018036	ATG2B	3'UTR	6770	protein_coding	**0.64**	0.017	ENSG00000066739.12	**0.40**	0.007
NM_001080414	CCDC88C	CDS	1928	protein_coding	**0.64**	0.033	ENSG00000015133.19	**-0.28**	0.043
NM_024496	IRF2BPL	CDS	939	protein_coding	**0.64**	0.026	ENSG00000119669.5	**0.66**	0.001
NM_001243439	SPECC1	CDS	752	protein_coding	**0.64**	0.036	ENSG00000128487.18	**-0.48**	0.002
NM_001025091	ABCF1	3'UTR	2661	protein_coding	**0.64**	0.035	ENSG00000204574.14	**-0.50**	0.000
NM_001261834	TBRG4	3'UTR	2143	protein_coding	**0.64**	0.045	ENSG00000136270.14	**-0.36**	0.012
NM_001145928	SAP130	CDS	3328	protein_coding	**0.64**	0.024	ENSG00000136715.19	**0.42**	0.025
NM_053043	RBM33	CDS	3240	protein_coding	**0.64**	0.015	ENSG00000184863.11	**0.23**	0.034
NM_002886	RAP2B	CDS	927	protein_coding	**0.64**	0.032	ENSG00000181467.5	**-0.26**	0.000
NM_172206	CAMKK1	3'UTR	2755	protein_coding	**0.64**	0.027	ENSG00000004660.15	**0.93**	0.000
NM_012204	GTF3C4	CDS	2021	protein_coding	**0.64**	0.002	ENSG00000125484.12	**-0.35**	0.001
NM_018461	PPP2R2D	3'UTR	1496	protein_coding	**0.64**	0.046	ENSG00000175470.20	**0.20**	0.021
NM_001002295	GATA3	3'UTR	2127	protein_coding	**0.64**	0.020	ENSG00000107485.18	**-0.53**	0.002
NM_181489	ZNF445	3'UTR	3771	protein_coding	**0.64**	0.017	ENSG00000185219.17	**0.98**	0.000
NM_003749	IRS2	3'UTR	5104	protein_coding	**0.64**	0.042	ENSG00000185950.9	**2.26**	0.000
NM_001184740	CTTN	CDS	1777	protein_coding	**0.64**	0.018	ENSG00000085733.16	**-0.50**	0.017
NM_001136262	ATXN7L3B	3'UTR	1113	protein_coding	**0.64**	0.030	ENSG00000253719.4	**-0.31**	0.001
NM_012401	PLXNB2	CDS	799	protein_coding	**0.64**	0.034	ENSG00000196576.16	**-0.40**	0.005
NM_001134338	RNF24	3'UTR	771	protein_coding	**0.64**	0.030	ENSG00000101236.17	**0.62**	0.001
NM_020772	NUFIP2	CDS	853	protein_coding	**0.64**	0.027	ENSG00000108256.9	**0.28**	0.020
NM_014071	NCOA6	CDS	5872	protein_coding	**0.63**	0.012	ENSG00000198646.14	**0.34**	0.001
NM_014643	ZNF516	CDS	1450	protein_coding	**0.63**	0.018	ENSG00000101493.11	**1.01**	0.000
NM_020899	ZBTB4	3'UTR	5409	protein_coding	**0.63**	0.023	ENSG00000174282.12	**-0.47**	0.000
NM_001012241	MSL1	CDS	1101	protein_coding	**0.63**	0.011	ENSG00000188895.12	**0.70**	0.000
NM_005216	DDOST	CDS	780	protein_coding	**0.63**	0.045	ENSG00000244038.11	**-0.19**	0.015
NM_005647	TBL1X	3'UTR	4535	protein_coding	**0.63**	0.029	ENSG00000101849.17	**0.75**	0.000

NM_017929	PEX26	3'UTR	1319	protein_coding	**0.63**	0.010	ENSG00000215193.14	**-0.48**	0.000
NM_024121	TMEM185B	CDS	913	protein_coding	**0.63**	0.025	ENSG00000226479.4	**0.46**	0.001
NM_000852	GSTP1	CDS	875	protein_coding	**0.63**	0.031	ENSG00000084207.18	**-0.57**	0.000
NM_001242739	ZNF691	CDS	1225	protein_coding	**0.63**	0.047	ENSG00000164011.18	**-0.38**	0.008
NM_014777	URB2	CDS	3663	protein_coding	**0.63**	0.024	ENSG00000135763.10	**-0.40**	0.024
NM_182557	BCL9L	CDS	2211	protein_coding	**0.63**	0.044	ENSG00000186174.12	**-0.53**	0.000
NM_144775	SMCR8	CDS	2804	protein_coding	**0.63**	0.042	ENSG00000176994.11	**0.30**	0.001
NM_001252119	PASK	CDS	2490	protein_coding	**0.63**	0.014	ENSG00000115687.14	**-0.57**	0.008
NM_032182	ABRAXAS2	3'UTR	1297	protein_coding	**0.63**	0.049	ENSG00000165660.8	**-0.37**	0.000
NM_018984	SSH1	3'UTR	3700	protein_coding	**0.63**	0.007	ENSG00000084112.15	**0.57**	0.000
NM_014870	ZBTB40	3'UTR	6742	protein_coding	**0.63**	0.003	ENSG00000184677.18	**-0.31**	0.003
NM_006401	ANP32B	3'UTR	992	protein_coding	**0.63**	0.009	ENSG00000136938.9	**-0.56**	0.000
NM_001810	CENPB	CDS	1051	protein_coding	**0.63**	0.040	ENSG00000125817.8	**-0.65**	0.000
NM_015210	MTCL1	CDS	4594	protein_coding	**0.63**	0.021	ENSG00000168502.17	**-1.01**	0.005
NM_001163809	WDR81	CDS	2077	protein_coding	**0.63**	0.020	ENSG00000167716.19	**-0.29**	0.016
NM_015282	CLASP1	3'UTR	7105	protein_coding	**0.63**	0.028	ENSG00000074054.20	**0.59**	0.000
NM_002254	KIF3C	3'UTR	4789	protein_coding	**0.63**	0.032	ENSG00000084731.15	**0.62**	0.004
NM_006977	ZBTB25	CDS	867	protein_coding	**0.63**	0.047	ENSG00000089775.12	**-0.44**	0.003
NM_001408	CELSR2	CDS	2744	protein_coding	**0.63**	0.033	ENSG00000143126.8	**-0.52**	0.017
NM_017798	YTHDF1	CDS	563	protein_coding	**0.63**	0.019	ENSG00000149658.18	**-0.23**	0.001
NM_014286	NCS1	3'UTR	1005	protein_coding	**0.63**	0.010	ENSG00000107130.10	**-0.96**	0.036
NM_016332	MSRB1	3'UTR	901	protein_coding	**0.63**	0.045	ENSG00000198736.12	**0.79**	0.000
NM_001244249	APEX1	CDS	1078	protein_coding	**0.63**	0.029	ENSG00000100823.12	**-0.46**	0.001
NM_001240	CCNT1	CDS	1938	protein_coding	**0.63**	0.049	ENSG00000129315.11	**0.33**	0.039
NM_182557	BCL9L	CDS	5055	protein_coding	**0.63**	0.026	ENSG00000186174.12	**-0.53**	0.000
NM_003036	SKI	3'UTR	2738	protein_coding	**0.62**	0.008	ENSG00000157933.10	**0.43**	0.003
NM_183047	ZMYND8	CDS	2495	protein_coding	**0.62**	0.023	ENSG00000101040.19	**0.33**	0.006
NM_025010	KLHL18	CDS	1727	protein_coding	**0.62**	0.037	ENSG00000114648.12	**0.34**	0.006

NM_001080414	CCDC88C	CDS	2119	protein_coding	**0.62**	0.028	ENSG00000015133.19	**-0.28**	0.043
NM_025137	SPG11	CDS	5563	protein_coding	**0.62**	0.027	ENSG00000104133.15	**0.81**	0.000
NM_152362	TNFAIP8L1	3'UTR	802	protein_coding	**0.62**	0.013	ENSG00000185361.9	**-0.86**	0.000
NM_016333	SRRM2	CDS	2061	protein_coding	**0.62**	0.027	ENSG00000167978.17	**0.36**	0.002
NM_021962	ABR	CDS	2798	protein_coding	**0.62**	0.045	ENSG00000159842.15	**0.33**	0.013
NM_021826	FASTKD5	5'UTR	240	protein_coding	**0.62**	0.024	ENSG00000215251.4	**0.23**	0.050
NM_020440	PTGFRN	3'UTR	5720	protein_coding	**0.62**	0.042	ENSG00000134247.10	**-0.99**	0.024
NM_018994	FBXO42	CDS	2086	protein_coding	**0.62**	0.031	ENSG00000037637.11	**0.30**	0.001
NM_001017963	HSP90AA1	CDS	2490	protein_coding	**0.62**	0.021	ENSG00000080824.19	**-0.42**	0.000
NM_030811	MRPS26	3'UTR	671	protein_coding	**0.62**	0.047	ENSG00000125901.6	**-0.38**	0.006
NM_006139	CD28	CDS	507	protein_coding	**0.62**	0.035	ENSG00000178562.18	**-0.60**	0.006
NM_032689	ZNF607	3'UTR	4026	protein_coding	**0.62**	0.024	ENSG00000198182.13	**-0.61**	0.007
NM_000527	LDLR	3'UTR	3467	protein_coding	**0.62**	0.026	ENSG00000130164.14	**-0.48**	0.037
NM_012145	DTYMK	3'UTR	1024	protein_coding	**0.62**	0.043	ENSG00000168393.13	**-0.63**	0.000
NM_001039770	TMPPE	CDS	1590	protein_coding	**0.62**	0.037	ENSG00000188167.9	**0.38**	0.001
NM_018984	SSH1	3'UTR	3472	protein_coding	**0.62**	0.033	ENSG00000084112.15	**0.57**	0.000
NM_183400	RNF14	CDS	759	protein_coding	**0.62**	0.012	ENSG00000013561.18	**-0.42**	0.013
NM_005762	TRIM28	CDS	2693	protein_coding	**0.62**	0.006	ENSG00000130726.12	**-0.35**	0.003
NM_001014839	NCDN	CDS	509	protein_coding	**0.62**	0.016	ENSG00000020129.16	**-0.35**	0.011
NM_014804	KIAA0753	CDS	757	protein_coding	**0.62**	0.026	ENSG00000198920.11	**0.29**	0.034
NM_015062	PPRC1	CDS	4011	protein_coding	**0.62**	0.029	ENSG00000148840.11	**-0.29**	0.035
NM_018143_2	KLHL11	CDS	1596	protein_coding	**0.62**	0.024	ENSG00000178502.6	**-0.30**	0.025
NM_020857	VPS18	CDS	722	protein_coding	**0.62**	0.042	ENSG00000104142.11	**0.26**	0.047
NM_001278074	COL5A1	3'UTR	5988	protein_coding	**0.61**	0.029	ENSG00000130635.16	**-1.44**	0.004
NM_025054	VCPIP1	CDS	2301	protein_coding	**0.61**	0.042	ENSG00000175073.8	**0.48**	0.000
NM_020786	PDP2	CDS	1319	protein_coding	**0.61**	0.033	ENSG00000172840.7	**-0.73**	0.001
NM_023074	ZNF649	CDS	757	protein_coding	**0.61**	0.027	ENSG00000198093.11	**-0.33**	0.047
NM_003405	YWHAH	CDS	671	protein_coding	**0.61**	0.009	ENSG00000128245.15	**-0.39**	0.002

NR_132117	DNAJC3-DT	313	313	ncRNA	**0.61**	0.045	ENSG00000247400.4	**0.51**	0.008
NM_003633	ENC1	CDS	1833	protein_coding	**0.61**	0.024	ENSG00000171617.15	**0.89**	0.004
NM_015721	GEMIN4	CDS	957	protein_coding	**0.61**	0.038	ENSG00000179409.11	**-0.59**	0.000
NM_001099638	ZNF146	3'UTR	3115	protein_coding	**0.61**	0.023	ENSG00000167635.12	**-0.38**	0.021
NM_182663	RASSF5	3'UTR	1480	protein_coding	**0.61**	0.025	ENSG00000266094.8	**0.29**	0.006
NM_001761	CCNF	CDS	2019	protein_coding	**0.61**	0.039	ENSG00000162063.13	**-0.55**	0.004
NM_052943	TENT5B	3'UTR	1585	protein_coding	**0.61**	0.013	ENSG00000158246.8	**-1.47**	0.015
NM_001270439	ARPC5	CDS	646	protein_coding	**0.61**	0.032	ENSG00000162704.16	**0.53**	0.001
NM_001620	AHNAK	CDS	15745	protein_coding	**0.61**	0.044	ENSG00000124942.14	**-0.51**	0.000
NM_004687	MTMR4	CDS	2653	protein_coding	**0.61**	0.014	ENSG00000108389.9	**0.36**	0.000
NM_016436	PHF20	3'UTR	3741	protein_coding	**0.61**	0.021	ENSG00000025293.17	**0.76**	0.000
NM_000594	TNF	3'UTR	1226	protein_coding	**0.61**	0.036	ENSG00000232810.4	**-0.92**	0.000
NM_024513	FYCO1	CDS	1074	protein_coding	**0.61**	0.013	ENSG00000163820.15	**-0.26**	0.046
NM_020861	ZBTB2	CDS	1687	protein_coding	**0.61**	0.036	ENSG00000181472.5	**-0.37**	0.001
NM_016316	REV1	CDS	843	protein_coding	**0.61**	0.019	ENSG00000135945.10	**0.28**	0.036
NM_001618	PARP1	CDS	2866	protein_coding	**0.60**	0.013	ENSG00000143799.14	**-0.54**	0.000
NM_001353345	SETD1B	CDS	1581	protein_coding	**0.60**	0.007	ENSG00000139718.11	**0.31**	0.021
NM_000127	EXT1	CDS	1502	protein_coding	**0.60**	0.025	ENSG00000182197.12	**0.48**	0.002
NM_144604	ZC3H18	CDS	2998	protein_coding	**0.60**	0.028	ENSG00000158545.16	**0.29**	0.013
NM_000527	LDLR	5'UTR	162	protein_coding	**0.60**	0.029	ENSG00000130164.14	**-0.48**	0.037
NM_001242851	RNF146	CDS	386	protein_coding	**0.60**	0.002	ENSG00000118518.16	**0.60**	0.000
NM_152892	LRWD1	CDS	1958	protein_coding	**0.60**	0.041	ENSG00000161036.13	**0.78**	0.000
NM_006977	ZBTB25	CDS	1054	protein_coding	**0.60**	0.048	ENSG00000089775.12	**-0.44**	0.003
NM_032859	ABHD13	CDS	817	protein_coding	**0.60**	0.042	ENSG00000139826.6	**0.57**	0.002
NM_004284	CHD1L	CDS	2594	protein_coding	**0.60**	0.025	ENSG00000131778.19	**-0.37**	0.005
NM_017588	WDR5	3'UTR	1281	protein_coding	**0.60**	0.025	ENSG00000196363.10	**-0.35**	0.000
NM_001195055	DDIT3	CDS	771	protein_coding	**0.60**	0.049	ENSG00000175197.12	**1.09**	0.000
NM_014071	NCOA6	CDS	2695	protein_coding	**0.60**	0.016	ENSG00000198646.14	**0.34**	0.001

NM_003246	THBS1	3'UTR	4329	protein_coding	**0.60**	0.019	ENSG00000137801.11	**2.12**	0.000
NM_018197	ZFP64	CDS	1752	protein_coding	**0.60**	0.008	ENSG00000020256.20	**-0.70**	0.000
NM_014866	SEC16A	CDS	3392	protein_coding	**0.60**	0.002	ENSG00000148396.18	**0.30**	0.043
NM_001008744	TDP1	CDS	419	protein_coding	**0.60**	0.017	ENSG00000042088.14	**-0.38**	0.022
NM_001025252	TPD52	CDS	528	protein_coding	**0.60**	0.013	ENSG00000076554.15	**-0.90**	0.000
NM_015340	LARS2	3'UTR	3201	protein_coding	**0.60**	0.004	ENSG00000011376.12	**-0.27**	0.023
NM_005647	TBL1X	3'UTR	4582	protein_coding	**0.60**	0.023	ENSG00000101849.17	**0.75**	0.000
NM_004508	IDI1	3'UTR	1682	protein_coding	**0.60**	0.027	ENSG00000067064.12	**0.77**	0.000
NM_001174108	ZBED6	CDS	2783	protein_coding	**0.60**	0.038	ENSG00000257315.3	**0.33**	0.019
NM_181672	OGT	3'UTR	4091	protein_coding	**0.59**	0.033	ENSG00000147162.14	**0.27**	0.000
NM_003390	WEE1	3'UTR	2579	protein_coding	**0.59**	0.035	ENSG00000166483.12	**-0.36**	0.030
NM_001198838	RBM12	3'UTR	3480	protein_coding	**0.59**	0.004	ENSG00000244462.8	**0.26**	0.011
NM_000127	EXT1	CDS	1326	protein_coding	**0.59**	0.003	ENSG00000182197.12	**0.48**	0.002
NM_006598	SLC12A7	3'UTR	4008	protein_coding	**0.59**	0.015	ENSG00000113504.21	**-0.59**	0.010
NM_001244134	MAP3K8	3'UTR	1812	protein_coding	**0.59**	0.006	ENSG00000107968.10	**0.43**	0.000
NM_020414	DDX24	CDS	1154	protein_coding	**0.59**	0.036	ENSG00000089737.17	**-0.29**	0.007
NM_032515	BOK	3'UTR	1861	protein_coding	**0.59**	0.033	ENSG00000176720.6	**-1.40**	0.002
NM_004973	JARID2	CDS	1634	protein_coding	**0.59**	0.030	ENSG00000008083.14	**0.34**	0.003
NM_005650	TCF20	CDS	3552	protein_coding	**0.59**	0.033	ENSG00000100207.20	**0.21**	0.012
NM_006540	NCOA2	CDS	2409	protein_coding	**0.59**	0.035	ENSG00000140396.13	**0.50**	0.001
NM_015963	THAP4	CDS	480	protein_coding	**0.59**	0.029	ENSG00000176946.12	**-0.41**	0.002
NM_001244580	TRRAP	3'UTR	12481	protein_coding	**0.59**	0.025	ENSG00000196367.14	**0.15**	0.018
NM_013248	NXT1	CDS	548	protein_coding	**0.59**	0.027	ENSG00000132661.4	**-0.46**	0.002
NM_001100594	SNRK	CDS	2497	protein_coding	**0.59**	0.042	ENSG00000163788.14	**0.72**	0.000
NM_182919	TICAM1	3'UTR	2563	protein_coding	**0.59**	0.049	ENSG00000127666.10	**-0.47**	0.000
NM_006095	ATP8A1	3'UTR	3858	protein_coding	**0.59**	0.015	ENSG00000124406.16	**0.39**	0.016
NM_001973	ELK4	3'UTR	2681	protein_coding	**0.59**	0.035	ENSG00000158711.14	**-0.36**	0.017
NM_022841	RFX7	CDS	2302	protein_coding	**0.59**	0.020	ENSG00000181827.15	**-0.39**	0.029

NM_153371	LNX2	CDS	1355	protein_coding	**0.59**	0.011	ENSG00000139517.9	**-0.43**	0.000
NM_032752	ZNF496	CDS	2162	protein_coding	**0.59**	0.032	ENSG00000162714.12	**-0.40**	0.014
NM_016565	COA4	CDS	305	protein_coding	**0.59**	0.010	ENSG00000181924.7	**-0.35**	0.025
NR_047573	TMEM44-AS1	63	63	ncRNA	**0.59**	0.034	ENSG00000231770.6	**0.60**	0.007
NM_014757	MAML1	CDS	3258	protein_coding	**0.59**	0.008	ENSG00000161021.13	**0.24**	0.005
NM_006943	SOX12	3'UTR	2486	protein_coding	**0.59**	0.046	ENSG00000177732.9	**-0.76**	0.000
NM_004901	ENTPD4	CDS	1487	protein_coding	**0.59**	0.037	ENSG00000197217.13	**0.24**	0.005
NM_001184900	CARD8	3'UTR	2079	protein_coding	**0.58**	0.016	ENSG00000105483.18	**0.48**	0.000
NM_014908	DOLK	CDS	662	protein_coding	**0.58**	0.027	ENSG00000175283.8	**-0.45**	0.000
NM_001961	EEF2	CDS	1929	protein_coding	**0.58**	0.017	ENSG00000167658.16	**-0.36**	0.001
NM_144498	OSBPL2	3'UTR	2404	protein_coding	**0.58**	0.013	ENSG00000130703.17	**0.70**	0.000
NM_015421	TMEM186	CDS	491	protein_coding	**0.58**	0.017	ENSG00000184857.8	**-0.31**	0.014
NM_004285	H6PD	CDS	344	protein_coding	**0.58**	0.015	ENSG00000049239.13	**0.34**	0.006
NM_001007278	TRIM13	CDS	714	protein_coding	**0.58**	0.036	ENSG00000204977.10	**-0.30**	0.005
NM_001145343	ZNF566	CDS	548	protein_coding	**0.58**	0.009	ENSG00000186017.14	**-0.55**	0.020
NM_001257975	CIZ1	CDS	925	protein_coding	**0.58**	0.025	ENSG00000148337.21	**-0.28**	0.045
NM_012448	STAT5B	CDS	2501	protein_coding	**0.58**	0.045	ENSG00000173757.10	**0.60**	0.000
NM_014506	TOR1B	CDS	1037	protein_coding	**0.58**	0.026	ENSG00000136816.16	**0.84**	0.000
NM_001042631	SDHAF1	3'UTR	767	protein_coding	**0.58**	0.020	ENSG00000205138.4	**-0.59**	0.001
NM_032590	KDM2B	CDS	1692	protein_coding	**0.58**	0.032	ENSG00000089094.19	**-0.42**	0.013
NM_005801	EIF1	3'UTR	874	protein_coding	**0.58**	0.029	ENSG00000173812.11	**-0.31**	0.020
NM_001005333	MAGED1	5'UTR	49	protein_coding	**0.58**	0.007	ENSG00000179222.18	**-0.44**	0.003
NM_014918	CHSY1	CDS	960	protein_coding	**0.58**	0.029	ENSG00000131873.7	**1.05**	0.000
NM_014883	FAM13A	3'UTR	3674	protein_coding	**0.58**	0.038	ENSG00000138640.15	**0.77**	0.001
NM_021922	FANCE	CDS	670	protein_coding	**0.58**	0.006	ENSG00000112039.5	**-0.52**	0.001
NM_022347	TOR1AIP2	5'UTR	333	protein_coding	**0.58**	0.023	ENSG00000169905.13	**0.36**	0.001
NM_170606	KMT2C	CDS	11359	protein_coding	**0.58**	0.044	ENSG00000055609.20	**0.68**	0.000
NM_002473	MYH9	CDS	4214	protein_coding	**0.58**	0.033	ENSG00000100345.22	**0.74**	0.000

NM_014938	MLXIP	3'UTR	3987	protein_coding	**0.57**	0.032	ENSG00000175727.14	**0.24**	0.030
NM_020177	FEM1C	CDS	843	protein_coding	**0.57**	0.026	ENSG00000145780.8	**0.34**	0.024
NM_030937	CCNL2	3'UTR	1714	protein_coding	**0.57**	0.028	ENSG00000221978.12	**0.30**	0.000
NM_015221	DNMBP	CDS	1261	protein_coding	**0.57**	0.010	ENSG00000107554.17	**0.58**	0.000
NM_001946	DUSP6	CDS	1781	protein_coding	**0.57**	0.042	ENSG00000139318.8	**-0.69**	0.000
NM_012433	SF3B1	5'UTR	89	protein_coding	**0.57**	0.046	ENSG00000115524.17	**0.59**	0.000
NM_199360	TPD52L2	CDS	178	protein_coding	**0.57**	0.038	ENSG00000101150.18	**0.72**	0.000
NM_000875	IGF1R	3'UTR	10131	protein_coding	**0.57**	0.039	ENSG00000140443.15	**0.88**	0.000
NM_015100	POGZ	CDS	4429	protein_coding	**0.57**	0.013	ENSG00000143442.22	**0.50**	0.000
NR_131012	NEAT1	933	933	ncRNA	**0.57**	0.027	ENSG00000245532.9	**1.34**	0.000
NM_001029882	AHDC1	CDS	3761	protein_coding	**0.57**	0.024	ENSG00000126705.15	**-0.72**	0.000
NM_018706	DHTKD1	3'UTR	3278	protein_coding	**0.57**	0.044	ENSG00000181192.12	**0.44**	0.004
NM_021035	ZNFX1	3'UTR	6839	protein_coding	**0.57**	0.026	ENSG00000124201.15	**0.56**	0.000
NM_175907	ZADH2	3'UTR	1948	protein_coding	**0.57**	0.014	ENSG00000180011.7	**-0.45**	0.001
NM_024735	FBXO31	3'UTR	1894	protein_coding	**0.57**	0.014	ENSG00000103264.18	**-0.49**	0.008
NM_001257975	CIZ1	CDS	1293	protein_coding	**0.57**	0.031	ENSG00000148337.21	**-0.28**	0.045
NM_018948	ERRFI1	CDS	1610	protein_coding	**0.57**	0.015	ENSG00000116285.13	**1.03**	0.007
NM_000179	MSH6	CDS	2834	protein_coding	**0.57**	0.005	ENSG00000116062.17	**-0.32**	0.013
NM_181672	OGT	CDS	2967	protein_coding	**0.56**	0.015	ENSG00000147162.14	**0.27**	0.000
NM_015629	PRPF31	CDS	510	protein_coding	**0.56**	0.009	ENSG00000105618.14	**-0.39**	0.001
NM_152260	RPUSD2	CDS	177	protein_coding	**0.56**	0.038	ENSG00000166133.18	**-0.43**	0.012
NM_013248	NXT1	3'UTR	892	protein_coding	**0.56**	0.038	ENSG00000132661.4	**-0.46**	0.002
NM_001007278	TRIM13	CDS	674	protein_coding	**0.56**	0.034	ENSG00000204977.10	**-0.30**	0.005
NM_017780	CHD7	CDS	1852	protein_coding	**0.56**	0.039	ENSG00000171316.12	**1.04**	0.000
NM_001821	CHML	3'UTR	2201	protein_coding	**0.56**	0.042	ENSG00000203668.3	**-0.48**	0.005
NM_001173487	NKRF	CDS	2087	protein_coding	**0.56**	0.038	ENSG00000186416.17	**-0.35**	0.013
NM_014844	TECPR2	CDS	2162	protein_coding	**0.56**	0.017	ENSG00000196663.16	**1.05**	0.000
NM_014866	SEC16A	CDS	3274	protein_coding	**0.56**	0.012	ENSG00000148396.18	**0.30**	0.043

NM_001012241	MSL1	CDS	1183	protein_coding	**0.55**	0.019	ENSG00000188895.12	**0.70**	0.000
NM_001244950	SPOCK2	5'UTR	393	protein_coding	**0.55**	0.038	ENSG00000107742.13	**-0.34**	0.031
NM_025222	WDR82	5'UTR	26	protein_coding	**0.55**	0.040	ENSG00000164091.12	**-0.15**	0.008
NM_019042	PUS7	CDS	546	protein_coding	**0.55**	0.013	ENSG00000091127.14	**0.78**	0.036
NM_020829	RIC1	CDS	4306	protein_coding	**0.55**	0.036	ENSG00000107036.12	**0.27**	0.019
NM_178014_6	TUBB	3'UTR	2414	protein_coding	**0.55**	0.024	ENSG00000196230.14	**-0.53**	0.000
NM_006309	LRRFIP2	3'UTR	2708	protein_coding	**0.55**	0.010	ENSG00000093167.18	**0.42**	0.001
NM_013386	SLC25A24	3'UTR	1730	protein_coding	**0.55**	0.024	ENSG00000085491.17	**-0.22**	0.031
NM_001136262	ATXN7L3B	3'UTR	1289	protein_coding	**0.55**	0.012	ENSG00000253719.4	**-0.31**	0.001
NM_001077199	SREK1	CDS	2019	protein_coding	**0.55**	0.027	ENSG00000153914.16	**0.21**	0.026
NM_031444	GUCD1	3'UTR	3587	protein_coding	**0.55**	0.049	ENSG00000138867.17	**-0.51**	0.003
NM_020337	ANKRD50	CDS	2294	protein_coding	**0.55**	0.002	ENSG00000151458.12	**0.35**	0.034
NM_004719	SCAF11	CDS	4668	protein_coding	**0.55**	0.013	ENSG00000139218.18	**0.37**	0.001
NM_001256183	ANKRD11	CDS	3024	protein_coding	**0.55**	0.039	ENSG00000167522.16	**0.63**	0.000
NM_001126111	OSGIN2	CDS	1521	protein_coding	**0.54**	0.008	ENSG00000164823.11	**0.47**	0.007
NM_014982	PCNX1	CDS	1718	protein_coding	**0.54**	0.012	ENSG00000100731.16	**1.03**	0.000
NM_001300	KLF6	CDS	535	protein_coding	**0.54**	0.030	ENSG00000067082.15	**0.61**	0.000
NM_004897	MINPP1	CDS	1739	protein_coding	**0.54**	0.012	ENSG00000107789.16	**-0.57**	0.000
NM_014883	FAM13A	3'UTR	3708	protein_coding	**0.54**	0.008	ENSG00000138640.15	**0.77**	0.001
NM_005902	SMAD3	3'UTR	1725	protein_coding	**0.54**	0.011	ENSG00000166949.17	**-0.34**	0.042
NM_012405	ICMT	3'UTR	2126	protein_coding	**0.54**	0.040	ENSG00000116237.16	**-0.54**	0.000
NM_014883	FAM13A	3'UTR	4010	protein_coding	**0.54**	0.023	ENSG00000138640.15	**0.77**	0.001
NM_032689	ZNF607	CDS	925	protein_coding	**0.54**	0.040	ENSG00000198182.13	**-0.61**	0.007
NM_152260	RPUSD2	CDS	156	protein_coding	**0.54**	0.030	ENSG00000166133.18	**-0.43**	0.012
NM_133647	SLC12A6	3'UTR	4108	protein_coding	**0.54**	0.036	ENSG00000140199.13	**0.63**	0.000
NM_018717	MAML3	CDS	1440	protein_coding	**0.54**	0.049	ENSG00000196782.12	**0.49**	0.001
NM_002712	PPP1R7	CDS	939	protein_coding	**0.54**	0.038	ENSG00000115685.15	**-0.28**	0.006
NM_197968	ZMYM2	CDS	4353	protein_coding	**0.53**	0.012	ENSG00000121741.17	**0.45**	0.001

NM_001242739	ZNF691	CDS	1171	protein_coding	**0.53**	0.025	ENSG00000164011.18	**-0.38**	0.008
NM_031217	KIF18A	CDS	2467	protein_coding	**0.53**	0.027	ENSG00000121621.7	**-0.58**	0.015
NM_003463	PTP4A1	5'UTR	861	protein_coding	**0.53**	0.049	ENSG00000112245.12	**0.68**	0.000
NM_002766	PRPSAP1	3'UTR	1771	protein_coding	**0.53**	0.044	ENSG00000161542.17	**-0.46**	0.000
NM_006775	QKI	3'UTR	3922	protein_coding	**0.53**	0.037	ENSG00000112531.17	**0.46**	0.001
NM_022347	TOR1AIP2	5'UTR	682	protein_coding	**0.53**	0.016	ENSG00000169905.13	**0.36**	0.001
NM_152705	POLR1D	CDS	396	protein_coding	**0.52**	0.041	ENSG00000186184.19	**-0.55**	0.000
NM_170606	KMT2C	CDS	6273	protein_coding	**0.52**	0.034	ENSG00000055609.20	**0.68**	0.000
NM_001144831	PHB2	3'UTR	1331	protein_coding	**0.52**	0.009	ENSG00000215021.10	**-0.16**	0.045
NM_004850	ROCK2	3'UTR	4670	protein_coding	**0.52**	0.050	ENSG00000134318.14	**0.35**	0.012
NM_032199	ARID5B	3'UTR	3970	protein_coding	**0.52**	0.013	ENSG00000150347.17	**0.43**	0.001
NM_001002860	BTBD7	CDS	3381	protein_coding	**0.52**	0.010	ENSG00000011114.15	**0.28**	0.004
NM_001111	ADAR	CDS	360	protein_coding	**0.52**	0.024	ENSG00000160710.18	**0.51**	0.000
NM_001102426	TBC1D8	CDS	3451	protein_coding	**0.52**	0.034	ENSG00000204634.13	**0.74**	0.001
NM_006923	SDF2	CDS	748	protein_coding	**0.52**	0.042	ENSG00000132581.10	**0.38**	0.006
NM_001278196	ZBTB18	CDS	484	protein_coding	**0.52**	0.015	ENSG00000179456.10	**0.50**	0.000
NM_001040694	INCENP	CDS	1144	protein_coding	**0.52**	0.037	ENSG00000149503.13	**-0.38**	0.023
NM_021964	ZNF148	CDS	558	protein_coding	**0.52**	0.007	ENSG00000163848.20	**0.30**	0.029
NM_002094	GSPT1	3'UTR	5490	protein_coding	**0.52**	0.031	ENSG00000103342.13	**-0.68**	0.002
NM_033317	DMKN	3'UTR	1858	protein_coding	**0.51**	0.006	ENSG00000161249.21	**-1.57**	0.006
NM_001134338	RNF24	CDS	667	protein_coding	**0.51**	0.022	ENSG00000101236.17	**0.62**	0.001
NM_015312	KIAA1109	CDS	4373	protein_coding	**0.51**	0.044	ENSG00000138688.16	**0.87**	0.000
NM_022455	NSD1	CDS	7306	protein_coding	**0.51**	0.040	ENSG00000165671.21	**0.44**	0.000
NM_015340	LARS2	3'UTR	2995	protein_coding	**0.51**	0.039	ENSG00000011376.12	**-0.27**	0.023
NM_015060	AVL9	CDS	1327	protein_coding	**0.51**	0.040	ENSG00000105778.19	**0.40**	0.002
NM_145034	TOR1AIP2	CDS	1604	protein_coding	**0.51**	0.036	ENSG00000169905.13	**0.36**	0.001
NM_001099679	TRIM32	3'UTR	2343	protein_coding	**0.51**	0.029	ENSG00000119401.11	**-0.51**	0.001
NM_005779	LHFPL2	3'UTR	1768	protein_coding	**0.51**	0.021	ENSG00000145685.14	**0.35**	0.018

NM_024805	RBFA	CDS	1017	protein_coding	**0.51**	0.035	ENSG00000101546.13	**-0.63**	0.002
NM_006766	KAT6A	CDS	6143	protein_coding	**0.51**	0.048	ENSG00000083168.11	**0.49**	0.000
NM_016332	MSRB1	3'UTR	956	protein_coding	**0.51**	0.021	ENSG00000198736.12	**0.79**	0.000
NM_001080495	TNRC18	CDS	5433	protein_coding	**0.50**	0.008	ENSG00000182095.14	**0.53**	0.001
NM_001111	ADAR	3'UTR	3873	protein_coding	**0.50**	0.023	ENSG00000160710.18	**0.51**	0.000
NM_198935	SS18L1	3'UTR	4143	protein_coding	**0.50**	0.004	ENSG00000184402.15	**0.26**	0.045
NM_033204	ZNF101	CDS	983	protein_coding	**0.50**	0.033	ENSG00000181896.12	**-0.40**	0.010
NM_021738	SVIL	CDS	1354	protein_coding	**0.50**	0.032	ENSG00000197321.15	**0.92**	0.000
NM_001017995	SH3PXD2B	3'UTR	6508	protein_coding	**0.50**	0.020	ENSG00000174705.13	**0.71**	0.049
NM_015556	SIPA1L1	CDS	4819	protein_coding	**0.50**	0.035	ENSG00000197555.9	**0.98**	0.000
NM_001961	EEF2	CDS	242	protein_coding	**0.50**	0.033	ENSG00000167658.16	**-0.36**	0.001
NM_025191	EDEM3	CDS	2818	protein_coding	**0.50**	0.035	ENSG00000116406.19	**0.43**	0.001
NM_001013694	SRRD	CDS	918	protein_coding	**0.50**	0.025	ENSG00000100104.13	**-0.58**	0.015
NM_001278346	PHYKPL	5'UTR	1027	protein_coding	**0.50**	0.019	ENSG00000175309.15	**0.41**	0.000
NM_017840	MRPL16	CDS	469	protein_coding	**0.49**	0.039	ENSG00000166902.5	**-0.34**	0.005
NM_024832	RIN3	3'UTR	3204	protein_coding	**0.49**	0.022	ENSG00000100599.16	**0.48**	0.000
NM_006379	SEMA3C	CDS	2576	protein_coding	**0.49**	0.041	ENSG00000075223.14	**1.04**	0.000
NM_001190945	TRAF1	3'UTR	2960	protein_coding	**0.49**	0.009	ENSG00000056558.11	**-0.59**	0.000
NM_015172	PRRC2C	CDS	1756	protein_coding	**0.49**	0.036	ENSG00000117523.16	**0.37**	0.001
NM_012158	FBXL3	CDS	1358	protein_coding	**0.49**	0.044	ENSG00000005812.11	**0.26**	0.021
NM_020747	ZNF608	CDS	3554	protein_coding	**0.48**	0.038	ENSG00000168916.15	**1.24**	0.000
NM_004719	SCAF11	CDS	2958	protein_coding	**0.48**	0.039	ENSG00000139218.18	**0.37**	0.001
NM_001244580	TRRAP	CDS	11715	protein_coding	**0.48**	0.025	ENSG00000196367.14	**0.15**	0.018
NM_002473	MYH9	3'UTR	6397	protein_coding	**0.47**	0.042	ENSG00000100345.22	**0.74**	0.000
NM_017542	POGK	CDS	1092	protein_coding	**0.47**	0.035	ENSG00000143157.12	**-0.32**	0.000
NM_012481	IKZF3	3'UTR	2071	protein_coding	**0.47**	0.037	ENSG00000161405.17	**-0.40**	0.022
NM_004566	PFKFB3	3'UTR	2728	protein_coding	**0.47**	0.032	ENSG00000170525.21	**1.15**	0.000
NM_018385	LSG1	3'UTR	2832	protein_coding	**0.47**	0.011	ENSG00000041802.11	**-0.42**	0.011

NM_004285	H6PD	3'UTR	2791	protein_coding	**0.46**	0.035	ENSG00000049239.13	**0.34**	0.006
NM_152857	WTAP	CDS	668	protein_coding	**0.46**	0.037	ENSG00000146457.16	**0.41**	0.000
NM_007373	SHOC2	3'UTR	3529	protein_coding	**0.46**	0.012	ENSG00000108061.12	**0.54**	0.003
NM_001136262	ATXN7L3B	3'UTR	732	protein_coding	**0.45**	0.045	ENSG00000253719.4	**-0.31**	0.001
NM_002229	JUNB	CDS	1250	protein_coding	**0.45**	0.008	ENSG00000171223.6	**0.40**	0.031
NM_004728	DDX21	CDS	362	protein_coding	**0.45**	0.040	ENSG00000165732.13	**-0.32**	0.025
NM_138704	NSMCE3	3'UTR	1052	protein_coding	**0.45**	0.041	ENSG00000185115.6	**-0.35**	0.008
NR_023924	DHRS4-AS1	1444	1444	ncRNA	**0.44**	0.023	ENSG00000215256.4	**-0.53**	0.000
NM_016604	KDM3B	CDS	1329	protein_coding	**0.44**	0.036	ENSG00000120733.14	**0.76**	0.000
NM_001172818	PGM1	CDS	565	protein_coding	**0.44**	0.033	ENSG00000079739.17	**0.52**	0.000
NM_001199862	KCNAB2	3'UTR	1695	protein_coding	**0.44**	0.033	ENSG00000069424.15	**0.28**	0.001
NM_017802	DNAAF5	3'UTR	3075	protein_coding	**0.43**	0.041	ENSG00000164818.16	**-0.64**	0.000
NM_018380	DDX28	3'UTR	2321	protein_coding	**0.43**	0.032	ENSG00000182810.7	**-0.26**	0.016
NM_004728	DDX21	CDS	305	protein_coding	**0.43**	0.044	ENSG00000165732.13	**-0.32**	0.025
NM_017934	PHIP	CDS	5393	protein_coding	**0.42**	0.007	ENSG00000146247.14	**0.51**	0.000
NM_024490	ATP10A	CDS	4500	protein_coding	**0.42**	0.041	ENSG00000206190.12	**-0.42**	0.009
NM_001002762	DNAJB12	3'UTR	2371	protein_coding	**0.41**	0.007	ENSG00000148719.15	**0.44**	0.000
NM_001206	KLF9	3'UTR	2090	protein_coding	**0.40**	0.033	ENSG00000119138.4	**0.63**	0.000
NM_021964	ZNF148	CDS	2425	protein_coding	**0.39**	0.047	ENSG00000163848.20	**0.30**	0.029
NM_005230	ELK3	3'UTR	1633	protein_coding	**0.39**	0.009	ENSG00000111145.8	**-0.28**	0.009
NM_006923	SDF2	CDS	849	protein_coding	**0.39**	0.020	ENSG00000132581.10	**0.38**	0.006
NM_021181	SLAMF7	3'UTR	2007	protein_coding	**0.38**	0.024	ENSG00000026751.17	**-0.57**	0.012
NM_001178099	ZNF182	3'UTR	2583	protein_coding	**0.38**	0.004	ENSG00000147118.12	**0.24**	0.035
NM_012482	ZNF281	3'UTR	3361	protein_coding	**0.37**	0.035	ENSG00000162702.8	**1.43**	0.000
NM_002706	PPM1B	CDS	796	protein_coding	**0.36**	0.046	ENSG00000138032.21	**0.34**	0.002
NM_058180	C21orf58	5'UTR	615	protein_coding	**0.34**	0.020	ENSG00000160298.18	**-0.43**	0.033
NM_024963	FBXL18	CDS	1581	protein_coding	**0.33**	0.028	ENSG00000155034.19	**0.33**	0.020
NM_024095	ASB8	CDS	770	protein_coding	**0.32**	0.046	ENSG00000177981.11	**0.30**	0.003

NM_152680	TMEM154	3'UTR	2002	protein_coding	**0.32**	0.032	ENSG00000170006.12	**0.48**	0.002
NM_001172669	ZNF668	3'UTR	2432	protein_coding	**0.31**	0.044	ENSG00000167394.13	**-0.41**	0.003
NM_001728	BSG	CDS	870	protein_coding	**0.31**	0.022	ENSG00000172270.22	**-0.47**	0.014
NM_001174118	MEX3D	CDS	734	protein_coding	**0.30**	0.024	ENSG00000181588.16	**-0.59**	0.004
NM_080598	DDX39B	CDS	547	protein_coding	**0.29**	0.043	ENSG00000198563.14	**0.16**	0.017
NM_001039999	FAM83G	CDS	1798	protein_coding	**0.28**	0.048	ENSG00000188522.15	**-0.75**	0.000
NM_001012991	KNOP1	3'UTR	1551	protein_coding	**0.26**	0.023	ENSG00000103550.14	**-0.61**	0.001
NM_004375	COX11	CDS	265	protein_coding	**0.25**	0.031	ENSG00000166260.13	**-0.52**	0.009
NM_015659	RSL1D1	CDS	1086	protein_coding	**0.25**	0.045	ENSG00000171490.13	**-0.35**	0.045
**m6A methylation array (hypemethylated)**							**RNAsequensing**		
**TransID**	**Gene Symbol**	**m6A location**	**m6A transcript location**	**Trans biotype**	**Fold change**	**P-value (unpaired t-test)**	Gene ID Version	**log2 Fold Change**	p-value
NM_001017963	HSP90AA1	CDS	829	protein_coding	**3.47**	0.018	ENSG00000080824.19	**-0.42**	0.000
NM_001134484	TOMM5	3'UTR	529	protein_coding	**3.11**	0.028	ENSG00000175768.13	**-0.36**	0.018
NM_005082	TRIM25	3'UTR	5556	protein_coding	**2.97**	0.048	ENSG00000121060.18	**0.72**	0.000
NM_005541	INPP5D	3'UTR	4410	protein_coding	**2.70**	0.019	ENSG00000168918.14	**0.68**	0.000
NM_015443_2	KANSL1	CDS	1357	protein_coding	**2.58**	0.003	ENSG00000120071.15	**0.40**	0.002
NR_131012	NEAT1	1610	1610	ncRNA	**2.54**	0.033	ENSG00000245532.9	**1.34**	0.000
NM_012482	ZNF281	CDS	770	protein_coding	**2.43**	0.025	ENSG00000162702.8	**1.43**	0.000
NM_015960	CUTC	CDS	452	protein_coding	**2.42**	0.005	ENSG00000119929.13	**0.36**	0.000
NM_002296	LBR	3'UTR	3656	protein_coding	**2.35**	0.033	ENSG00000143815.15	**1.11**	0.000
NM_006825	CKAP4	CDS	1756	protein_coding	**2.26**	0.036	ENSG00000136026.14	**1.25**	0.000
NM_006825	CKAP4	CDS	1798	protein_coding	**2.19**	0.038	ENSG00000136026.14	**1.25**	0.000
NM_052947	ALPK2	CDS	5376	protein_coding	**2.09**	0.033	ENSG00000198796.7	**1.25**	0.023
NM_145055	C18orf25	CDS	986	protein_coding	**2.09**	0.047	ENSG00000152242.11	**0.31**	0.000
NM_001277817	YTHDF3	CDS	1575	protein_coding	**2.07**	0.029	ENSG00000185728.17	**0.35**	0.001
NM_024607	PPP1R3B	3'UTR	2317	protein_coding	**1.92**	0.027	ENSG00000173281.5	**0.75**	0.000
NM_032479	MRPL36	3'UTR	582	protein_coding	**1.86**	0.043	ENSG00000171421.13	**-0.50**	0.043

NM_001017963	HSP90AA1	CDS	1337	protein_coding	**1.85**	0.035	ENSG00000080824.19	**-0.42**	0.000
NM_018717	MAML3	3'UTR	4811	protein_coding	**1.85**	0.027	ENSG00000196782.12	**0.49**	0.001
NM_012414	RAB3GAP2	3'UTR	4956	protein_coding	**1.75**	0.032	ENSG00000118873.16	**0.35**	0.007
NM_017926	GPATCH2L	CDS	705	protein_coding	**1.74**	0.037	ENSG00000089916.18	**0.17**	0.015
NM_020755	SERINC1	CDS	1387	protein_coding	**1.71**	0.023	ENSG00000111897.7	**0.47**	0.001
NM_014159	SETD2	CDS	4140	protein_coding	**1.60**	0.045	ENSG00000181555.21	**0.50**	0.000
NM_012336	NARF	3'UTR	1523	protein_coding	**1.59**	0.030	ENSG00000141562.18	**0.52**	0.003

Supplemental table S4. methylated transcripts annotated to the two pathways with highest significantly different between Tocilizumab and Placebo group.

**R-HSA-6798695 Neutrophil degranulation**			**GO:0051301 Cell Division**		
Gene symbol	***Fold Change***	***Pval (unpaired ttest)***	***Gene symbol***	***Fold Change***	***Pval (unpaired ttest)***
ADAM10	0.54	*0.018*	CDC25A	0.52	*0.008*
CD14	0.61	*0.012*	LIG3	0.66	*0.047*
CSTB	0.64	*0.032*	MAP4	0.50	*0.001*
GDI2	0.61	*0.048*	PLEC	2.71	*0.034*
HLA-B	0.63	*0.014*	PLK1	0.40	*0.023*
MANBA	0.41	*0.014*	TOP2A	0.42	*0.035*
DNAJC3	0.60	*0.046*	BUB3	0.65	*0.040*
PSAP	0.54	*0.031*	KIF20A	0.52	*0.018*
PTPRC	0.52	*0.024*	MAEA	0.56	*0.020*
TLR2	0.55	*0.015*	TUBA1B	0.37	*0.019*
KCNAB2	0.54	*0.009*	ABRAXAS2	0.61	*0.005*
CDK13	0.52	*0.026*	EXOC7	0.62	*0.015*
ARPC5	0.56	*0.035*	PELO	0.60	*0.030*
FGL2	0.56	*0.005*	NDE1	0.45	*0.000*
CKAP4	0.43	*0.032*	ERCC6L	0.59	*0.036*
TMEM30A	0.44	*0.039*	CHMP1B	0.46	*0.013*
TUBB	0.54	*0.033*	TUBB	0.54	*0.033*
