# Supplementary material for: The N-6 methyladenosine dynamics in STEMI and the effect of IL-6 inhibition - a hypothesis generating sub-study of the ASSAIL-MI trial
Source: Front Immunol. 2025 Jun 6;16:1532325. doi: 10.3389/fimmu.2025.1532325 (PMC12178860; doi:10.3389/fimmu.2025.1532325)
Supplement: Supplementary file 2 [file DataSheet2.pdf]

Supplemental Figure S1

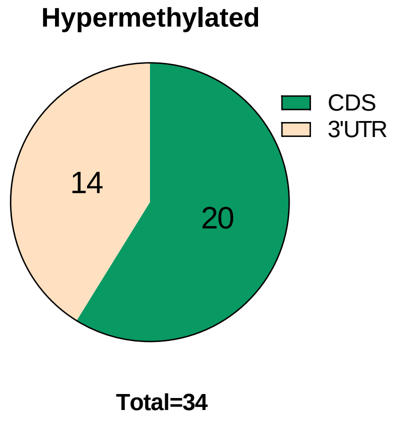

Supplemental Figure S1: m^{6}A distribution in patients with STEMI prior to PCI vs healthy controls. Distribution of regulated hypermethylated m^{6}A sites in mRNAs between patients with STEMI at hospitalization and healthy controls.

Supplemental Figure S2

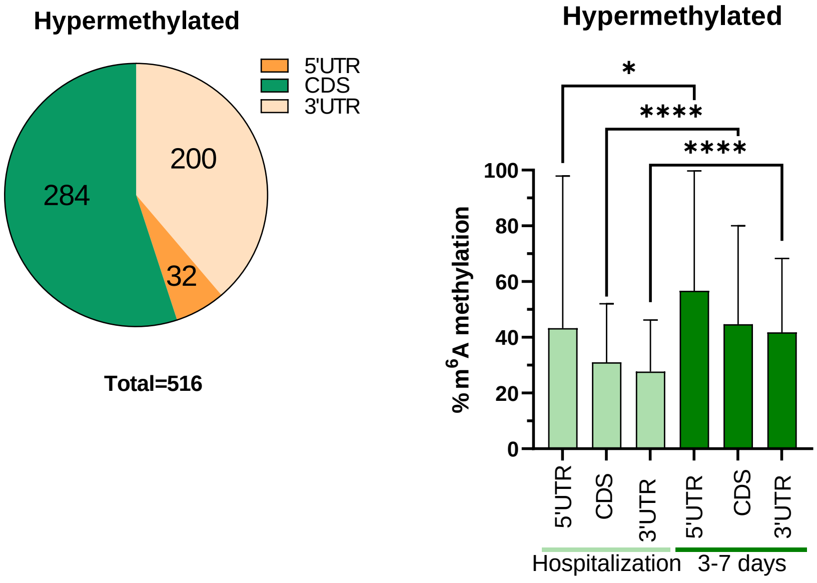

Supplemental Figure S2: m^{6}A distribution in patients with STEMI treated with placebo 3-7 days post hospitalization versus at hospitalization. Distribution of regulated hypermethylated m^{6}A sites in mRNAs between patients with STEMI(placebo) at 3-7 days following infarct versus hospitalization and

average m^{6}A methylation percent for all sites in the different sections of protein coding transcripts. ****p < 0.0001, *p < 0.05 (2-way ANOVA with Tukey's multiple comparisons test).

Supplemental Figure S3

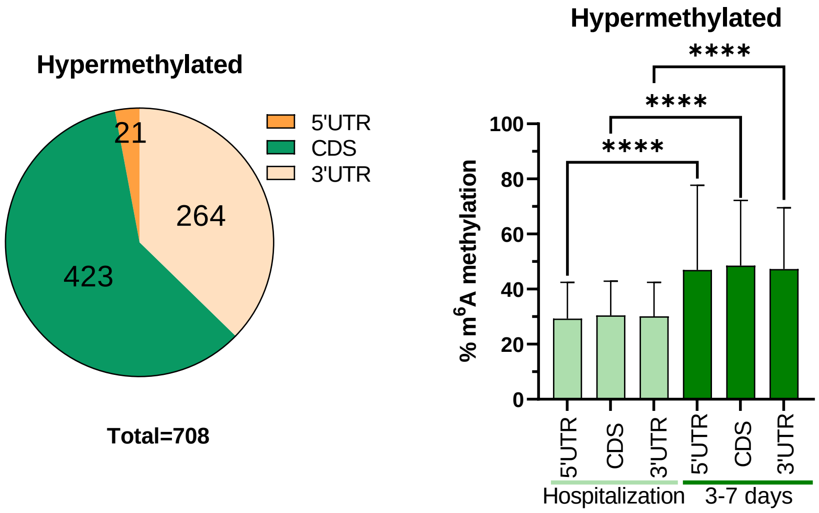

Supplemental Figure S3: m^{6}A distribution in patients with STEMI treated with tocilizumab 3-7 days post hospitalization versus at hospitalization:. Distribution of regulated hypermethylated m^{6}A sites in mRNAs between patients with STEMI(tocilizumab) at 3-7 days following infarct versus hospitalization and average m^{6}A methylation percent for all sites in the different sections of protein coding transcripts. ****p < 0.0001 (2-way ANOVA with Tukey's multiple comparisons test).

Supplemental Figure S4

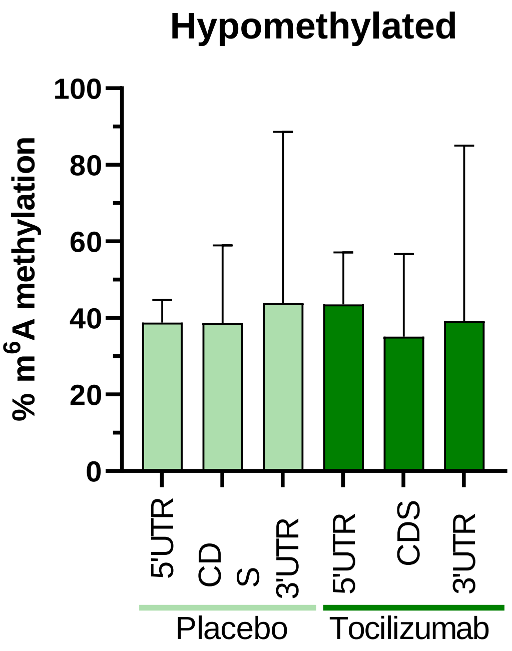

Supplemental Figure S4: m^{6}A distribution in patients with STEMI treated with tocilizumab 3-7 days post hospitalization versus placebo treated patients at day 3-7 post hospitalization. Average m^{6}A methylation percent for all sites in the different sections of protein coding transcripts. *$p < 0.05$ (2-way ANOVA with Tukey's multiple comparisons test).

Supplemental figure S5

A

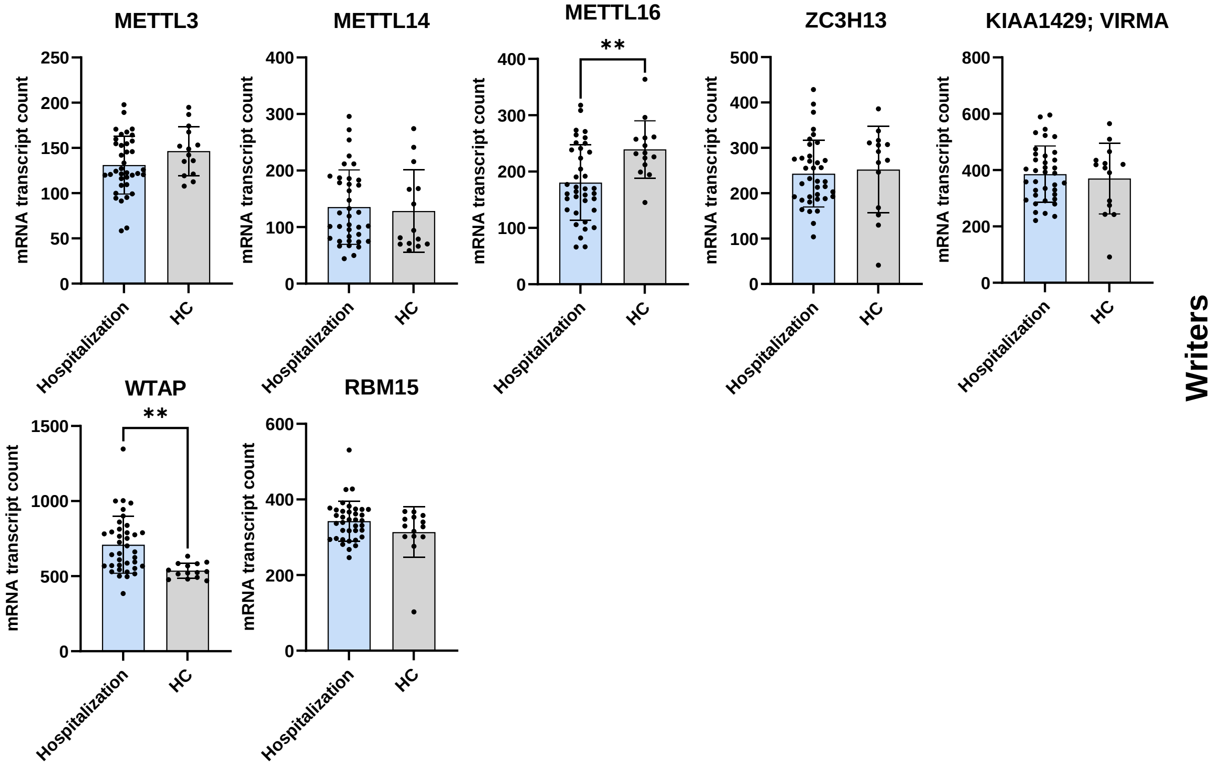

B

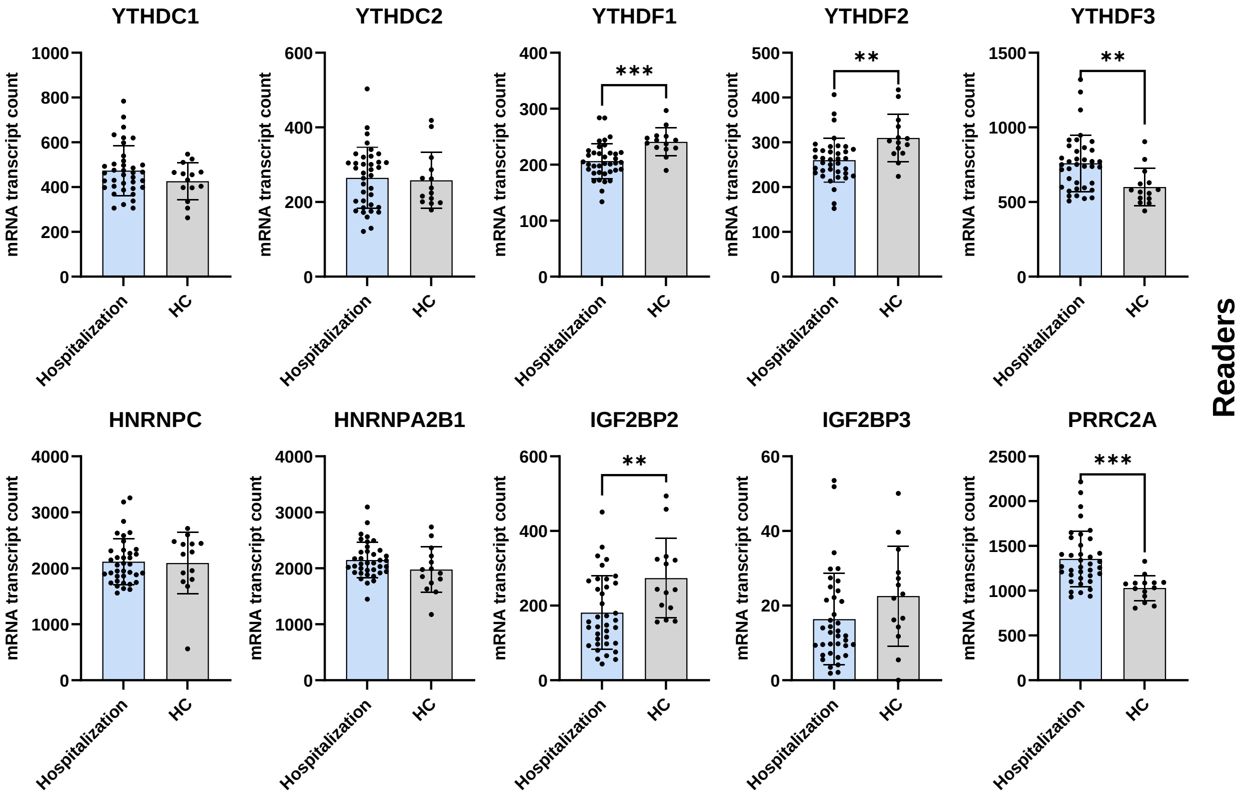

Supplemental figure S6

A

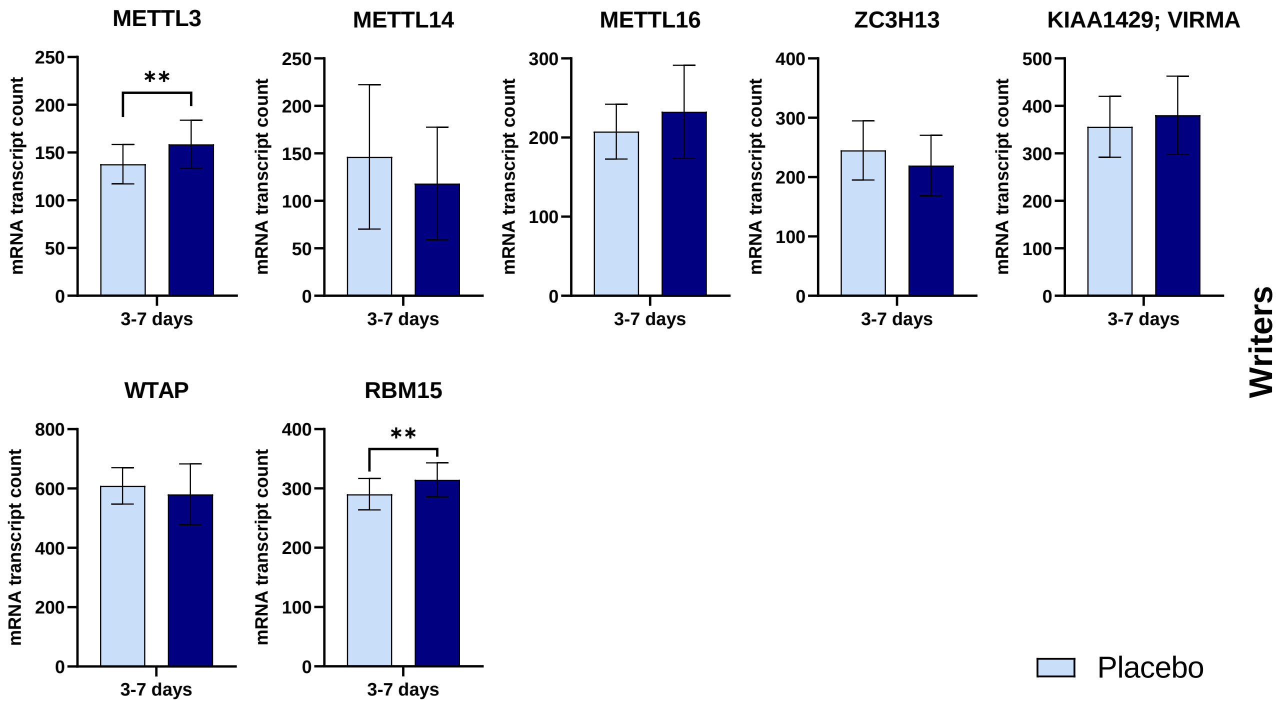

B

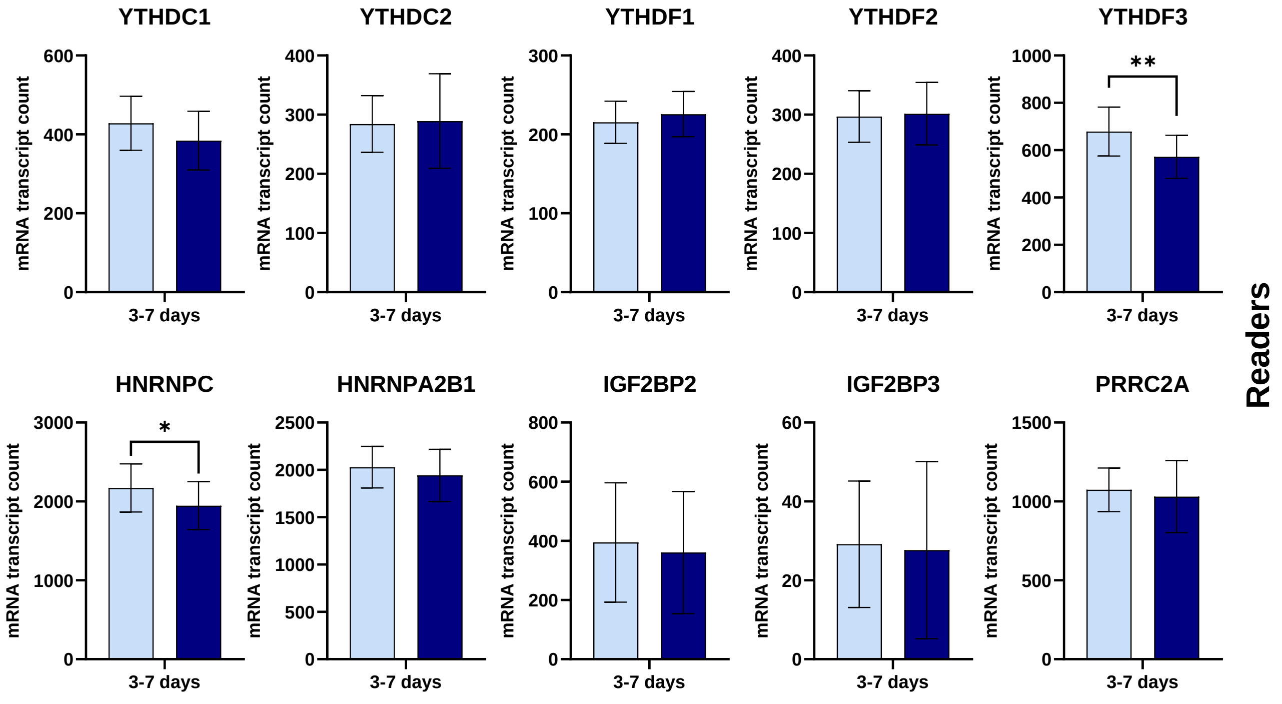

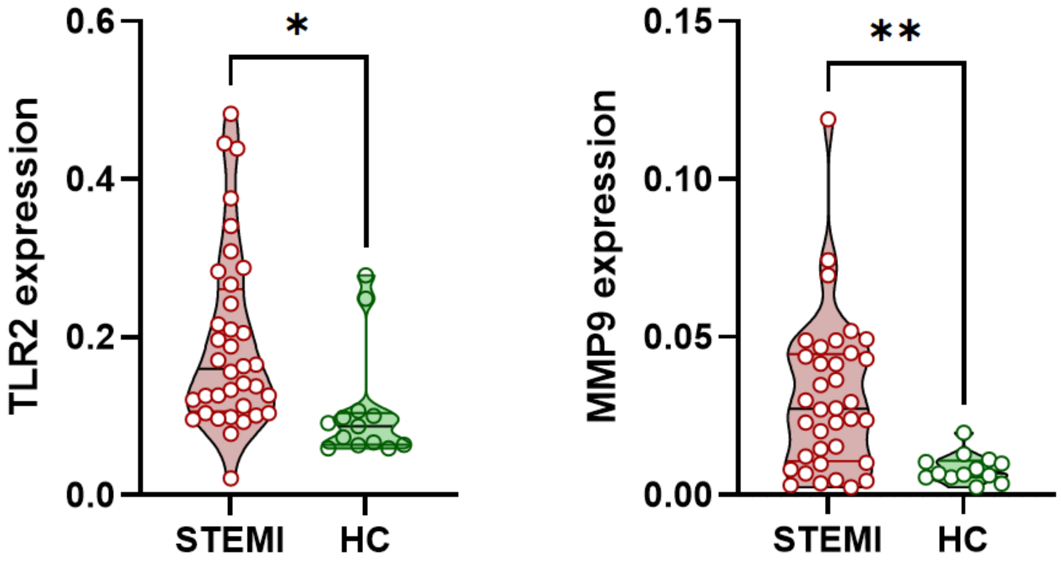

Supplemental figure S7: Increased expression of key proteins in the NFkB Pathway in RNA isolated from whole blood from patients with STEMI compared to healthy controls. *=p<0.05, **p<0.005
